# Temporal changes of headwater river surface microlayer characteristics during the dry to wet season transition in the tropical rainforest of Guyana

**DOI:** 10.1038/s41598-025-28843-4

**Published:** 2025-11-28

**Authors:** Sevda Norouzi, James Spray, Sara Trojahn, Juliane Bischoff, Julian Brasche, Thomas Wagner, Walter Hill, Alan MacDonald, Ryan Pereira

**Affiliations:** 1 The Lyell Centre, Heriot-Watt University, Edinburgh, UK; 2 Iwokrama International Centre for Rainforest Conservation and Development, Georgetown, Guyana; 3 British Geological Survey, Edinburgh, UK; 4 Present Address: The James Hutton Institute, Aberdeen, UK

**Keywords:** Surface microlayer, Headwater river, Tropical rainforest, Dissolved organic matter, Wet season, Dry season, Biogeochemistry, Climate sciences, Ecology, Ecology, Environmental sciences, Hydrology

## Abstract

The surface microlayer (SML) is a fundamental control of energy transfer, biogeochemical processes, and climate-active gases flux at the water-atmosphere interface. Organic matter in the SML has an important role in marine environments but inland waters have received far less attention. This results in large unknowns of SML variability and its impact on boundary layer exchanges. In this study, we investigate variations in dissolved organic carbon (DOC) and dissolved organic matter (DOM) composition in SML and subsurface water (SSW) using size exclusion chromatography in a Northern Amazonia rainforest headwater during the dry to wet season transition. During the dry season, we observed higher DOC concentrations with greater DOM compositional variability, characterised by lower humic substance (HS) abundance and elevated contributions of biopolymers (BP), building blocks (BB), and low-molecular-weight neutrals and acids (LMWN, LMWA). River discharge and DOC flux increased in the wet season. However, the overall DOC concentration decreased accompanied by DOM homogenisation, a dominance of HS, and the disappearance of LMWA. The SML and SSW exhibited similar DOC concentrations but distinct compositional differences, evidenced by divergent HS-DOC relationships and HS nominal molecular weight (M_n_) characteristics. In the dry season, high M_n_ variability in the SML suggested preferential accumulation of higher-molecular-weight HS, which diminished as wet-season conditions stabilised DOM distribution. Enrichment factors (EFs) for all DOM compound groups between the SML and SSW displayed strong temporal variability. These patterns likely reflect shifts in hydrodynamic regimes, terrestrial inputs, and microbial or photochemical processing. Notably, rainfall events did likely not directly trigger EF pulses but indirectly influenced DOM composition from local sources through terrestrial mobilisation. Our results demonstrate that tropical river DOM dynamics are fundamentally controlled by seasonal hydrological regimes, with discharge-driven processes exerting stronger influence on molecular composition and vertical heterogeneity (particularly in the SML) than direct rainfall effects. This hydrological dominance shapes DOM distribution through three interconnected mechanisms: source variability during low-flow periods, transport-mediated homogenisation during high discharge, and in situ processing that differentially affects boundary layer composition.

**Supplementary Information:**

The online version contains supplementary material available at 10.1038/s41598-025-28843-4.

## Introduction

The interface between the atmosphere and hydrosphere, known as the surface microlayer (SML), covers about 70% of the Earth’s surface. The SML is an important biological habitat and a collection point for buoyant particles and dissolved components from the underlying water^1–5^. Compared to subsurface water (SSW), the SML is subject to a broad set of unique physical and chemical properties^6–8^ that are vulnerable to environmental and climatic variations^1^. However, to date, the majority of SML characterisations have been focused on marine systems, with relatively few investigations of freshwater systems^9–11^ and a notable paucity in tropical rivers. Organic matter compositional differences between SML and SSW have been shown to play a key role in the water-atmosphere transfer of climate active gases including carbon dioxide (CO_2_), methane (CH_4_) and nitrous oxide (N_2_O) in marine systems^3,12^. However, SML processes are poorly understood and rarely represented in marine and freshwater interactions with the atmosphere in numerical models^6,13,14^.

Although the hydrodynamic energy of rivers and creeks is generally higher than that of marine systems, several mechanisms can still sustain a discrete microlayer at the air–water interface. In marine systems, the accumulation of surfactants, exopolymeric substances, and surface-active organic matter can dampen small-scale turbulence and create surface-tension gradients that stabilise the SML even under moderate flow^15,16^. Evidence from both natural and engineered freshwater systems further indicates that distinguishable microlayers can persist in reservoirs and constructed wetland channels, where surfactant and organic matter accumulation reduce near-surface turbulence^17^. However, to the best of our knowledge, no previous studies have explicitly characterised the SML in headwater river systems or explored the potential for distinct SML to form in headwaters.

Material known to accumulate in the SML of marine, lake, and wetland waters includes a range of naturally occurring organic matter, such as carbohydrates, proteins, lipids^5^ and the key nutrients, including nitrogen and phosphorus^18^. The enrichment factor (EF), defined as the ratio of the concentration of a specific substance or property in the SML to its corresponding value in the underlying SSW, is a widely utilised metric for assessing the accumulation of substances and the distinct characteristics of the SML relative to the SSW^1,19–22^. The primary sources of material to the SML are phytoplankton exudates, transported to the surface via rising bubbles and diffusion^23^. Additionally, hydrophilic polysaccharides, high molecular polysaccharides and complex glucans, major excretion productions of phytoplankton, can become enriched in the SML^5^. In rivers, the surfactant compositional variation between the SML and SSW has been shown in the estuary of the Delaware Bay and the Broadkill River^2^, the estuary of the Selangor River^24^ and the Tyne Estuary^25^. An investigation of the Guangzhou segment of the Pearl River further confirmed the enrichment of nutrients (nitrogen, and phosphorus), heavy metals (Fe, Mn, Ni, Cr, and Pb), bacteria, and increased chemical oxygen demand^9,11^ in the SML. However, to our best knowledge, the organic matter SML properties in headwater tropical rainforest rivers are unknown.

The characteristics of dissolved organic matter (DOM) in aquatic environments vary depending on its source (terrestrial or aquatic) and diagenetic state^26^. Freshwater, estuarine, and marine ecosystems receive large quantities of dissolved organic carbon (DOC) from terrestrial ecosystems, altering their physical and chemical qualities and affecting their metabolic functioning^27^. Instream DOC mineralisation is a notable source of riverine carbon dioxide (CO_2_) emission, particularly in tropical rivers [e.g^28,29^. with compositional changes of DOM widely observed using a variety of approaches, including coloured and fluorescence DOM (CDOM and FDOM) techniques [e.g^30,31^, liquid chromatography organic carbon and nitrogen detection^22,32–34^, high-resolution mass spectrometry^6,35^, and nuclear magnetic resonance^36,37^.

Tropical rivers are a major component of the global biogeochemical cycling^38^, with headwaters estimated to account for 70–80% of the total river network^39^ and connect the terrestrial and aquatic ecosystems as they are in direct contact with adjacent soils^40,41^. The transition between dry and wet periods in these ecosystems can affect the water and atmospheric composition^42,43^. Despite their importance, little is known about the temporal dynamics of DOM. Given the relatively high organic matter fluxes of these systems^44^, this study questioned whether partitioning between SML and SSW exists in tropical headwaters. Therefore, this study aims to characterise temporal variations in DOC and DOM composition during the dry-to-wet season transition of the SML and SSW in a tropical rainforest headwater catchment within the Iwokrama Forest, Guyana. Specifically, we:

- Assess the temporal variability of DOC and DOM compound groups during the dry-to-wet season transition to determine how hydrological and biogeochemical changes (e.g. hydrological mobilisation, microbial or photochemical transformation) influence DOM composition and molecular characteristics.
- Examine the relationships between DOC/DOM metrics, rainfall, and river discharge to identify how hydrological forcing and rainfall events regulate DOM sources, transport pathways, and compositional dynamics in tropical headwaters.
- Quantify the differences in DOC concentration and DOM composition between the SML and SSW to evaluate enrichment processes and compositional differentiation at the air-water interface.

Through this approach, we explore how tropical headwater systems regulate carbon dynamics at the air–water interface and influence aquatic carbon cycling.

## Methodology

### Study area

**Fig. 1:**
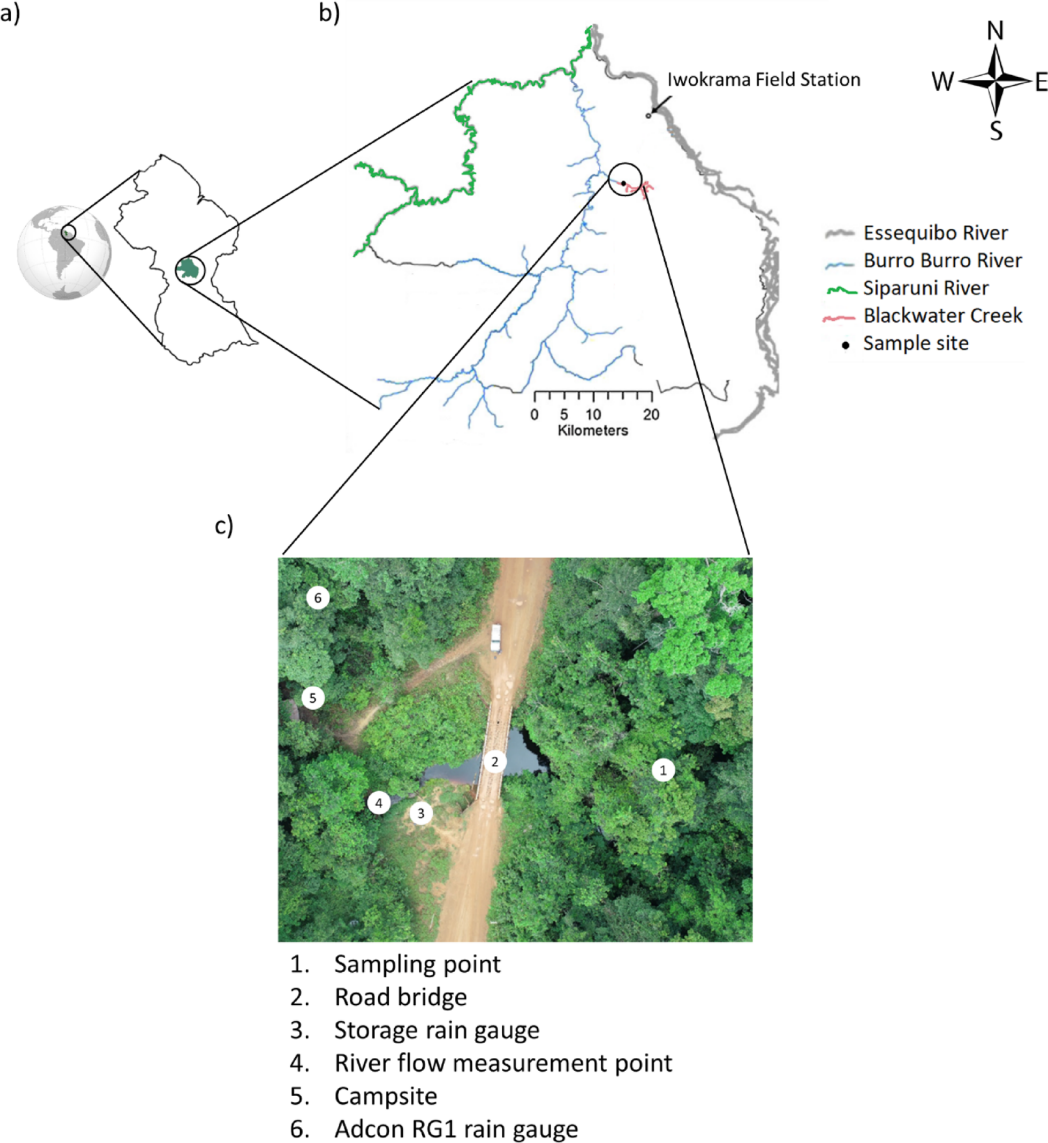
Study site map. The map of (a) Guyana, (b) the Iwokrama rainforest. (Modified from^45^, and (c) aerial view of Blackwater Creek study site (modified after^43^)

This study selected a previously characterised headwater of the Essequibo River located in the Iwokrama rainforest (Fig. 1) in the deep rainforest interior of Guyana, South America^43–45.^ The Iwokrama forest is in the heart of the Guiana Shield part of northern Amazonia, in tropical South America. The forest is bounded and drained by the Essequibo River to the East and the Siparuni River, a tributary of the Essequibo, to the west and north (See Pereira et al., 2014a for more details). Briefly, Iwokrama is located in an isolated intact rainforest near a transition in the climate regime from the north (coastal) to south (savannah). The coastal region experiences two wet seasons (a primary wet season from May to July and a secondary wet season from December to January) and two dry seasons (a primary dry season centred around October and a secondary dry season around March). In contrast, the southern savannah region has a single wet season from May to August and a long dry season extending from September to March^45,46^. Blackwater Creek (BC), a second-order headwater of the Essequibo River, was chosen to study the temporal variability of riverine DOM concentration and composition in the SML and SSW to further our understanding on dynamic headwater river systems.

### Hydrometeorological data collection

Rainfall was measured at the site using an Adcon RG1 rain gauge equipped with a 0.2 mm tipping bucket with a 200 cm^2^ diameter from 3^rd^ to 28^th^ May 2019 (no data was recorded in the last 10 days of the study period due to the rain gauge failure) and a storage rain gauge with a 127 mm diameter, placed on the ground was used to record daily rainfall from 14^th^ May to 7^th^ June 2019^45^.

River stage was measured using sealed Solinst Levelogger Edge pressure transducers located in a stilling well recording every 1 min from 3^rd^ May to 7^th^ June 2019. River stage was corrected for atmospheric pressure changes using a Solinst Barologger. River discharge for the whole study period was calculated from an empirical relationship between the measured discharge and river stage (rating curve), as follows:

1 $$\:Q=2.309\:{(S-0.57)}^{1.575}$$

where *Q* is river discharge (m^3^ s^− 1^) and *S* is the river stage (m). (For further details and complete data set used for rating curves please refer to^43^.)

### Sample Collection, preservation and analysis

SSW grab samples were collected^47^ into 60 ml high density polyethylene (HDPE) bottles from the top 50 cm of the river water column from 4^th^ May until 7^th^ June 2019 during four intensive sampling phases from 4^th^ May 12:00 to 10^th^ May 05:00 (First Phase), 13^th^ May 14:00 to 18^th^ May 05:00 (Second Phase), 21^st^ May 11:00 to 28^th^ May 05:00 (Third Phase) and 31^st^ May 10:00 to 7^th^ June 05:00 (Fourth Phase) 2019. SML samples were collected using a Garrett Screen^48^ at the same time as SSW samples. The screen (mesh 16, 0.36 mm wire diameter with an opening of 1.25 mm, a frame width of 1.25 cm) had an effective surface area of 101 cm^2^. A 1.5 m stainless steel rod was attached to the frame to minimise disturbing the SML. The screen was gently placed on the water’s surface allowing it to capture the SML and then withdrawn slowly and drained directly into 60 ml HDPE bottles^49^. These steps were repeated until two sample bottles were filled (3.5 dips per bottle on average). Samples were collected every 6 h at 05:00, 11:00, 17:00 and 23:00 (baseline samples) to capture diurnal variation and extra water samples were collected every 30 min during rainstorm events in response to observed hydrological changes such as rainfall and/or increased river discharge (rainstorm event samples). In total, 238 SML samples (94 baseline and 144 rainstorm event samples) were collected from BC. The sampling team recorded whether it was raining at the time of each sample collection, categorising samples into rain event samples (48 samples) collected during rainfall, and dry condition samples (190 samples) collected under non-rainy conditions. The identification of rain event samples was then cross-checked against rainfall data from the Adcon RG1 rain gauge, using a threshold of > 5 mL min⁻¹.

All water samples were collected following standard protocols outlined by previous work for Liquid chromatography-organic carbon detection-organic nitrogen detection (LC-OCD-OND) determination^50,51^. Briefly, water samples were filtered immediately using pre-combusted GF-F filters (450 °C for 8 h) collected in 60 ml HDPE bottles which were acid-washed (10% HCl) and rinsed with deionised water (18.2 M Ω cm^− 1^, carbon-free) prior to use. All samples were transported to a mobile laboratory in the Iwokrama River Lodge under dark and cold conditions using a portable chiller (4–6 °C).

### Field analysis and mobile laboratory

DOC analyses were completed on the same day as sample collection using a Sievers M5310C Portable TOC Analyzer, with attached GE Autosampler with a 0.03 to 50 mg L^− 1^ carbon range in the mobile laboratory at the Iwokrama River Lodge. DOC results were within the specification of < 1% RSD precision and ± 2% accuracy. Deionised water was used as an analytical blank.

DOC EFs were calculated as follows:

2 $$\:\:DOC\:EF=\frac{{DOC}_{SML}}{{DOC}_{SSW}}$$

where $\:{DOC}_{SML}$ (mg L^− 1^) is the concentration of the substance or property in the SML and $\:DO{C}_{SSW}$ (mg L^− 1^) is the concentration of the substance or property in the SSW. EF higher than 1 indicates enrichment of the substance or property in the SML relative to the SSW. EF less than 1 indicates enrichment of the substance or property in the SSW relative to the SML.

### Liquid chromatography-organic carbon detection-organic nitrogen detection (LC-OCD-OND)

A second set of 60 ml HDPE bottles were sealed at the field sites, and then kept at −20 °C until analysed at the Lyell Centre at Heriot-Watt University, UK, for further DOM compositional analysis^34,51,52^.

The DOM composition was measured using LC-OCD-OND for SML and SSW water samples. LC-OCD-OND is a size exclusion chromatography (SEC) technique, providing quantitative information regarding organic carbon compound groups^53^. LC-OCD-OND allows 1 mL of whole water to be injected onto a size exclusion column (2 mL min^− 1^; HW50S, Tosoh, Japan) with a phosphate buffer (potassium dihydrogen phosphate 1.2 g L^− 1^ plus 2 g L^− 1^ di-sodium hydrogen phosphate dihydrate, pH 6.58) and separated into five DOM compound groups. These include biopolymers (BP; high molecular weight polysaccharides and proteins), humic substances (HS), building blocks (BB; lower molecular weight HS), low molecular weight acids (LMWA), and low molecular weight neutrals (LMWN; amphiphilic/neutral compounds including alcohols, aldehydes, ketones, and amino acids)^32,53^.

LC-OCD-OND can be used to determine the nominal molecular weight of HS (M_n_) in natural waters^53^, which can describe the apparent size or weight of the HS relative to the known molecular weight of International Humic Substances Society (IHSS) humic and fulvic acid (HA and FA, respectively). This technique has been used in the study of organic matter in SML and SSW of freshwater or marine systems^44,54,55^. The resulting compound groups were identified using detectors for organic carbon (OC), UV-amenable carbon and organic nitrogen (ON)^53^. All peaks were identified and quantified with bespoke software (Labview, 2013) normalized to IHSS HA and FA standards, potassium hydrogen phthalate and potassium nitrate.

EFs were calculated for BP, HS, BB, LMWN, LMWA concentration and M_n_ following Eq. (1). These were denoted as BP EF, HS EF, BB EF, LMWN EF, LMWA EF, and M_n_ EF, respectively.

### Statistical analysis

All statistical analyses were performed using R software (version 4.3.3) and Microsoft Excel (version 365). Prior to analysis, Rosner’s test for outliers was applied to identify and remove extreme values, ensuring the robustness of the results. Group differences were assessed using independent samples *t*-tests assuming unequal variances, with the *t*-statistic used to determine statistical significance at a threshold of α = 0.05. Specifically, *t*-tests were used to compare (i) DOC concentrations between SML and SSW, (ii) DOC concentrations and DOM EFs between rain event and dry-period samples, and (iii) DOM compound group EFs (BP, HS, BB, LMWN, and LMWA) between rising and falling river discharge conditions.

To evaluate relationships between variables, linear regression analyses were conducted. These included (i) relationships between HS and DOC concentrations for SML and SSW during the drier (phases 1–2) and wetter (phases 3–4) periods, and (ii) relationships between DOC and other DOM compound groups (BP, BB, LMWN, and LMWA). The slopes and intercepts of regression models were compared using analysis of covariance (ANCOVA) to determine statistically significant differences between groups (e.g. between SML and SSW, and between dry and wet transition periods). The coefficient of variation (CV) was calculated to assess relative variability within each data set and across sampling phases.

Temporal trends in DOC concentration, HS M_n_, and DOM composition were evaluated using the non-parametric Mann–Kendall test, which detects monotonic trends in time-series data without assuming normality. A significance threshold of α = 0.05 was applied to all tests.

## Results

### Medium-term climate and hydrology

**Fig. 2:**
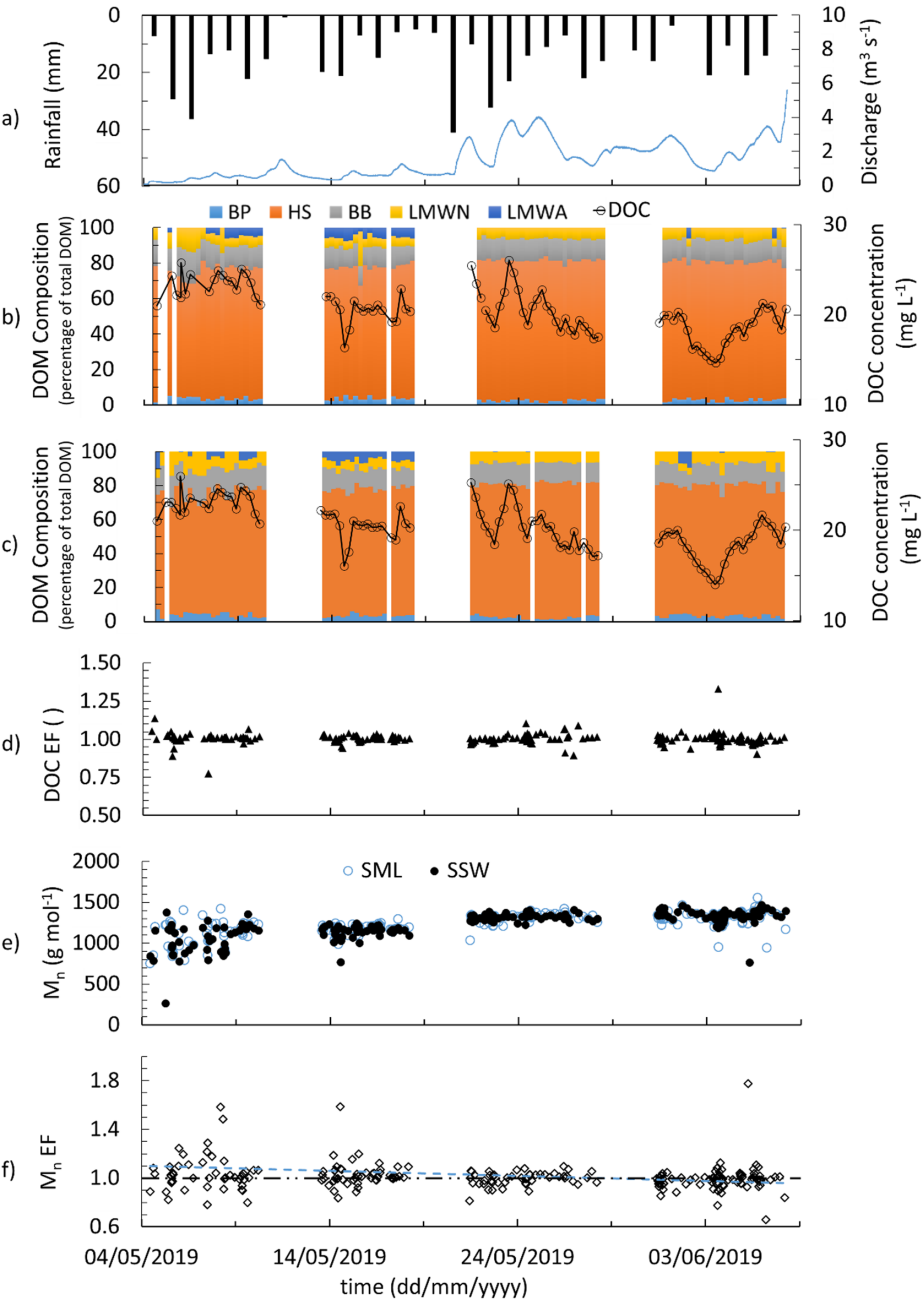
BC daily rainfall (black bar) and river discharge (blue line) (**a**), temporal variability of the percentage of the total DOM pool and DOC concentration in baseline SML (**b**) and SSW samples (**c**), DOC EF (**d**), M_n_ of HS in the SML and SSW (e), and the HS M_n_ EF (**f**). The blue dashed line shows the general trend of M_n_ EF and the black dashed line shows EF = 1 in (**f**).

The total monthly rainfall in the study site for May 2019 was 421 mm. The river discharge ranged from 0.05 to 5.59 m^3^ s^− 1^ and the river water level ranged from 0.66 m to 2.32 m (SD = 0.34) (Figs. 2a and 4a). The distinction between wet and dry periods in this study follows the climatic characterisation reported elsewhere^43^. Briefly, monthly rainfall data recorded at the Iwokrama Field Station from January 2016 to December 2019 show a bimodal precipitation pattern with two wet and two dry seasons typical of the Guianas region^46^. Based on the Köppen-Geiger system for the Guianas (rainfall in the driest month is ≥ 60 mm or < [100 mm – (mean annual rainfall/25)]), 2019 started with a wet season that began in April 2018^56^.

### Temporal dynamics of DOC and DOM composition

#### Temporal variability of DOC concentration

DOC concentrations ranged from 9.33 to 26.41 mg L^− 1^ (20.01 ± 2.92 mg L^− 1^) for the SSW (Fig. 2c) and from 9.82 to 26.08 mg L^− 1^ (20.19 ± 2.89 mg L^− 1^) for the SML (Fig. 2b) over the study period. The DOC concentration during rainstorm event SML samples (*n* = 144, ranging from 13.87 to 25.28 mg L^− 1^, 20.02 ± 2.91 mg L^− 1^) does not deviate from baseline samples (*n* = 94, ranging from 9.82 to 26.08 mg L^− 1^, 20.36 ± 1.81 mg L^− 1^) (t-test: two-sample assuming unequal variances, t static =−1.08, t critical = 1.66). The enrichment of SML DOC concentration relative to the SSW, DOC EF ranged from 0.78 to 1.33 (1.00 ± 0.04) over the study period (Fig. 2d). The majority of samples (96% of the total sample set) reported a DOC EF between 0.92 and 1.08 (1 ± 2 × SD (= 0.04)). As both SML and SSW DOC were normally distributed, t-tests suggest that the SML DOC and SSW DOC are not statistically different (t static = 0.36, t critical = 1.97). The Mann–Kendall test further indicated a downward temporal trend in DOC concentration for both the SML and SSW (z = − 2.48 and − 2.75, respectively; *p* < 0.05) (Table S9), suggesting a gradual decrease over the study period.

#### Temporal variability of DOM compound groups

The BP concentration ranged from 0.06 to 1.44 mg L^− 1^ (0.56 ± 0.30 mg L^− 1^) in SML and from 0.02 to 1.44 mg L^− 1^ (0.56 ± 0.29 mg L^− 1^) in the SSW. BP accounted for 2.8 ± 1.1% of the total DOM present in the SML (0.6 to 5.8%) (Fig. 2b) and 2.91 ± 1.1% of the DOM in the SSW (0.5 to 6.7%) (Fig. 2c). HS, which is the dominant compound group observed, ranged from 3.25 to 20.69 mg L^− 1^ (14.30 ± 3.50 mg L^− 1^) in the SML and from 0.27 to 21.66 mg L^− 1^ (14.08 ± 3.99 mg L^− 1^) in the SSW. Generally, the HS fraction increases in both SML and SSW waters ranging from 54.6 to 89.3% in the SML (73.7 ± 6.3%; Fig. 2b) and 54.9 to 84.2% in the SSW during the study period (73.5 ± 6.0%; Fig. 2c). The BB concentrations ranged from 0.51 to 4.54 mg l^− 1^ (2.34 ± 0.67 mg L^− 1^) in the SML and from 0.07 to 4.50 mg L^− 1^ (2.31 ± 0.76 mg L^− 1^) in the SSW. The BB accounted for 9.1 to 18.5% (12.4 ± 1.7%) of DOM in the SML (Fig. 2b) and 7.2 to 18.7% (12.1 ± 1.9%) in the SSW (Fig. 2c). The LMWNs were found at concentrations of 0.08 to 5.09 mg L^− 1^ (1.30 ± 0.63 mg L^− 1^) in the SML and 0.01 to 3.82 mg L^− 1^ (1.29 ± 0.65 mg L^− 1^) in the SSW. The LMWNs contributed 0.0 to 22.0% (6.6 ± 2.8%) to the total DOM in the SML (Fig. 2b) and 0.0 to 26.1% (6.8 ± 3.1%) in the SSW (Fig. 2c). The BP, BB and LMWN overall concentrations and contributions to the total DOM pool generally decreased over the study period in both SML and SSW waters. The LMWAs concentration ranged from below detection to 1.48 mg L^− 1^ (0.92 ± 0.45 mg L^− 1^) and 1.59 mg L^− 1^ (0.80 ± 0.47 mg L^− 1^) in the SML and SSW, respectively. When present, LMWAs contributed 0.4 to 7.5% (4.2 ± 2.2%) to DOM in the SML (Fig. 2b) and 7.2 to 10.2% (4.9 ± 1.8%) in the SSW (Fig. 2c).

The LMWAs were detected inconsistently during the first phase of the study, appearing in 22 SML and 24 SSW samples (out of 50 total sample pairs). LMWAs were found in 11 SSW samples without corresponding detection in the SML and in 9 SML samples below detection in the SSW. During the second phase, LMWAs were detected in all waters (100 samples in total). In the third and fourth phases, LMWAs were detected in both the SML and SSW for 5 sample pairs.

The Mann–Kendall trend analysis revealed statistically significant temporal patterns in several DOM compound groups (Table S8). In the SML and SSW, significant increasing trends were observed for HS percentage of total DOM (z = 4.05, *p* < 0.01 and z = 4.27, *p* < 0.01, respectively), whereas BB and LMWN percentages exhibited significant decreasing trends (BB: z = − 3.07, *p* < 0.01 in the SML; z = − 3.29, *p* < 0.01 in the SSW; LMWN: z = − 3.03, *p* < 0.01 in the SML; z = − 3.03, *p* < 0.01 in the SSW). BP showed a weak decreasing tendency in the SML (z = − 1.85, *p* = 0.06) and a significant decline in the SSW (z = − 2.82, *p* < 0.01), while LMWA showed no significant temporal change (*p* > 0.9) (Table S9).

#### Variations in HS and molecular characteristics

**Fig. 3:**
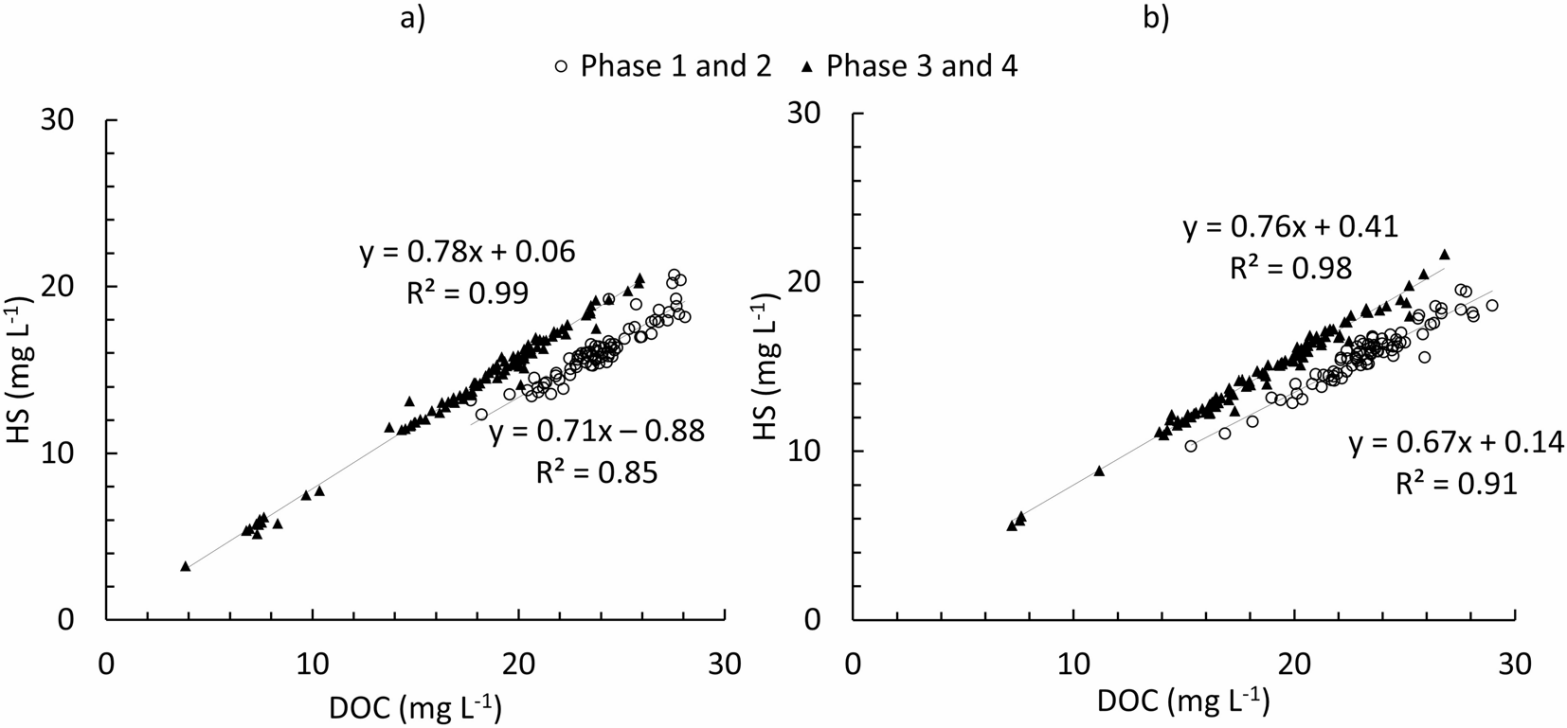
BC HS vs. DOC concentration for the SML (**a**) and SSW (**b**) in the first (circles) and second (triangles) half of the study period.

DOM data from this study were categorised into two groups: phase 1 and 2 (first half of the study period) and phase 3 and 4 (second half of the study period) based on earlier observations of DOM and river discharge by^43^. Notably, HS represents a greater proportion of the DOC in the first half of the study compared to the latter half in both the SML and the SSW (Fig. 3).

Figure 3 further shows that the relationship of HS to the total DOC pool for phases 1 and 2 (first half of the study period and drier) and phases 3 and 4 (second half of the study period and wetter) follow different trends, evidenced by two different sets of slopes and intercepts. The HS and DOC concentrations show a strong correlation in SML (R^2^ = 0.99, *n* = 103, *p* < 0.0001 in phase 1 and 2 and R^2^ = 0.85, *n* = 135, *p* < 0.0001 in phase 3 and 4) and SSW (R^2^ = 0.98 *n* = 103, *p* < 0.0001 in phase 1 and 2 and R^2^ = 0.91, *n* = 135, *p* < 0.0001 in phase 3 and 4). The comparative analysis of the linear regression slopes of HS and DOC relationship for two groups of data (group 1: phase 1 and 2, group 2: phase 3 and 4) suggests a significant but variable relationship between HS and DOC between these two groups (SML: *p* < 0.0001, α = 0.05, SSW: *p* < 0.0001, α = 0.05) despite the intercept not being significantly different in these two groups (SML: *p* = 0.31, SSW: *p* = 0.23). In addition, the interaction term (i.e. the statistical test of whether the slope of the HS-DOC relationship differs significantly between SML and SSW) suggests statistically significant differences in the second half of the study period (*p* = 0.004, α = 0.05). However, no statistically significant difference was observed between the intercepts (*p* = 0.21, α = 0.05) and no statistically significant difference was observed between the intercept and interaction term in the first half of the study period (intercept: *p* = 0.84, α = 0.05, interaction term *p* = 0.48, α = 0.05). In addition, the linear relationship between DOC and other DOM compound groups (BP, BB, LMWN, and LMWA) was tested. For all groups, the correlations were weak (R^2^ < 0.8) except for BB in the first half of the study period (R² = 0.86 for SML and R² = 0.95 for SSW).

HS M_n_ increases over the study period (ranges from 747 to 1553 (1236 ± 134) g mol^− 1^ in the SSW and from 763 to 1465 (1232 ± 151) g mol^− 1^ in the SML (Fig. 2e). In the first phase of the study period, HS M_n_ has a high SML and SSW variability (CV = 15 and 15% for the first phase, respectively), which decreases during the study period (for example, CV = 7 and 7% for the fourth phase, respectively). The M_n_ EF ranges from 0.66 to 1.70 (1.01 ± 0.11) with maximum variability in the first phase (CV = 14%, second phase CV = 10%, third phase CV = 5% and fourth phase CV = 11%) (Fig. 2f). The comparison of humic substances diagram (HS-diagram, the SAC/OC ratio (aromaticity) of aquatic humic substances plotted against M_n_)^53^ in different phases shows a gradual shift of the samples to higher molecularity and lower aromaticity from phase 1 to phase 4 (SI section H, Figure S28 to 32).

### SML–SSW compositional differences and enrichment factors

**Fig. 4:**
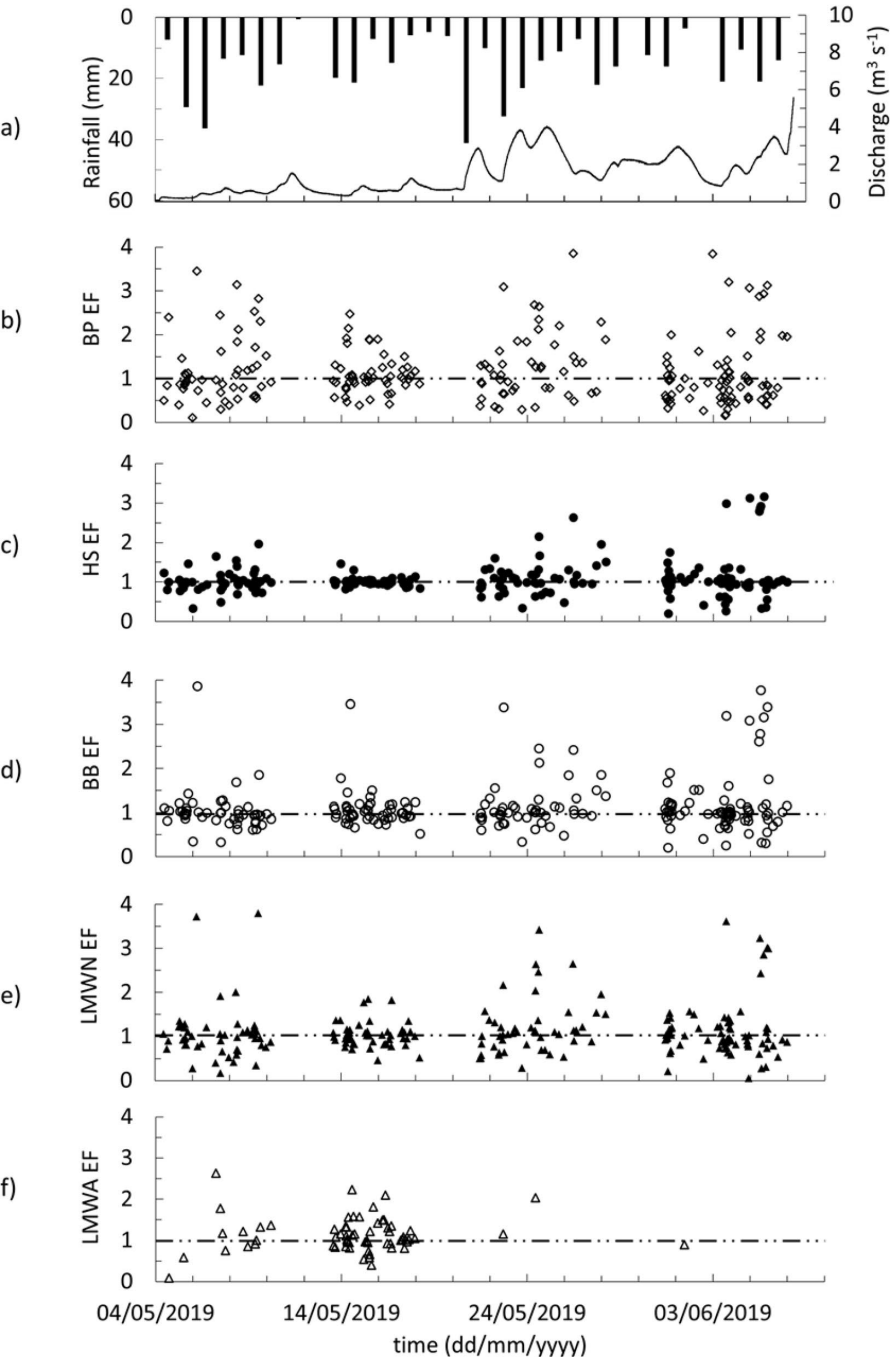
BC daily rainfall (black bar) and river discharge (line) (**a**) and the temporal variability of EF of DOM compound groups (**b**) to (**f**) (dashed lines mark EF = 1).

The EF of the DOM compound groups is used for the compositional comparison of the SML and SSW. The BP EF ranges from 0.41 to 8.03 (1.24 ± 0.99) (Fig. 4b), HS EF ranges from 0.21 to 7.38 (1.16 ± 0.76) (Fig. 4c), BB EF ranges from 0.21 to 7.61 (1.16 ± 0.75) (Fig. 4d), LMWN fraction EF ranges from 0.05 to 5.97(1.19 ± 0.78) (Fig. 4e) and LMWA fraction EF ranges from 0.09 to 2.63 (1.14 ± 0.43) (Fig. 4f). BP EF shows the maximum variability (CV = 80%) among DOM compound groups (HS EF (CV = 66%), BB EF (CV = 64%), LMWN EF (CV = 66%), and LMWA (CV = 38%)). BP, HS, BB, and LMWN EFs are more variable in the second half of the study period (CV = 55, 48, 42, and 58% in phase 1 and 2 and CV = 91, 74, 73, and 70% in phase 3 and 4, respectively). Weak linear relationships were observed between the EF of all DOM compound groups (R²<0.7, *n* = 238, *p* < 0.0001), except for BB and HS, which showed a strong correlation (R²=0.92, *n* = 238, *p* < 0.0001).

As such, the DOM EFs of these regimes were investigated to assess whether the rising or falling phases exert control over the compositional differences between the SML and SSW. Two categories of water samples collected during rising and falling river discharge in the second half of the study period (as it has distinct rising and falling periods) were statistically compared. No statistically significant variation of DOM composition was observed in relation to river discharge trends (t critical = 2.00 and t static = 0.83, 0.82, 0.68, and 0.55 and 1.51 for BP EF, HS EF, BB EF, LMWN EF, and LMWA EF, respectively).

To evaluate the relationship between rain events and compositional variations in the water column, rain event samples and dry condition samples were compared. Given the normal distribution of the data and the unequal variance between the groups, t-tests were performed. In all cases, the calculated t-statistics were lower than the corresponding critical t-values, indicating no statistically significant compositional variation in the water column between the samples collected during rain events and those collected during dry periods (t critical = 1.98 and t static = 1.11, 0.94, 1.14, and 1.32 for BP EF, HS EF, BB EF, and LMWN, respectively). To determine whether high EF values (spikes) are associated with rain events, EF values greater than two were filtered, and the proportion of samples with high EF values collected during rain events was compared to the total number of such samples. The percentages of high EF samples collected during rain events were 21% (6 out of 29 samples) for BP EF, 29% (4 out of 14 samples) for HS EF, 27% (4 out of 15 samples) for BB EF, 20% (4 out of 20 samples) for LMWN EF, and 62% (5 out of 8 samples) for LMWA EF. Since 26% of all samples were collected during rain events, these percentages are generally close to or below the overall sampling proportion, except for LMWA. This distribution indicates that high EF values occurred in both rain and dry conditions at similar frequencies, suggesting no consistent or systematic increase in EF during rain events. Therefore, the data do not support a strong association between rain conditions and the occurrence of high EF values.

## Discussion

### Seasonal hydrological connectivity and implications for DOM transport

The dry season typically occurs from February to April, and the wet season from May to August, with transitional periods in between. The present study was conducted in May 2019, during the onset of the major wet season, corresponding to the dry-to-wet transitional period^43^. To briefly place the study period in a broader context, monthly precipitation totals for the month of May at the Iwokrama Field Station from 2016 to 2019 were 283, 425, 898, and 421 mm, respectively^43^. The 2019 value therefore represents a typical early-wet-season rainfall for the region, neither unusually high nor low relative to interannual variability. This suggests that the catchment was transitioning from dry to wet season during the 35-day sampling period. The Iwokrama headwater catchment exhibits a clear and rapid hydrological response to rainfall^43^, which our study period captures. At the onset of the wet season, increasing rainfall likely mobilises shallow groundwater and near-surface flow paths, producing short-lived discharge peaks as the catchment becomes progressively hydraulically connected. During drier periods, river flow is likely sustained primarily by groundwater inputs, resulting in lower and more stable base flow. This seasonal alternation between surface-dominated and groundwater-dominated conditions strongly influences DOC–discharge relationships and the type of organic matter exported from the catchment^57^.

### DOC and DOM through the dry to wet season transition

In the transition from the dry to wet season, we observed SSW DOC concentrations at the lower range of DOC values previously reported for this river that captured intense rain events^44^. However, here we observe that during the first phase of our study period, where drier conditions prevail, DOC concentrations are generally higher. As river discharge increases, reflecting wet season conditions, DOC is less concentrated albeit with a greater overall flux that is responsive to fluctuations in river discharge^43^. Interestingly, when the river discharge is at its lowest in the dry season the variability in the composition of DOM in the SSW is at its greatest. At this time, LC-OCD-OND analysis demonstrates that DOM exhibits a lower presence of HS and a higher abundance of BP, BB, LMWN, and LMWA. However, as more water enters the catchment, the range in DOC concentration increases, with very little variability in DOM composition, a notable absence of LMWA, and a greater proportion of HS.

The high variability of DOM composition during lower discharge indicates contributions from a more heterogeneous mix of organic matter sources within the catchment. This compositional variability is particularly evident in the HS fraction, with M_n_ showing higher variability early in the study period before declining over time and a shift in the position of the samples in HS-diagram. As the catchment becomes wetter with higher river discharge it is likely that DOM sources become homogenised^43^. That earlier study demonstrated that during the onset of the wet season, DOC concentrations can decline even as total DOM flux increases, due to dilution and the mobilisation of more distal or humic-rich sources. Consistent with this, the observed decline in M_n_ variability supports the hydrological mobilisation of dry-season DOM sources by rainfall, followed by the increasing influence of a more dominant source, likely high-molecular-weight HS delivered via wet-season dynamics. However, DOM and in particular HS can absorb solar radiation, leading to significant reduction in abundance, and changes in composition and molecular size^58,59^. Photochemical reactions mainly occur in the photic zone of the upper water column and have been observed to increase the molecular weight of DOM^60^, while microbial degradation is ubiquitous and can consume up to 60% of DOM^61^. While HS is traditionally considered chemically stable and resistant to microbial degradation^62,^ there is evidence that bacteria can utilise DOM as a substrate^63^. The observed changes in HS molecular weight during the study are therefore consistent with concurrent photochemical activity and microbial reworking processes acting on DOM within the water column.

### SML and SSW compositional differences

We observe that turbulent river flow homogenises the water column in terms of DOC concentration between the SML and the SSW (DOC EF CV = 10%). The observation that these compound groups are not uniformly distributed between SML and SSW likely indicate a change in either the hydrodynamic regime of the river system, a change in the supply of organic matter to the river, compositional variation of the compound groups, or a change in the degradation pathway or rate of organic matter in the river.

The high temporal variability of EFs for all DOM compound groups demonstrates the dynamic nature of a headwater tropical river. The changing pattern of DOM compound enrichments in the SML throughout our time series is notable. The variability of EFs for BP, HS, BB, and LMWN is greater in the wet transition period with BP and LMWN compounds more frequently observed to fluctuate between depleted to enriched in the SML (Fig. 4b to e).

The weak EF relationships observed for LMWAs, LMWNs, and BPs suggests that these compound groups migrate independently between the SML and SSW. This may suggest that they are derived from different sources or are subjected to differing transformation processes. It may also suggest that the chemical composition of these compound groups have notable differences in their relative buoyancy. Intriguingly, the observed relationship with HS and BB EFs suggests these DOM compound groups may be coupled together when transported in either the SML or SSW.

The significantly different slope and intercept of the relationship between HS and DOC in the SML and SSW, indicates compositional differences of HS further supported by M_n_ dynamics. The EFs demonstrate a higher variability early in the study period indicating preferential accumulation of high-molecular-weight HS in the SML at this time. Towards the wet-season phase, HS variability decreases coincident with more uniform SML-SSW composition. Combined, this may suggest that the supply, processing and/or migration of HS between the SML and SSW are in a quasi-steady state.

Our results show no immediate or consistent association between individual rain events and high EF values, indicating that direct rainfall does not lead to an EF pulse. Thus, the observed EF pulses may result from complex, rainfall-initiated processes or instream autochthonous processes rather allochthonous supply or direct rain effects. This is consistent with the idea that discrete and frequent rain events, such as those associated with tropical rainforests, deliver organic substances with variable buoyancy or surface activity potential to the river, as proposed for the Delaware basin^2^. While differentiating individual mechanisms of SML enrichments between organic matter supply and degradation is challenging due to intertwined and competing processes, the rising trend of higher molecular weight humic substances combined with a reduction in SML enrichment over our study period does suggest a change in source composition. Whether this is reflective of the other components of the DOM pool is unknown. The absence of LMWA in the second half of study may suggest reactions with humic substances in the SML generating volatile organic compounds^64^. The stabilisation of HS EFs in the second phase, when LMWA was consistently present, further highlights the linkage between LMWA and HS in driving SML-SSW compositional differences. Alternatively, the increasing variability in the EFs of BP and LMWN coupled with the notable absence of LMWA in the wet transition period may indicate an increased microbial presence and activity in the SML^9,49,65^. Assuming that the compounds are predominantly derived from microbial sources, the absence of acids may be a result of higher demand than supply for this compound group as observed in microbial incubation systems^66^.

## Implications

In the paucity of freshwater studies, we note that marine systems have been shown to have SML enrichments of DOM in upwelling regions likely derived from autochthonous microbial release of DOM as a response to light exposure in this environment^67^. High-resolution observations of DOM in the coastal and open ocean have demonstareted that increasing wind speeds, solar radiation and photochemical degradation lower SML enrichments of organic material^20,25^. However, organic enrichments within the SML can persist under high turbulence conditions^68^ with bubbles in particular enriching the microlayer further^2^. Additionally, the presence of surfactants and humic substances in the SML may attenuate turbulence^69^ with reductions in the exchange of gases including oxygen^70^ and CO_2_^3,12^. Importantly, the variable suppression of gas exchange at similar concentrations of surfactants in the SML suggests organic composition has a notable impact. However, whether these mechanisms are directly comparable to those of headwater river systems remains unknown^2,67,68^.

Our study reveals compositional variation in the water column, even when DOC concentrations remain similar. The interplay between these compositional DOM changes demonstrates that rivers are never truly well-mixed with the surface microlayer as a distinct chemical layer even in headwater river systems. The dynamic nature of the SML at the water-atmosphere interface likely contributes to highly variable gas exchange rates, similar to observations in marine environments^3,12^. However, the uncertainty surrounding riverine greenhouse gas outgassing highlights the need for research across various scales of river catchments. In particular, mechanisms driving water column mixing in small headwaters may differ fundamentally from those in larger downstream systems. Expanding such studies in tropical rivers and other aquatic systems will enhance our understanding of the SML’s role in global biogeochemical cycles.

## Supplementary Information

Below is the link to the electronic supplementary material.

Supplementary Material 1

## Data Availability

The data utilised in this research study is available as supplemental material documents within this manuscript. Furthermore, these data are deposited in Mendeley repository ([here](https:/data.mendeley.com/preview/9fx2dvnk7t? a=53396f18-ac88-497d-8131-4e929faf0bc2)) with the title of this manuscript and will be published upon acceptance of this manuscript for publication. For any further queries regarding the dataset, please contact the corresponding authors.
