# Supplementary material for: Temporal changes of headwater river surface microlayer characteristics during the dry to wet season transition in the tropical rainforest of Guyana
Source: Sci Rep. 2025 Nov 28;15:45568. doi: 10.1038/s41598-025-28843-4 (PMC12753671; doi:10.1038/s41598-025-28843-4)
Supplement: Supplementary file 1 — Supplementary Material 1 [file 41598_2025_28843_MOESM1_ESM.docx]

# **Supplementary Information**

# **A: Rainfall data**

**Table S1:** Blackwater Creek (BC) rainfall data

| Date | Rain (mm) (Storage Rain Guage) | Rain (mm) (Adcon RG1 Rain Gauge) |
| --- | --- | --- |
| 04/05/2019 |  | 7.25 |
| 05/05/2019 |  | 29.25 |
| 06/05/2019 |  | 36.25 |
| 07/05/2019 |  | 13.50 |
| 08/05/2019 |  | 12.25 |
| 09/05/2019 |  | 22.25 |
| 10/05/2019 |  | 15.25 |
| 11/05/2019 |  | 0.50 |
| 12/05/2019 |  | 0.00 |
| 13/05/2019 |  | 19.75 |
| 14/05/2019 |  | 21.25 |
| 15/05/2019 |  | 7.00 |
| 16/05/2019 |  | 14.75 |
| 17/05/2019 |  | 5.75 |
| 18/05/2019 |  | 4.75 |
| 19/05/2019 |  | 6.00 |
| 20/05/2019 |  | 41.00 |
| 21/05/2019 |  | 10.00 |
| 22/05/2019 |  | 32.25 |
| 23/05/2019 |  | 23.00 |
| 24/05/2019 |  | 14.00 |
| 25/05/2019 |  | 11.00 |
| 26/05/2019 |  | 7.00 |
| 27/05/2019 |  | 22.00 |
| 28/05/2019 |  | 16.00 |
| 29/05/2019 | 0.50 |  |
| 30/05/2019 | 12.25 |  |
| 31/05/2019 | 16.00 |  |
| 01/06/2019 | 3.48 |  |
| 02/06/2019 | 0.00 |  |
| 03/06/2019 | 20.91 |  |
| 04/06/2019 | 10.45 |  |
| 05/06/2019 | 20.91 |  |
| 06/06/2019 | 13.94 |  |

# **B: TOC analyser data**

**Table S2:** BC surface microlayer (SML) dissolved organic carbon (DOC) concentration measured by TOC analyser

| Date and Time | DOC (mg/L) |
| --- | --- |
| 04/05/2019 11:00 | 9.82 |
| 04/05/2019 15:00 | 16.62 |
| 04/05/2019 17:00 | 21.02 |
| 05/05/2019 06:30 | 24.04 |
| 05/05/2019 07:30 | 23.94 |
| 05/05/2019 10:00 | 23.51 |
| 05/05/2019 12:00 | 24.31 |
| 05/05/2019 13:35 | 21.81 |
| 05/05/2019 13:59 | 23.31 |
| 05/05/2019 14:25 | 23.01 |
| 05/05/2019 14:55 | 22.91 |
| 05/05/2019 15:25 | 21.71 |
| 05/05/2019 15:55 | 22.91 |
| 05/05/2019 16:25 | 22.41 |
| 05/05/2019 18:00 | 22.21 |
| 05/05/2019 23:20 | 21.91 |
| 06/05/2019 00:00 | 25.80 |
| 06/05/2019 05:25 | 22.31 |
| 06/05/2019 07:00 | 22.70 |
| 06/05/2019 12:00 | 24.50 |
| 06/05/2019 18:00 | 25.70 |
| 07/05/2019 06:00 | 23.10 |
| 07/05/2019 11:30 | 20.51 |
| 07/05/2019 12:00 | 22.60 |
| 07/05/2019 12:00 | 23.11 |
| 07/05/2019 12:30 | 23.41 |
| 07/05/2019 14:30 | 22.81 |
| 07/05/2019 18:00 | 23.91 |
| 07/05/2019 23:00 | 24.91 |
| 08/05/2019 05:00 | 24.41 |
| 08/05/2019 08:00 | 24.41 |
| 08/05/2019 09:00 | 24.11 |
| 08/05/2019 09:30 | 23.81 |
| 08/05/2019 10:00 | 23.91 |
| 08/05/2019 10:30 | 23.71 |
| 08/05/2019 11:00 | 23.81 |
| 08/05/2019 17:00 | 23.71 |
| 08/05/2019 23:00 | 22.78 |
| 09/05/2019 05:00 | 25.08 |
| 09/05/2019 07:00 | 25.08 |
| 09/05/2019 08:00 | 25.28 |
| 09/05/2019 08:30 | 24.28 |
| 09/05/2019 09:00 | 24.88 |
| 09/05/2019 10:00 | 24.78 |
| 09/05/2019 11:00 | 24.58 |
| 09/05/2019 13:00 | 23.98 |
| 09/05/2019 15:10 | 25.08 |
| 09/05/2019 17:00 | 23.58 |
| 09/05/2019 23:00 | 21.92 |
| 10/05/2019 05:00 | 21.12 |
| 13/05/2019 14:00 | 22.33 |
| 13/05/2019 15:00 | 22.33 |
| 13/05/2019 16:00 | 22.53 |
| 13/05/2019 17:00 | 22.03 |
| 13/05/2019 23:00 | 22.03 |
| 14/05/2019 05:00 | 21.49 |
| 14/05/2019 05:30 | 21.39 |
| 14/05/2019 06:00 | 21.69 |
| 14/05/2019 06:30 | 21.49 |
| 14/05/2019 07:00 | 21.49 |
| 14/05/2019 07:30 | 21.39 |
| 14/05/2019 09:00 | 20.99 |
| 14/05/2019 10:00 | 20.89 |
| 14/05/2019 10:30 | 20.59 |
| 14/05/2019 11:00 | 20.59 |
| 14/05/2019 13:30 | 18.99 |
| 14/05/2019 14:00 | 17.99 |
| 14/05/2019 15:30 | 17.79 |
| 14/05/2019 17:00 | 16.34 |
| 14/05/2019 23:00 | 18.34 |
| 15/05/2019 05:00 | 21.54 |
| 15/05/2019 07:00 | 21.04 |
| 15/05/2019 09:00 | 20.74 |
| 15/05/2019 10:00 | 21.22 |
| 15/05/2019 11:00 | 20.72 |
| 15/05/2019 12:00 | 21.12 |
| 15/05/2019 12:30 | 20.82 |
| 15/05/2019 13:00 | 20.52 |
| 15/05/2019 13:30 | 20.22 |
| 15/05/2019 15:00 | 20.42 |
| 15/05/2019 17:00 | 20.42 |
| 15/05/2019 23:00 | 20.72 |
| 16/05/2019 05:00 | 20.42 |
| 16/05/2019 07:00 | 20.92 |
| 16/05/2019 09:00 | 21.09 |
| 16/05/2019 11:00 | 21.09 |
| 16/05/2019 12:00 | 20.99 |
| 16/05/2019 14:00 | 21.09 |
| 16/05/2019 15:30 | 21.09 |
| 16/05/2019 16:30 | 21.19 |
| 16/05/2019 17:00 | 20.49 |
| 17/05/2019 05:00 | 19.19 |
| 17/05/2019 07:00 | 19.69 |
| 17/05/2019 08:00 | 19.49 |
| 17/05/2019 09:30 | 18.29 |
| 17/05/2019 11:00 | 19.27 |
| 17/05/2019 13:00 | 20.99 |
| 17/05/2019 15:00 | 22.99 |
| 17/05/2019 17:00 | 22.87 |
| 17/05/2019 23:00 | 20.67 |
| 18/05/2019 05:00 | 20.39 |
| 21/05/2019 11:00 | 25.47 |
| 21/05/2019 11:50 | 24.81 |
| 21/05/2019 12:20 | 24.61 |
| 21/05/2019 12:50 | 24.61 |
| 21/05/2019 13:20 | 24.41 |
| 21/05/2019 15:00 | 23.81 |
| 21/05/2019 17:00 | 23.47 |
| 21/05/2019 23:00 | 21.87 |
| 22/05/2019 05:00 | 20.51 |
| 22/05/2019 05:50 | 20.41 |
| 22/05/2019 11:00 | 19.56 |
| 22/05/2019 11:50 | 19.56 |
| 22/05/2019 12:45 | 19.36 |
| 22/05/2019 14:00 | 19.26 |
| 22/05/2019 15:45 | 18.56 |
| 22/05/2019 16:20 | 18.32 |
| 22/05/2019 17:00 | 18.56 |
| 22/05/2019 18:05 | 17.52 |
| 22/05/2019 23:00 | 20.96 |
| 23/05/2019 05:00 | 22.52 |
| 23/05/2019 08:00 | 23.68 |
| 23/05/2019 11:00 | 26.08 |
| 23/05/2019 17:00 | 24.68 |
| 23/05/2019 23:00 | 22.78 |
| 24/05/2019 05:00 | 20.28 |
| 24/05/2019 09:00 | 18.87 |
| 24/05/2019 10:00 | 20.07 |
| 24/05/2019 10:30 | 18.37 |
| 24/05/2019 11:00 | 18.87 |
| 24/05/2019 12:00 | 18.87 |
| 24/05/2019 14:00 | 19.27 |
| 24/05/2019 14:30 | 19.77 |
| 24/05/2019 15:15 | 19.97 |
| 24/05/2019 16:00 | 19.19 |
| 24/05/2019 17:00 | 20.97 |
| 24/05/2019 18:00 | 20.19 |
| 24/05/2019 23:00 | 21.77 |
| 25/05/2019 05:00 | 22.79 |
| 25/05/2019 11:00 | 20.99 |
| 25/05/2019 17:00 | 20.45 |
| 25/05/2019 23:00 | 19.25 |
| 26/05/2019 05:00 | 18.15 |
| 26/05/2019 11:00 | 19.57 |
| 26/05/2019 11:35 | 19.57 |
| 26/05/2019 12:15 | 16.57 |
| 26/05/2019 15:05 | 18.07 |
| 26/05/2019 17:00 | 18.27 |
| 26/05/2019 23:00 | 17.77 |
| 27/05/2019 05:00 | 19.38 |
| 27/05/2019 11:00 | 18.73 |
| 27/05/2019 17:00 | 18.13 |
| 27/05/2019 23:00 | 17.33 |
| 28/05/2019 05:00 | 17.53 |
| 31/05/2019 10:00 | 18.63 |
| 31/05/2019 11:00 | 19.13 |
| 31/05/2019 12:00 | 19.23 |
| 31/05/2019 12:30 | 19.13 |
| 31/05/2019 13:00 | 19.23 |
| 31/05/2019 13:30 | 19.53 |
| 31/05/2019 14:00 | 19.63 |
| 31/05/2019 14:30 | 19.23 |
| 31/05/2019 15:00 | 19.43 |
| 31/05/2019 15:30 | 19.33 |
| 31/05/2019 16:00 | 19.53 |
| 31/05/2019 16:30 | 19.43 |
| 31/05/2019 17:00 | 19.93 |
| 31/05/2019 17:30 | 19.43 |
| 31/05/2019 18:00 | 19.53 |
| 31/05/2019 18:30 | 19.73 |
| 31/05/2019 19:00 | 18.89 |
| 31/05/2019 23:00 | 19.93 |
| 01/06/2019 05:00 | 19.39 |
| 01/06/2019 11:00 | 20.30 |
| 01/06/2019 17:00 | 19.70 |
| 01/06/2019 23:00 | 18.30 |
| 02/06/2019 05:00 | 16.20 |
| 02/06/2019 11:00 | 16.54 |
| 02/06/2019 17:00 | 15.94 |
| 02/06/2019 23:00 | 15.44 |
| 03/06/2019 05:00 | 14.94 |
| 03/06/2019 08:00 | 14.54 |
| 03/06/2019 09:00 | 14.57 |
| 03/06/2019 10:00 | 14.47 |
| 03/06/2019 11:00 | 14.67 |
| 03/06/2019 12:30 | 14.17 |
| 03/06/2019 14:00 | 13.87 |
| 03/06/2019 14:30 | 13.97 |
| 03/06/2019 15:00 | 14.17 |
| 03/06/2019 15:30 | 14.17 |
| 03/06/2019 16:00 | 14.37 |
| 03/06/2019 16:30 | 19.57 |
| 03/06/2019 17:00 | 15.17 |
| 03/06/2019 17:30 | 15.47 |
| 03/06/2019 18:00 | 14.37 |
| 03/06/2019 18:30 | 14.67 |
| 03/06/2019 19:00 | 14.77 |
| 03/06/2019 19:30 | 15.07 |
| 03/06/2019 20:00 | 15.17 |
| 03/06/2019 20:30 | 15.17 |
| 03/06/2019 21:00 | 15.37 |
| 03/06/2019 21:30 | 15.37 |
| 03/06/2019 22:00 | 15.57 |
| 03/06/2019 22:30 | 16.17 |
| 03/06/2019 23:00 | 16.87 |
| 04/06/2019 05:00 | 17.47 |
| 04/06/2019 11:00 | 18.49 |
| 04/06/2019 17:00 | 18.69 |
| 04/06/2019 19:30 | 17.69 |
| 04/06/2019 20:00 | 18.09 |
| 04/06/2019 20:30 | 17.79 |
| 04/06/2019 21:00 | 18.19 |
| 04/06/2019 21:30 | 17.89 |
| 04/06/2019 22:00 | 18.09 |
| 04/06/2019 22:30 | 17.69 |
| 04/06/2019 23:00 | 17.59 |
| 05/06/2019 05:00 | 19.01 |
| 05/06/2019 06:30 | 19.11 |
| 05/06/2019 11:00 | 19.20 |
| 05/06/2019 12:00 | 19.40 |
| 05/06/2019 13:00 | 19.50 |
| 05/06/2019 14:00 | 19.30 |
| 05/06/2019 15:30 | 19.60 |
| 05/06/2019 17:00 | 20.30 |
| 05/06/2019 18:00 | 20.70 |
| 05/06/2019 19:30 | 21.10 |
| 05/06/2019 20:00 | 21.10 |
| 05/06/2019 20:30 | 20.83 |
| 05/06/2019 21:00 | 20.93 |
| 05/06/2019 21:30 | 21.23 |
| 05/06/2019 22:00 | 20.83 |
| 05/06/2019 22:30 | 21.43 |
| 05/06/2019 23:00 | 21.30 |
| 06/06/2019 05:00 | 20.73 |
| 06/06/2019 11:00 | 20.97 |
| 06/06/2019 17:00 | 19.47 |
| 06/06/2019 23:00 | 18.37 |
| 07/06/2019 05:00 | 20.67 |

**Table S3:** BC subsurface water (SSW) DOC concentration measured by TOC analyser

| Date and Time | DOC (mg/L) |
| --- | --- |
| 04/05/2019 11:00 | 9.33 |
| 04/05/2019 15:00 | 14.62 |
| 04/05/2019 17:00 | 21.02 |
| 05/05/2019 06:30 | 23.54 |
| 05/05/2019 07:30 | 23.24 |
| 05/05/2019 12:00 | 23.14 |
| 05/05/2019 13:35 | 24.51 |
| 05/05/2019 13:59 | 22.91 |
| 05/05/2019 14:25 | 22.81 |
| 05/05/2019 14:55 | 22.71 |
| 05/05/2019 15:25 | 23.11 |
| 05/05/2019 15:55 | 22.51 |
| 05/05/2019 16:25 | 22.41 |
| 05/05/2019 18:00 | 22.44 |
| 05/05/2019 23:20 | 21.71 |
| 06/05/2019 00:00 | 26.00 |
| 06/05/2019 05:25 | 22.01 |
| 06/05/2019 07:00 | 22.40 |
| 06/05/2019 12:00 | 23.60 |
| 07/05/2019 06:00 | 23.00 |
| 07/05/2019 11:30 | 26.41 |
| 07/05/2019 12:00 | 22.40 |
| 07/05/2019 12:00 | 22.81 |
| 07/05/2019 12:30 | 23.01 |
| 07/05/2019 14:30 | 22.21 |
| 07/05/2019 18:00 | 23.81 |
| 07/05/2019 23:00 | 24.61 |
| 08/05/2019 05:00 | 24.21 |
| 08/05/2019 08:00 | 24.11 |
| 08/05/2019 09:00 | 23.81 |
| 08/05/2019 09:30 | 23.81 |
| 08/05/2019 10:00 | 23.61 |
| 08/05/2019 10:30 | 23.31 |
| 08/05/2019 11:00 | 23.81 |
| 08/05/2019 17:00 | 23.71 |
| 08/05/2019 23:00 | 22.38 |
| 09/05/2019 05:00 | 24.78 |
| 09/05/2019 07:00 | 24.88 |
| 09/05/2019 08:00 | 24.88 |
| 09/05/2019 08:30 | 24.98 |
| 09/05/2019 09:00 | 24.78 |
| 09/05/2019 10:00 | 24.68 |
| 09/05/2019 11:00 | 24.38 |
| 09/05/2019 13:00 | 23.78 |
| 09/05/2019 15:10 | 23.48 |
| 09/05/2019 17:00 | 23.78 |
| 09/05/2019 23:00 | 21.82 |
| 10/05/2019 05:00 | 20.72 |
| 13/05/2019 14:00 | 21.83 |
| 13/05/2019 15:00 | 21.73 |
| 13/05/2019 16:00 | 21.83 |
| 13/05/2019 17:00 | 21.73 |
| 13/05/2019 23:00 | 21.73 |
| 14/05/2019 05:00 | 21.83 |
| 14/05/2019 05:30 | 21.43 |
| 14/05/2019 06:00 | 21.73 |
| 14/05/2019 06:30 | 21.73 |
| 14/05/2019 07:00 | 21.33 |
| 14/05/2019 07:30 | 21.63 |
| 14/05/2019 09:00 | 21.23 |
| 14/05/2019 10:00 | 21.23 |
| 14/05/2019 10:30 | 20.53 |
| 14/05/2019 11:00 | 20.53 |
| 14/05/2019 13:30 | 18.79 |
| 14/05/2019 14:00 | 18.89 |
| 14/05/2019 15:30 | 18.89 |
| 14/05/2019 17:00 | 16.04 |
| 14/05/2019 23:00 | 17.64 |
| 15/05/2019 05:00 | 21.04 |
| 15/05/2019 07:00 | 20.64 |
| 15/05/2019 09:00 | 20.24 |
| 15/05/2019 10:00 | 20.72 |
| 15/05/2019 11:00 | 20.62 |
| 15/05/2019 12:00 | 21.02 |
| 15/05/2019 12:30 | 20.82 |
| 15/05/2019 13:00 | 20.72 |
| 15/05/2019 13:30 | 20.62 |
| 15/05/2019 15:00 | 20.72 |
| 15/05/2019 17:00 | 20.52 |
| 15/05/2019 23:00 | 20.72 |
| 16/05/2019 05:00 | 20.32 |
| 16/05/2019 07:00 | 20.42 |
| 16/05/2019 09:00 | 20.99 |
| 16/05/2019 11:00 | 20.39 |
| 16/05/2019 12:00 | 20.99 |
| 16/05/2019 14:00 | 20.79 |
| 16/05/2019 15:30 | 20.89 |
| 16/05/2019 16:30 | 20.79 |
| 16/05/2019 17:00 | 20.49 |
| 17/05/2019 05:00 | 19.19 |
| 17/05/2019 07:00 | 19.19 |
| 17/05/2019 08:00 | 19.09 |
| 17/05/2019 09:30 | 18.57 |
| 17/05/2019 11:00 | 18.97 |
| 17/05/2019 13:00 | 21.07 |
| 17/05/2019 15:00 | 22.67 |
| 17/05/2019 17:00 | 22.67 |
| 17/05/2019 23:00 | 20.77 |
| 18/05/2019 05:00 | 20.29 |
| 21/05/2019 11:00 | 25.27 |
| 21/05/2019 11:50 | 25.67 |
| 21/05/2019 12:20 | 24.97 |
| 21/05/2019 12:50 | 25.07 |
| 21/05/2019 13:20 | 25.07 |
| 21/05/2019 15:00 | 24.27 |
| 21/05/2019 17:00 | 23.67 |
| 21/05/2019 23:00 | 21.77 |
| 22/05/2019 05:00 | 20.51 |
| 22/05/2019 05:50 | 20.21 |
| 22/05/2019 11:00 | 19.76 |
| 22/05/2019 11:50 | 19.56 |
| 22/05/2019 12:45 | 19.46 |
| 22/05/2019 14:00 | 19.36 |
| 22/05/2019 15:45 | 19.06 |
| 22/05/2019 16:20 | 18.76 |
| 22/05/2019 17:00 | 18.46 |
| 22/05/2019 18:05 | 17.96 |
| 22/05/2019 23:00 | 20.96 |
| 23/05/2019 05:00 | 22.42 |
| 23/05/2019 08:00 | 23.28 |
| 23/05/2019 11:00 | 25.18 |
| 23/05/2019 17:00 | 24.48 |
| 23/05/2019 23:00 | 22.58 |
| 24/05/2019 05:00 | 20.38 |
| 24/05/2019 09:00 | 18.37 |
| 24/05/2019 10:00 | 18.17 |
| 24/05/2019 10:30 | 18.17 |
| 24/05/2019 11:00 | 19.13 |
| 24/05/2019 12:00 | 18.17 |
| 24/05/2019 14:00 | 18.77 |
| 24/05/2019 14:30 | 19.17 |
| 24/05/2019 15:15 | 19.77 |
| 24/05/2019 16:00 | 19.67 |
| 24/05/2019 17:00 | 21.03 |
| 24/05/2019 18:00 | 20.77 |
| 24/05/2019 23:00 | 21.07 |
| 25/05/2019 05:00 | 21.79 |
| 25/05/2019 11:00 | 20.29 |
| 25/05/2019 17:00 | 20.45 |
| 25/05/2019 23:00 | 19.25 |
| 26/05/2019 05:00 | 18.15 |
| 26/05/2019 11:00 | 18.37 |
| 26/05/2019 11:35 | 18.27 |
| 26/05/2019 12:15 | 18.17 |
| 26/05/2019 15:05 | 17.87 |
| 26/05/2019 17:00 | 17.87 |
| 26/05/2019 23:00 | 19.87 |
| 27/05/2019 05:00 | 17.77 |
| 27/05/2019 11:00 | 18.63 |
| 27/05/2019 17:00 | 17.93 |
| 27/05/2019 23:00 | 17.13 |
| 28/05/2019 05:00 | 17.23 |
| 31/05/2019 10:00 | 18.72 |
| 31/05/2019 11:00 | 18.62 |
| 31/05/2019 12:00 | 19.02 |
| 31/05/2019 12:30 | 19.32 |
| 31/05/2019 13:00 | 19.32 |
| 31/05/2019 13:30 | 19.42 |
| 31/05/2019 14:00 | 19.32 |
| 31/05/2019 14:30 | 19.82 |
| 31/05/2019 15:00 | 19.82 |
| 31/05/2019 15:30 | 19.82 |
| 31/05/2019 16:00 | 19.72 |
| 31/05/2019 16:30 | 20.02 |
| 31/05/2019 17:00 | 19.52 |
| 31/05/2019 17:30 | 19.92 |
| 31/05/2019 18:00 | 19.43 |
| 31/05/2019 18:30 | 19.73 |
| 31/05/2019 19:00 | 19.93 |
| 31/05/2019 23:00 | 19.82 |
| 01/06/2019 05:00 | 19.59 |
| 01/06/2019 11:00 | 20.00 |
| 01/06/2019 17:00 | 18.80 |
| 01/06/2019 23:00 | 18.00 |
| 02/06/2019 05:00 | 17.30 |
| 02/06/2019 11:00 | 16.44 |
| 02/06/2019 17:00 | 15.74 |
| 02/06/2019 23:00 | 15.34 |
| 03/06/2019 05:00 | 14.64 |
| 03/06/2019 08:00 | 14.44 |
| 03/06/2019 09:00 | 14.10 |
| 03/06/2019 10:00 | 14.30 |
| 03/06/2019 11:00 | 14.00 |
| 03/06/2019 12:30 | 13.70 |
| 03/06/2019 14:00 | 13.60 |
| 03/06/2019 14:30 | 13.70 |
| 03/06/2019 15:00 | 14.00 |
| 03/06/2019 15:30 | 14.30 |
| 03/06/2019 16:00 | 14.40 |
| 03/06/2019 16:30 | 14.70 |
| 03/06/2019 17:00 | 14.50 |
| 03/06/2019 17:30 | 14.90 |
| 03/06/2019 18:00 | 14.90 |
| 03/06/2019 18:30 | 15.40 |
| 03/06/2019 19:00 | 15.30 |
| 03/06/2019 19:30 | 15.40 |
| 03/06/2019 20:00 | 15.80 |
| 03/06/2019 20:30 | 15.37 |
| 03/06/2019 21:00 | 15.37 |
| 03/06/2019 21:30 | 15.47 |
| 03/06/2019 22:00 | 15.47 |
| 03/06/2019 22:30 | 16.17 |
| 03/06/2019 23:00 | 16.30 |
| 04/06/2019 05:00 | 17.67 |
| 04/06/2019 11:00 | 18.38 |
| 04/06/2019 17:00 | 18.78 |
| 04/06/2019 19:30 | 17.89 |
| 04/06/2019 20:00 | 17.79 |
| 04/06/2019 20:30 | 17.79 |
| 04/06/2019 21:00 | 17.89 |
| 04/06/2019 21:30 | 17.69 |
| 04/06/2019 22:00 | 17.59 |
| 04/06/2019 22:30 | 17.69 |
| 04/06/2019 23:00 | 17.88 |
| 05/06/2019 05:00 | 19.09 |
| 05/06/2019 06:30 | 19.09 |
| 05/06/2019 11:00 | 19.42 |
| 05/06/2019 12:00 | 20.02 |
| 05/06/2019 13:00 | 20.32 |
| 05/06/2019 14:00 | 19.72 |
| 05/06/2019 15:30 | 20.22 |
| 05/06/2019 17:00 | 20.72 |
| 05/06/2019 18:00 | 22.90 |
| 05/06/2019 19:30 | 21.20 |
| 05/06/2019 20:00 | 21.60 |
| 05/06/2019 20:30 | 21.10 |
| 05/06/2019 21:00 | 20.90 |
| 05/06/2019 21:30 | 21.00 |
| 05/06/2019 22:00 | 20.80 |
| 05/06/2019 22:30 | 20.90 |
| 05/06/2019 23:00 | 21.72 |
| 06/06/2019 05:00 | 20.93 |
| 06/06/2019 11:00 | 20.47 |
| 06/06/2019 17:00 | 19.67 |
| 06/06/2019 23:00 | 18.47 |
| 07/06/2019 05:00 | 20.37 |

# **C: LC-OCD-OND data**

**Table S4:** Liquid chromatography-organic carbon detection-organic nitrogen detection (LC-OCD-OND) results for DOC, DOM compound groups, and M_n_ in BW SML samples.

| Date and Time | DOC (µg/L) | Biopolymers (µg/L) | Humic Substances (µg/L) | Building Blocks (µg/L) | Low Molecular Weight Neutral (µg/L) | Low Molecular Weight Acids (µg/L) | Nominal Molecular Weight of Humic Substances (g/mol) |
| --- | --- | --- | --- | --- | --- | --- | --- |
| 04/05/2019 11:00 | 8583 | 173 | 5198 | 1324 | 1888 |  | 747.00 |
| 04/05/2019 15:00 | 13103 | 265 | 8501 | 2064 | 2273 |  | 847.00 |
| 04/05/2019 17:00 | 21809 | 307 | 14646 | 2835 | 1341 |  | 1195.00 |
| 05/05/2019 06:30 | 26811 | 319 | 18578 | 3375 | 1530 | 16 | 1219.00 |
| 05/05/2019 07:30 | 26778 | 242 | 17870 | 3312 | 1617 | 76 | 1222.00 |
| 05/05/2019 10:00 | 20110 | 749 | 14011 | 2969 | 2381 |  | 957.00 |
| 05/05/2019 12:00 | 27933 | 1306 | 17769 | 3168 | 2279 | 681 | 1263.00 |
| 05/05/2019 13:35 | 23276 | 796 | 15426 | 2994 | 2190 | 203 | 1206.00 |
| 05/05/2019 13:59 | 25637 | 773 | 17554 | 3186 | 1940 | 177 | 1244.00 |
| 05/05/2019 14:25 | 21340 | 762 | 14263 | 3245 | 3069 |  | 910.00 |
| 05/05/2019 14:55 | 22175 | 949 | 13882 | 3765 | 3579 |  | 946.00 |
| 05/05/2019 15:25 | 21201 | 848 | 13946 | 3643 | 2764 |  | 837.00 |
| 05/05/2019 15:55 | 21980 | 979 | 14411 | 3776 | 2815 |  | 883.00 |
| 05/05/2019 16:25 | 21252 | 880 | 14191 | 3675 | 2506 |  | 881.00 |
| 05/05/2019 23:20 | 9496 | 423 | 6466 | 1562 | 1045 |  | 1116.00 |
| 06/05/2019 00:00 | 25692 | 1188 | 16406 | 4541 | 3556 |  | 965.00 |
| 06/05/2019 05:25 | 6780 | 118 | 5372 | 850 | 441 |  | 1402.00 |
| 06/05/2019 07:00 | 25364 | 1174 | 17437 | 3729 | 3025 |  | 788.00 |
| 06/05/2019 12:00 | 14800 | 589 | 9640 | 2741 | 1830 |  | 1016.00 |
| 06/05/2019 18:00 | 22628 | 1094 | 14378 | 4099 | 3057 |  | 975.00 |
| 07/05/2019 06:00 | 20259 | 470 | 15887 | 2419 | 1483 |  | 1341.00 |
| 07/05/2019 11:30 | 27672 | 850 | 18807 | 2623 | 1367 | 1018 | 1116.00 |
| 07/05/2019 12:00 | 23511 | 1033 | 16521 | 2887 | 1211 | 883 | 1186.00 |
| 07/05/2019 12:00 | 19582 | 299 | 13408 | 3159 | 2716 |  | 849.00 |
| 07/05/2019 12:30 | 10200 | 595 | 7460 | 978 | 640 |  | 1023.00 |
| 07/05/2019 14:30 | 25702 | 683 | 18913 | 2643 | 1374 |  | 1025.00 |
| 07/05/2019 18:00 | 22461 | 728 | 15663 | 3143 | 902 | 739 | 1237.00 |
| 07/05/2019 23:00 | 25911 | 610 | 16945 | 3353 | 1419 | 697 | 1235.00 |
| 08/05/2019 05:00 | 22374 | 465 | 17734 | 2714 | 1461 |  | 1417.00 |
| 08/05/2019 08:00 | 26459 | 860 | 17142 | 2693 | 1169 | 1377 | 1253.00 |
| 08/05/2019 09:00 | 27874 | 966 | 20390 | 3294 | 1815 |  | 1085.00 |
| 08/05/2019 09:30 | 27796 | 1328 | 18343 | 2859 | 1791 | 805 | 1125.00 |
| 08/05/2019 10:00 | 15424 | 766 | 10057 | 2398 | 2203 |  | 927.00 |
| 08/05/2019 10:30 | 20923 | 346 | 13970 | 3651 | 2955 |  | 880.00 |
| 08/05/2019 11:00 | 21506 | 515 | 14509 | 3685 | 2797 |  | 883.00 |
| 08/05/2019 17:00 | 26641 | 659 | 17977 | 2798 | 1111 | 1484 | 1138.00 |
| 08/05/2019 23:00 | 26446 | 601 | 17877 | 2384 | 1257 | 1342 | 1138.00 |
| 09/05/2019 05:00 | 26804 | 713 | 17868 | 2736 | 1353 | 1024 | 1144.00 |
| 09/05/2019 07:00 | 25989 | 657 | 16971 | 2532 | 1259 | 1219 | 1194.00 |
| 09/05/2019 08:00 | 27554 | 502 | 20694 | 3244 | 1434 |  | 1119.00 |
| 09/05/2019 08:30 | 27646 | 1438 | 19248 | 2543 | 1330 | 966 | 1121.00 |
| 09/05/2019 09:00 | 27346 | 1440 | 19031 | 2443 | 1371 | 940 | 1097.00 |
| 09/05/2019 10:00 | 16324 | 731 | 11440 | 2024 | 826 |  | 1246.00 |
| 09/05/2019 11:00 | 21841 | 791 | 14799 | 2027 | 524 | 1113 | 1204.00 |
| 09/05/2019 13:00 | 26002 | 735 | 17424 | 3362 | 1400 | 969 | 1235.00 |
| 09/05/2019 15:10 | 27476 | 1077 | 20205 | 2873 | 1540 |  | 1079.00 |
| 09/05/2019 17:00 | 27300 | 1318 | 18451 | 3017 | 1351 | 1127 | 1225.00 |
| 09/05/2019 23:00 | 19572 | 408 | 13544 | 2331 | 1216 | 851 | 1253.00 |
| 10/05/2019 05:00 | 28076 | 843 | 18156 | 3089 | 1381 | 1302 | 1227.00 |
| 13/05/2019 14:00 | 24502 | 485 | 16325 | 2611 | 1177 | 1181 | 1147.00 |
| 13/05/2019 15:00 | 24639 | 402 | 16515 | 2896 | 1207 | 987 | 1215.00 |
| 13/05/2019 16:00 | 24476 | 581 | 16532 | 2623 | 1072 | 1353 | 1161.00 |
| 13/05/2019 17:00 | 22813 | 717 | 15167 | 2732 | 1069 | 924 | 1198.00 |
| 13/05/2019 23:00 | 24150 | 576 | 16326 | 2605 | 1176 | 1321 | 1157.00 |
| 14/05/2019 05:00 | 29615 | 591 | 16154 | 2168 | 1005 | 1366 | 1197.00 |
| 14/05/2019 05:30 | 20641 | 585 | 13439 | 1962 | 858 | 1317 | 1133.00 |
| 14/05/2019 06:00 | 24789 | 473 | 16327 | 2049 | 1121 | 1208 | 1039.00 |
| 14/05/2019 06:30 | 24078 | 415 | 15974 | 2569 | 847 | 1294 | 1177.00 |
| 14/05/2019 07:00 | 24365 | 1045 | 15807 | 2956 | 1216 | 1158 | 1239.00 |
| 14/05/2019 07:30 | 23909 | 529 | 16380 | 2443 | 1174 | 893 | 1088.00 |
| 14/05/2019 09:00 | 22507 | 974 | 14715 | 2487 | 1217 | 1208 | 1178.00 |
| 14/05/2019 10:00 | 22891 | 1090 | 15797 | 2006 | 1060 | 1390 | 1191.00 |
| 14/05/2019 10:30 | 23759 | 520 | 15584 | 2802 | 1087 | 1109 | 1202.00 |
| 14/05/2019 11:00 | 17687 | 424 | 13178 | 2159 | 931 | 926 | 985.00 |
| 14/05/2019 13:30 | 23602 | 574 | 15257 | 3008 | 1157 | 709 | 1220.00 |
| 14/05/2019 14:00 | 21646 | 1032 | 14457 | 2107 | 1159 | 1276 | 1106.00 |
| 14/05/2019 15:30 | 21091 | 532 | 14559 | 1872 | 768 | 1410 | 1151.00 |
| 14/05/2019 17:00 | 23915 | 490 | 15380 | 2413 | 823 | 1220 | 1151.00 |
| 14/05/2019 23:00 | 18209 | 961 | 12326 | 1617 | 845 | 1278 | 1094.00 |
| 15/05/2019 05:00 | 24365 | 414 | 16707 | 2791 | 1128 | 964 | 1229.00 |
| 15/05/2019 07:00 | 23726 | 1209 | 15641 | 2775 | 1830 | 630 | 1170.00 |
| 15/05/2019 09:00 | 24052 | 589 | 15756 | 2630 | 1068 | 1200 | 1214.00 |
| 15/05/2019 10:00 | 23772 | 637 | 15664 | 2049 | 1104 | 1267 | 1096.00 |
| 15/05/2019 11:00 | 23250 | 568 | 15565 | 2104 | 1036 | 1346 | 1174.00 |
| 15/05/2019 12:00 | 23257 | 1066 | 16007 | 2626 | 1221 | 934 | 1155.00 |
| 15/05/2019 12:30 | 23739 | 1138 | 16420 | 2321 | 1213 | 639 | 1080.00 |
| 15/05/2019 13:00 | 24417 | 1140 | 15982 | 2813 | 1032 | 870 | 1185.00 |
| 15/05/2019 13:30 | 23002 | 681 | 15823 | 2536 | 915 | 802 | 1201.00 |
| 15/05/2019 15:00 | 24554 | 625 | 15779 | 2412 | 861 | 1104 | 1159.00 |
| 15/05/2019 17:00 | 30111 | 833 | 16636 | 2851 | 5090 | 565 | 1144.00 |
| 15/05/2019 23:00 | 23165 | 608 | 15672 | 2067 | 990 | 1326 | 1200.00 |
| 16/05/2019 05:00 | 22754 | 1090 | 15305 | 2254 | 641 | 1245 | 1205.00 |
| 16/05/2019 07:00 | 22865 | 718 | 15603 | 2208 | 1178 | 1317 | 1193.00 |
| 16/05/2019 09:00 | 23341 | 1088 | 16040 | 2533 | 1065 | 1089 | 1211.00 |
| 16/05/2019 11:00 | 23387 | 648 | 15838 | 2079 | 1088 | 1286 | 1167.00 |
| 16/05/2019 12:00 | 20693 | 1116 | 15010 | 2571 | 937 | 941 | 1196.00 |
| 16/05/2019 14:00 | 23596 | 678 | 15830 | 2348 | 1096 | 1389 | 1235.00 |
| 16/05/2019 15:30 | 23837 | 518 | 16076 | 2594 | 1090 | 992 | 1220.00 |
| 16/05/2019 16:30 | 23074 | 656 | 15998 | 2282 | 1044 | 1167 | 1211.00 |
| 16/05/2019 17:00 | 24280 | 645 | 15452 | 2402 | 989 | 1247 | 1245.00 |
| 17/05/2019 05:00 | 23791 | 676 | 16174 | 2457 | 1316 | 868 | 1168.00 |
| 17/05/2019 07:00 | 23459 | 675 | 16066 | 2219 | 1015 | 1113 | 1167.00 |
| 17/05/2019 08:00 | 21515 | 556 | 14823 | 1981 | 1034 | 1302 | 1130.00 |
| 17/05/2019 09:30 | 20439 | 605 | 13798 | 1833 | 884 | 1225 | 1157.00 |
| 17/05/2019 11:00 | 20759 | 976 | 14502 | 2548 | 1135 | 968 | 1171.00 |
| 17/05/2019 13:00 | 20967 | 621 | 13682 | 2174 | 955 | 1155 | 1176.00 |
| 17/05/2019 15:00 | 23524 | 1149 | 15269 | 2732 | 1149 | 1188 | 1294.00 |
| 17/05/2019 17:00 | 22526 | 673 | 15038 | 2118 | 982 | 1159 | 1174.00 |
| 17/05/2019 23:00 | 25141 | 837 | 16862 | 2290 | 1184 | 1199 | 1164.00 |
| 18/05/2019 05:00 | 27241 | 869 | 17968 | 2392 | 1029 | 1278 | 1193.00 |
| 21/05/2019 11:00 | 24652 | 773 | 16171 | 1644 | 978 | 1257 | 1031.00 |
| 21/05/2019 11:50 | 19562 | 300 | 15618 | 2847 | 797 |  | 1337.00 |
| 21/05/2019 12:20 | 25883 | 406 | 20531 | 3378 | 1568 |  | 1297.00 |
| 21/05/2019 12:50 | 14457 | 469 | 11341 | 1658 | 988 |  | 1343.00 |
| 21/05/2019 13:20 | 21712 | 243 | 17306 | 2674 | 1488 |  | 1329.00 |
| 21/05/2019 15:00 | 22305 | 593 | 17131 | 2785 | 1796 |  | 1287.00 |
| 21/05/2019 23:00 | 21577 | 669 | 16995 | 2453 | 1460 |  | 1246.00 |
| 22/05/2019 05:00 | 22381 | 553 | 17687 | 2631 | 1509 |  | 1222.00 |
| 22/05/2019 05:50 | 19745 | 540 | 15827 | 2354 | 1024 |  | 1327.00 |
| 22/05/2019 11:00 | 18097 | 103 | 14658 | 2177 | 1158 | 1 | 1249.00 |
| 22/05/2019 11:50 | 13496 | 217 | 10462 | 1973 | 844 |  | 1361.00 |
| 22/05/2019 12:45 | 18125 | 464 | 14231 | 2379 | 1047 | 4 | 1303.00 |
| 22/05/2019 14:00 | 20664 | 705 | 16000 | 2613 | 1347 |  | 1268.00 |
| 22/05/2019 15:45 | 20271 | 523 | 16252 | 2110 | 1387 |  | 1207.00 |
| 22/05/2019 16:20 | 16851 | 542 | 13362 | 1931 | 1016 |  | 1214.00 |
| 22/05/2019 17:00 | 15968 | 339 | 12560 | 2036 | 1032 |  | 1331.00 |
| 22/05/2019 18:05 | 19687 | 325 | 15358 | 2710 | 1274 | 20 | 1333.00 |
| 22/05/2019 23:00 | 16277 | 227 | 13060 | 2030 | 961 |  | 1257.00 |
| 23/05/2019 05:00 | 19186 | 262 | 15766 | 2057 | 1101 |  | 1205.00 |
| 23/05/2019 08:00 | 23487 | 578 | 18390 | 3157 | 1362 |  | 1322.00 |
| 23/05/2019 11:00 | 24387 | 403 | 19232 | 3076 | 1676 |  | 1327.00 |
| 23/05/2019 17:00 | 25821 | 828 | 20202 | 3104 | 1688 |  | 1341.00 |
| 23/05/2019 23:00 | 8432 | 255 | 6582 | 1048 | 548 |  | 1323.00 |
| 24/05/2019 05:00 | 20875 | 379 | 16783 | 2457 | 1255 |  | 1329.00 |
| 24/05/2019 09:00 | 20822 | 222 | 16932 | 2453 | 1215 |  | 1320.00 |
| 24/05/2019 10:00 | 20291 | 589 | 15695 | 2573 | 1435 |  | 1311.00 |
| 24/05/2019 10:30 | 10767 | 143 | 8523 | 1348 | 754 |  | 1321.00 |
| 24/05/2019 11:00 | 25794 | 911 | 18917 | 3066 | 2831 | 69 | 1319.00 |
| 24/05/2019 12:00 | 20097 | 642 | 14135 | 2637 | 2549 | 133 | 1328.00 |
| 24/05/2019 14:00 | 15481 | 324 | 12048 | 2072 | 1031 | 6 | 1312.00 |
| 24/05/2019 14:30 | 21127 | 686 | 16476 | 2508 | 1457 |  | 1252.00 |
| 24/05/2019 15:15 | 16834 | 415 | 13283 | 2132 | 1004 |  | 1369.00 |
| 24/05/2019 16:00 | 24874 | 962 | 17440 | 3536 | 2840 | 96 | 1398.00 |
| 24/05/2019 17:00 | 17917 | 230 | 14261 | 2286 | 1139 |  | 1308.00 |
| 24/05/2019 18:00 | 21703 | 767 | 16784 | 2714 | 1438 |  | 1319.00 |
| 24/05/2019 23:00 | 18799 | 483 | 14739 | 2414 | 1161 | 2 | 1369.00 |
| 25/05/2019 05:00 | 16888 | 155 | 13657 | 2174 | 902 |  | 1340.00 |
| 25/05/2019 11:00 | 14852 | 149 | 12294 | 1624 | 785 |  | 1365.00 |
| 25/05/2019 17:00 | 23480 | 543 | 18550 | 2904 | 1483 |  | 1347.00 |
| 25/05/2019 23:00 | 19987 | 420 | 15860 | 2514 | 1193 |  | 1357.00 |
| 26/05/2019 05:00 | 6942 | 154 | 5498 | 866 | 424 |  | 1348.00 |
| 26/05/2019 11:00 | 18830 | 130 | 14517 | 2851 | 1331 |  | 1417.00 |
| 26/05/2019 11:35 | 14526 | 400 | 11384 | 1688 | 1054 |  | 1373.00 |
| 26/05/2019 12:15 | 18000 | 263 | 14330 | 2163 | 1245 |  | 1300.00 |
| 26/05/2019 15:05 | 18913 | 288 | 15116 | 2349 | 1161 |  | 1327.00 |
| 26/05/2019 17:00 | 16757 | 549 | 13238 | 1953 | 1017 |  | 1340.00 |
| 26/05/2019 23:00 | 17655 | 519 | 13773 | 2189 | 1174 |  | 1370.00 |
| 27/05/2019 05:00 | 20128 | 680 | 15601 | 2314 | 1533 |  | 1296.00 |
| 27/05/2019 11:00 | 17877 | 594 | 14128 | 2069 | 1086 |  | 1322.00 |
| 27/05/2019 17:00 | 4445 | 108 | 3541 | 525 | 270 |  | 1298.00 |
| 27/05/2019 23:00 | 14789 | 277 | 11678 | 1784 | 1050 |  | 1336.00 |
| 28/05/2019 05:00 | 12164 | 442 | 9579 | 1372 | 771 |  | 1253.00 |
| 31/05/2019 10:00 | 22122 | 817 | 17447 | 2460 | 1397 |  | 1340.00 |
| 31/05/2019 11:00 | 21187 | 427 | 16632 | 2684 | 1444 |  | 1302.00 |
| 31/05/2019 12:00 | 19333 | 252 | 15494 | 2434 | 1153 |  | 1342.00 |
| 31/05/2019 12:30 | 19812 | 457 | 15302 | 2808 | 1245 |  | 1314.00 |
| 31/05/2019 13:00 | 19938 | 421 | 15503 | 2649 | 1365 |  | 1340.00 |
| 31/05/2019 13:30 | 16883 | 498 | 12942 | 2116 | 1326 |  | 1361.00 |
| 31/05/2019 14:00 | 4473 | 146 | 3505 | 531 | 291 |  | 1294.00 |
| 31/05/2019 14:00 | 18773 | 259 | 15070 | 2196 | 1249 |  | 1338.00 |
| 31/05/2019 14:30 | 21025 | 283 | 16834 | 2523 | 1385 |  | 1352.00 |
| 31/05/2019 15:00 | 20476 | 345 | 16509 | 2278 | 1344 |  | 1220.00 |
| 31/05/2019 15:30 | 20588 | 310 | 16259 | 2508 | 1512 |  | 1316.00 |
| 31/05/2019 16:00 | 19437 | 275 | 15239 | 2539 | 1384 |  | 1389.00 |
| 31/05/2019 16:30 | 12970 | 300 | 10133 | 1647 | 890 |  | 1293.00 |
| 31/05/2019 17:00 | 21883 | 352 | 17226 | 2780 | 1525 |  | 1407.00 |
| 31/05/2019 17:30 | 18944 | 297 | 15303 | 2401 | 943 |  | 1334.00 |
| 31/05/2019 18:00 | 21332 | 443 | 16776 | 2752 | 1361 |  | 1296.00 |
| 31/05/2019 18:30 | 20899 | 418 | 16372 | 2643 | 1466 |  | 1300.00 |
| 31/05/2019 19:00 | 19238 | 631 | 14768 | 2379 | 1459 |  | 1292.00 |
| 01/06/2019 05:00 | 21652 | 641 | 17002 | 2463 | 1546 |  | 1280.00 |
| 01/06/2019 11:00 | 23515 | 745 | 18856 | 2673 | 1241 |  | 1222.00 |
| 01/06/2019 17:00 | 20275 | 858 | 15106 | 2133 | 946 | 1233 | 1460.00 |
| 01/06/2019 23:00 | 18885 | 485 | 15009 | 2186 | 1205 |  | 1355.00 |
| 02/06/2019 05:00 | 13722 | 423 | 10815 | 1715 | 769 |  | 1355.00 |
| 02/06/2019 11:00 | 16863 | 592 | 13098 | 2179 | 994 |  | 1358.00 |
| 02/06/2019 17:00 | 7188 | 90 | 5621 | 935 | 542 |  | 1296.00 |
| 02/06/2019 23:00 | 17160 | 497 | 13490 | 2022 | 1150 |  | 1313.00 |
| 03/06/2019 05:00 | 15961 | 198 | 12736 | 1954 | 1073 |  | 1307.00 |
| 03/06/2019 08:00 | 15799 | 259 | 12563 | 1820 | 1158 |  | 1285.00 |
| 03/06/2019 09:00 | 10324 | 369 | 7778 | 1367 | 810 |  | 1357.00 |
| 03/06/2019 10:00 | 15242 | 209 | 12047 | 1798 | 1188 |  | 1198.00 |
| 03/06/2019 11:00 | 14951 | 206 | 11874 | 1904 | 966 |  | 1292.00 |
| 03/06/2019 12:30 | 14336 | 238 | 11431 | 1802 | 865 |  | 1271.00 |
| 03/06/2019 14:00 | 14502 | 240 | 11500 | 1801 | 960 |  | 1216.00 |
| 03/06/2019 14:30 | 14774 | 206 | 11726 | 1743 | 1098 |  | 1223.00 |
| 03/06/2019 15:00 | 9665 | 129 | 7513 | 1212 | 811 |  | 1281.00 |
| 03/06/2019 15:30 | 7298 | 80 | 5183 | 1292 | 743 |  | 949.00 |
| 03/06/2019 16:00 | 13724 | 300 | 11590 | 1835 |  |  | 1206.00 |
| 03/06/2019 16:30 | 3814 | 59 | 3248 | 507 |  |  | 1298.00 |
| 03/06/2019 17:00 | 16030 | 731 | 11980 | 2060 | 1260 |  | 1228.00 |
| 03/06/2019 17:30 | 14997 | 282 | 11872 | 1871 | 972 |  | 1273.00 |
| 03/06/2019 18:00 | 14749 | 294 | 11660 | 1877 | 919 |  | 1284.00 |
| 03/06/2019 18:30 | 18009 | 835 | 14081 | 1919 | 1174 |  | 1208.00 |
| 03/06/2019 19:00 | 9436 | 112 | 7318 | 1254 | 752 |  | 1359.00 |
| 03/06/2019 19:30 | 17440 | 565 | 13613 | 2105 | 1158 |  | 1350.00 |
| 03/06/2019 20:00 | 12118 | 410 | 8992 | 1746 | 952 | 17 | 1286.00 |
| 03/06/2019 20:30 | 16501 | 698 | 12338 | 2032 | 1433 |  | 1200.00 |
| 03/06/2019 21:00 | 16468 | 539 | 12795 | 2034 | 1099 |  | 1333.00 |
| 03/06/2019 21:30 | 16179 | 564 | 12465 | 2003 | 1147 |  | 1293.00 |
| 03/06/2019 22:00 | 16894 | 593 | 13066 | 1979 | 1256 |  | 1275.00 |
| 03/06/2019 22:30 | 16600 | 179 | 13100 | 2075 | 1246 |  | 1238.00 |
| 03/06/2019 23:00 | 17680 | 544 | 13502 | 2279 | 1355 |  | 1312.00 |
| 04/06/2019 05:00 | 18396 | 348 | 14658 | 2254 | 1137 |  | 1343.00 |
| 04/06/2019 11:00 | 18573 | 323 | 14850 | 2324 | 1076 |  | 1329.00 |
| 04/06/2019 17:00 | 21217 | 515 | 16264 | 2444 | 1994 |  | 1338.00 |
| 04/06/2019 19:30 | 18430 | 533 | 14525 | 2093 | 1279 |  | 1318.00 |
| 04/06/2019 20:00 | 18964 | 437 | 14552 | 2686 | 1279 | 9 | 1364.00 |
| 04/06/2019 20:30 | 17313 | 609 | 13295 | 2106 | 1304 |  | 1385.00 |
| 04/06/2019 21:00 | 19347 | 613 | 14992 | 2618 | 1124 |  | 1371.00 |
| 04/06/2019 21:30 | 17839 | 429 | 14250 | 1999 | 1162 |  | 1241.00 |
| 04/06/2019 22:00 | 14716 | 238 | 13145 | 1243 | 76 | 13 | 1252.00 |
| 04/06/2019 22:30 | 17742 | 567 | 13726 | 2202 | 1247 |  | 1354.00 |
| 04/06/2019 23:00 | 20018 | 788 | 15220 | 2358 | 1652 |  | 1308.00 |
| 05/06/2019 06:30 | 7813 | 189 | 6219 | 900 | 505 |  | 1355.00 |
| 05/06/2019 11:00 | 7428 | 112 | 6039 | 786 | 490 |  | 1295.00 |
| 05/06/2019 12:00 | 18270 | 402 | 15444 | 2139 | 285 |  | 1393.00 |
| 05/06/2019 13:00 | 20556 | 442 | 16064 | 2419 | 1632 |  | 1341.00 |
| 05/06/2019 14:00 | 21658 | 596 | 17152 | 2762 | 1148 |  | 1442.00 |
| 05/06/2019 15:30 | 7266 | 254 | 5789 | 860 | 364 |  | 1383.00 |
| 05/06/2019 17:00 | 7371 | 166 | 5733 | 1037 | 435 |  | 1553.00 |
| 05/06/2019 18:00 | 22579 | 578 | 17859 | 2708 | 1434 |  | 1356.00 |
| 05/06/2019 19:30 | 4396 | 170 | 3376 | 531 | 318 |  | 1445.00 |
| 05/06/2019 20:00 | 7620 | 180 | 6176 | 803 | 460 |  | 1305.00 |
| 05/06/2019 20:30 | 17459 | 457 | 13714 | 2108 | 1179 |  | 1401.00 |
| 05/06/2019 21:00 | 7521 | 184 | 5867 | 908 | 561 |  | 1397.00 |
| 05/06/2019 21:30 | 12943 | 173 | 10079 | 1577 | 1115 |  | 1419.00 |
| 05/06/2019 22:00 | 20127 | 442 | 16422 | 2268 | 995 |  | 1381.00 |
| 05/06/2019 22:30 | 23744 | 573 | 19171 | 2508 | 1492 |  | 1372.00 |
| 05/06/2019 23:00 | 22120 | 524 | 17481 | 2732 | 1382 |  | 1410.00 |
| 06/06/2019 05:00 | 21581 | 1028 | 13572 | 3709 | 3272 |  | 941.00 |
| 06/06/2019 11:00 | 20123 | 582 | 15572 | 1827 | 904 | 1238 | 1382.00 |
| 06/06/2019 17:00 | 23266 | 831 | 18282 | 2578 | 1575 |  | 1351.00 |
| 06/06/2019 23:00 | 23762 | 899 | 17479 | 2767 | 2510 | 108 | 1331.00 |
| 07/06/2019 05:00 | 24352 | 1315 | 16463 | 2708 | 1177 | 986 | 1167.00 |

**Table S5:** LC-OCD-OND results for Hydrophobic DOC and Aromaticity BW SML samples.

| Date and Time | Hydrophobic DOC (µg/L) | Aromaticity (L/(mg*m)) |
| --- | --- | --- |
| 04/05/2019 11:00 |  | 7.53 |
| 04/05/2019 15:00 |  | 8.12 |
| 04/05/2019 17:00 | 2680 | 6.58 |
| 05/05/2019 06:30 | 2994 | 6.74 |
| 05/05/2019 07:30 | 3662 | 6.74 |
| 05/05/2019 10:00 |  | 8.72 |
| 05/05/2019 12:00 | 2729 | 7.26 |
| 05/05/2019 13:35 | 1666 | 6.55 |
| 05/05/2019 13:59 | 2007 | 6.66 |
| 05/05/2019 14:25 |  | 8.25 |
| 05/05/2019 14:55 |  | 8.42 |
| 05/05/2019 15:25 |  | 8.30 |
| 05/05/2019 15:55 |  | 8.34 |
| 05/05/2019 16:25 |  | 8.41 |
| 05/05/2019 18:00 |  |  |
| 05/05/2019 23:20 |  | 9.11 |
| 06/05/2019 00:00 |  | 8.60 |
| 06/05/2019 05:25 |  | 6.60 |
| 06/05/2019 07:00 |  | 7.69 |
| 06/05/2019 12:00 |  | 8.63 |
| 06/05/2019 18:00 |  | 8.26 |
| 07/05/2019 06:00 |  | 6.58 |
| 07/05/2019 11:30 | 3005 | 6.83 |
| 07/05/2019 12:00 | 975 | 7.31 |
| 07/05/2019 12:00 |  | 7.91 |
| 07/05/2019 12:30 | 526 | 6.16 |
| 07/05/2019 14:30 | 2088 | 6.11 |
| 07/05/2019 18:00 | 1286 | 7.48 |
| 07/05/2019 23:00 | 2885 | 7.24 |
| 08/05/2019 05:00 |  | 6.64 |
| 08/05/2019 08:00 | 3218 | 6.84 |
| 08/05/2019 09:00 | 1410 | 6.09 |
| 08/05/2019 09:30 | 2671 | 6.71 |
| 08/05/2019 10:00 |  | 8.05 |
| 08/05/2019 10:30 |  | 8.12 |
| 08/05/2019 11:00 |  | 8.14 |
| 08/05/2019 17:00 | 2612 | 6.79 |
| 08/05/2019 23:00 | 2984 | 6.74 |
| 09/05/2019 05:00 | 3110 | 6.78 |
| 09/05/2019 07:00 | 3350 | 7.00 |
| 09/05/2019 08:00 | 1681 | 6.13 |
| 09/05/2019 08:30 | 2120 | 6.84 |
| 09/05/2019 09:00 | 2120 | 6.81 |
| 09/05/2019 10:00 | 1304 | 6.56 |
| 09/05/2019 11:00 | 2588 | 7.16 |
| 09/05/2019 13:00 | 2112 | 7.28 |
| 09/05/2019 15:10 | 1780 | 6.31 |
| 09/05/2019 17:00 | 2036 | 7.06 |
| 09/05/2019 23:00 | 1222 | 7.65 |
| 10/05/2019 05:00 | 3306 | 7.15 |
| 13/05/2019 14:00 | 2723 | 7.20 |
| 13/05/2019 15:00 | 2632 | 7.09 |
| 13/05/2019 16:00 | 2315 | 7.10 |
| 13/05/2019 17:00 | 2205 | 7.23 |
| 13/05/2019 23:00 | 2146 | 7.13 |
| 14/05/2019 05:00 | 8331 | 7.21 |
| 14/05/2019 05:30 | 2480 | 7.04 |
| 14/05/2019 06:00 | 3611 | 6.96 |
| 14/05/2019 06:30 | 2979 | 7.13 |
| 14/05/2019 07:00 | 2184 | 7.26 |
| 14/05/2019 07:30 | 2490 | 7.07 |
| 14/05/2019 09:00 | 1906 | 7.09 |
| 14/05/2019 10:00 | 1549 | 7.01 |
| 14/05/2019 10:30 | 2656 | 7.20 |
| 14/05/2019 11:00 | 69 | 7.17 |
| 14/05/2019 13:30 | 2897 | 7.24 |
| 14/05/2019 14:00 | 1613 | 6.98 |
| 14/05/2019 15:30 | 1950 | 7.07 |
| 14/05/2019 17:00 | 3589 | 7.27 |
| 14/05/2019 23:00 | 1181 | 6.90 |
| 15/05/2019 05:00 | 2360 | 7.08 |
| 15/05/2019 07:00 | 1641 | 7.10 |
| 15/05/2019 09:00 | 2809 | 7.22 |
| 15/05/2019 10:00 | 3051 | 7.12 |
| 15/05/2019 11:00 | 2631 | 7.16 |
| 15/05/2019 12:00 | 1403 | 7.14 |
| 15/05/2019 12:30 | 2007 | 6.87 |
| 15/05/2019 13:00 | 2581 | 7.17 |
| 15/05/2019 13:30 | 2245 | 7.24 |
| 15/05/2019 17:00 | 4136 | 6.84 |
| 15/05/2019 23:00 | 2501 | 6.91 |
| 16/05/2019 05:00 | 2218 | 7.21 |
| 16/05/2019 07:00 | 1842 | 7.13 |
| 16/05/2019 09:00 | 1526 | 7.12 |
| 16/05/2019 11:00 | 2448 | 6.97 |
| 16/05/2019 12:00 | 120 | 7.27 |
| 16/05/2019 14:00 | 2254 | 7.12 |
| 16/05/2019 15:30 | 2568 | 7.05 |
| 16/05/2019 16:30 | 1926 | 7.06 |
| 16/05/2019 17:00 | 3544 | 7.12 |
| 17/05/2019 05:00 | 2300 | 7.07 |
| 17/05/2019 07:00 | 2370 | 6.92 |
| 17/05/2019 08:00 | 1820 | 6.79 |
| 17/05/2019 09:30 | 2095 | 6.88 |
| 17/05/2019 11:00 | 629 | 7.36 |
| 17/05/2019 13:00 | 2380 | 7.41 |
| 17/05/2019 15:00 | 2036 | 7.19 |
| 17/05/2019 17:00 | 2555 | 6.89 |
| 17/05/2019 23:00 | 2769 | 6.86 |
| 18/05/2019 05:00 | 3705 | 6.96 |
| 21/05/2019 11:00 | 3829 | 6.69 |
| 21/05/2019 11:50 |  | 6.42 |
| 21/05/2019 12:20 |  | 6.12 |
| 21/05/2019 12:50 |  | 6.30 |
| 21/05/2019 13:20 |  | 6.20 |
| 21/05/2019 15:00 |  | 6.19 |
| 21/05/2019 17:00 |  | 6.16 |
| 21/05/2019 23:00 |  | 6.16 |
| 22/05/2019 05:00 |  | 6.16 |
| 22/05/2019 05:50 |  | 6.30 |
| 22/05/2019 11:00 |  | 6.14 |
| 22/05/2019 11:50 |  | 6.57 |
| 22/05/2019 12:45 |  | 6.45 |
| 22/05/2019 14:00 |  | 6.28 |
| 22/05/2019 15:45 |  | 6.22 |
| 22/05/2019 16:20 |  | 6.32 |
| 22/05/2019 17:00 |  | 6.53 |
| 22/05/2019 18:05 |  | 6.28 |
| 22/05/2019 23:00 |  | 6.41 |
| 23/05/2019 05:00 |  | 6.20 |
| 23/05/2019 08:00 |  | 6.46 |
| 23/05/2019 11:00 |  | 6.27 |
| 23/05/2019 17:00 |  | 6.38 |
| 23/05/2019 23:00 |  | 6.37 |
| 24/05/2019 05:00 |  | 6.52 |
| 24/05/2019 09:00 |  | 6.60 |
| 24/05/2019 10:00 |  | 6.52 |
| 24/05/2019 10:30 |  | 6.46 |
| 24/05/2019 11:00 |  | 6.37 |
| 24/05/2019 12:00 |  | 6.29 |
| 24/05/2019 14:30 |  | 6.32 |
| 24/05/2019 15:15 |  | 6.50 |
| 24/05/2019 16:00 |  | 6.54 |
| 24/05/2019 18:00 |  | 6.43 |
| 24/05/2019 23:00 |  | 6.59 |
| 25/05/2019 05:00 |  | 6.92 |
| 25/05/2019 11:00 |  | 6.39 |
| 25/05/2019 17:00 |  | 6.56 |
| 25/05/2019 23:00 |  | 6.42 |
| 26/05/2019 05:00 |  | 6.52 |
| 26/05/2019 11:00 |  | 6.72 |
| 26/05/2019 11:35 |  | 6.70 |
| 26/05/2019 12:15 |  | 6.40 |
| 26/05/2019 15:05 |  | 6.50 |
| 26/05/2019 17:00 |  | 6.51 |
| 26/05/2019 23:00 |  | 6.60 |
| 27/05/2019 05:00 |  | 6.42 |
| 27/05/2019 17:00 |  | 6.60 |
| 27/05/2019 23:00 |  | 6.53 |
| 28/05/2019 05:00 |  | 6.56 |
| 31/05/2019 10:00 |  | 6.36 |
| 31/05/2019 11:00 |  | 6.71 |
| 31/05/2019 12:00 |  | 6.69 |
| 31/05/2019 12:30 |  | 6.81 |
| 31/05/2019 13:00 |  | 6.76 |
| 31/05/2019 13:30 |  | 6.89 |
| 31/05/2019 14:00 |  | 6.51 |
| 31/05/2019 14:30 |  | 6.62 |
| 31/05/2019 15:00 |  | 6.56 |
| 31/05/2019 15:30 |  | 6.63 |
| 31/05/2019 16:00 |  | 6.93 |
| 31/05/2019 16:30 |  | 6.73 |
| 31/05/2019 17:00 |  | 6.45 |
| 31/05/2019 17:30 |  | 6.90 |
| 31/05/2019 18:00 |  | 6.68 |
| 31/05/2019 18:30 |  | 6.70 |
| 31/05/2019 19:00 |  | 6.64 |
| 31/05/2019 23:00 |  | 6.36 |
| 01/06/2019 05:00 |  | 6.63 |
| 01/06/2019 11:00 |  | 6.28 |
| 01/06/2019 17:00 |  | 7.27 |
| 01/06/2019 23:00 |  | 6.48 |
| 02/06/2019 05:00 |  | 6.66 |
| 02/06/2019 11:00 |  | 6.63 |
| 02/06/2019 17:00 |  | 6.68 |
| 02/06/2019 23:00 |  | 6.56 |
| 03/06/2019 05:00 |  | 6.62 |
| 03/06/2019 08:00 |  | 6.54 |
| 03/06/2019 09:00 |  | 6.96 |
| 03/06/2019 10:00 |  | 6.64 |
| 03/06/2019 11:00 |  | 6.57 |
| 03/06/2019 12:30 |  | 6.68 |
| 03/06/2019 14:00 |  | 6.63 |
| 03/06/2019 14:30 |  | 6.56 |
| 03/06/2019 15:00 |  | 6.91 |
| 03/06/2019 15:30 |  | 5.02 |
| 03/06/2019 16:00 |  | 6.67 |
| 03/06/2019 16:30 |  | 6.73 |
| 03/06/2019 17:00 |  | 6.57 |
| 03/06/2019 17:30 |  | 6.49 |
| 03/06/2019 18:00 |  | 7.01 |
| 03/06/2019 18:30 |  | 6.41 |
| 03/06/2019 19:00 |  | 6.81 |
| 03/06/2019 19:30 |  | 6.57 |
| 03/06/2019 20:00 |  | 6.48 |
| 03/06/2019 20:30 |  | 6.38 |
| 03/06/2019 21:00 |  | 6.68 |
| 03/06/2019 21:30 |  | 6.47 |
| 03/06/2019 22:00 |  | 6.42 |
| 03/06/2019 22:30 |  | 6.45 |
| 03/06/2019 23:00 |  | 6.69 |
| 04/06/2019 05:00 |  | 6.55 |
| 04/06/2019 11:00 |  | 6.51 |
| 04/06/2019 17:00 |  | 6.57 |
| 04/06/2019 19:30 |  | 6.44 |
| 04/06/2019 20:00 |  | 6.63 |
| 04/06/2019 20:30 |  | 6.58 |
| 04/06/2019 21:00 |  | 6.56 |
| 04/06/2019 21:30 |  | 6.27 |
| 04/06/2019 22:00 |  | 6.32 |
| 04/06/2019 22:30 |  | 6.55 |
| 04/06/2019 23:00 |  | 6.63 |
| 05/06/2019 05:00 |  | 6.43 |
| 05/06/2019 06:30 |  | 6.59 |
| 05/06/2019 11:00 |  | 6.44 |
| 05/06/2019 12:00 |  | 6.67 |
| 05/06/2019 13:00 |  | 6.43 |
| 05/06/2019 14:00 |  | 6.54 |
| 05/06/2019 15:30 |  | 6.51 |
| 05/06/2019 17:00 |  | 6.73 |
| 05/06/2019 18:00 |  | 6.43 |
| 05/06/2019 19:30 |  | 6.87 |
| 05/06/2019 20:00 |  | 6.44 |
| 05/06/2019 20:30 |  | 6.74 |
| 05/06/2019 21:00 |  | 6.82 |
| 05/06/2019 21:30 |  | 6.70 |
| 05/06/2019 22:00 |  | 6.42 |
| 05/06/2019 22:30 |  | 6.38 |
| 05/06/2019 23:00 |  | 6.67 |
| 06/06/2019 05:00 |  | 8.85 |
| 06/06/2019 11:00 |  | 7.20 |
| 06/06/2019 17:00 |  | 6.51 |
| 06/06/2019 23:00 |  | 6.44 |
| 07/06/2019 05:00 | 1703 | 6.81 |

**Table S6:** LC-OCD-OND results for DOC, DOM compound groups, and M_n_ in BW SSW samples.

| Date and Time | DOC (µg/L) | Biopolymers (µg/L) | Humic Substances (µg/L) | Building Blocks (µg/L) | Low Molecular Weight Neutral (µg/L) | Low Molecular Weight Acids (µg/L) | Nominal Molecular Weight of Humic Substances (g/mol) |
| --- | --- | --- | --- | --- | --- | --- | --- |
| 04/05/2019 11:00 | 11863 | 793 | 7577 | 1108 | 596 | 1145 | 841 |
| 04/05/2019 15:00 | 11572 | 532 | 6895 | 1864 | 2131 | 152 | 783 |
| 04/05/2019 17:00 | 28083 | 365 | 18191 | 3475 | 1868 | 98 | 1154 |
| 05/05/2019 06:30 | 26400 | 133 | 18562 | 3209 | 1691 | 179 |  |
| 05/05/2019 07:30 | 21500 | 605 | 16856 | 2719 | 1319 |  | 1377 |
| 05/05/2019 10:00 | 25917 | 859 | 17856 | 2855 | 1762 |  | 1164 |
| 05/05/2019 12:00 | 26250 | 892 | 18528 | 3037 | 1862 |  | 1155 |
| 05/05/2019 13:35 | 28128 | 1038 | 17976 | 3102 | 2355 | 347 | 1179 |
| 05/05/2019 13:59 | 25604 | 907 | 17828 | 3367 | 1613 |  | 1222 |
| 05/05/2019 14:25 | 22149 | 877 | 14310 | 3730 | 3232 |  | 946 |
| 05/05/2019 14:55 | 21235 | 850 | 13814 | 3798 | 2774 |  | 971 |
| 05/05/2019 15:25 | 21812 | 941 | 14185 | 3269 | 3416 |  | 858 |
| 05/05/2019 15:55 | 22382 | 906 | 14718 | 3854 | 2904 |  | 847 |
| 05/05/2019 16:25 | 21698 | 928 | 14200 | 3500 | 3070 |  | 855 |
| 05/05/2019 18:00 | 20576 | 420 | 14476 | 2281 | 1012 | 16 | 1121 |
| 05/05/2019 23:20 | 6813 | 374 | 4410 | 1090 | 938 |  | 1015 |
| 06/05/2019 00:00 | 24813 | 1201 | 16414 | 3694 | 3504 |  | 774 |
| 06/05/2019 05:25 | 23348 | 1060 | 15829 | 2439 | 1564 | 586 | 1171 |
| 06/05/2019 07:00 | 6461 | 340 | 4346 | 964 | 811 |  | 875 |
| 06/05/2019 12:00 | 17577 | 810 | 11733 | 2687 | 2348 |  | 915 |
| 06/05/2019 18:00 | 25516 | 1126 | 16244 | 4503 | 3643 |  | 976 |
| 07/05/2019 06:00 | 24352 | 1034 | 16890 | 2419 | 1223 | 1069 | 1189 |
| 07/05/2019 11:30 | 19169 | 876 | 11411 | 3132 | 3363 | 386 | 918 |
| 07/05/2019 12:00 | 20824 | 422 | 13976 | 2915 | 1142 | 497 | 1277 |
| 07/05/2019 12:00 | 24235 | 1002 | 16757 | 2485 | 1414 | 582 | 1085 |
| 07/05/2019 12:30 | 22634 | 872 | 15039 | 2899 | 3824 |  | 793 |
| 07/05/2019 14:30 | 24593 | 421 | 18200 | 2621 | 1503 |  | 1025 |
| 07/05/2019 18:00 | 25650 | 831 | 18031 | 2440 | 1356 | 629 | 1049 |
| 07/05/2019 23:00 | 26178 | 1289 | 17479 | 2910 | 1370 | 925 | 1185 |
| 08/05/2019 05:00 | 22154 | 1188 | 14647 | 3571 | 2747 |  | 894 |
| 08/05/2019 08:00 | 25917 | 1074 | 15539 | 3132 | 2772 |  | 844 |
| 08/05/2019 09:00 | 18978 | 888 | 13176 | 1941 | 902 | 1030 | 1194 |
| 08/05/2019 09:30 | 20362 | 422 | 13069 | 3801 | 3069 |  | 987 |
| 08/05/2019 10:00 | 21792 | 416 | 14424 | 3822 | 3130 |  | 931 |
| 08/05/2019 10:30 | 21950 | 641 | 14573 | 3734 | 3003 |  | 884 |
| 08/05/2019 11:00 | 20062 | 430 | 13978 | 3486 | 2168 |  | 880 |
| 08/05/2019 17:00 | 27789 | 311 | 19430 | 3157 | 1643 |  | 1111 |
| 08/05/2019 23:00 | 24848 | 760 | 16985 | 2474 | 1149 | 1104 | 1130 |
| 09/05/2019 05:00 | 26393 | 599 | 18278 | 2400 | 1180 | 1196 | 1153 |
| 09/05/2019 07:00 | 27554 | 536 | 19545 | 4099 | 1207 |  | 1179 |
| 09/05/2019 08:00 | 25833 | 846 | 16906 | 3405 | 1217 | 925 | 1237 |
| 09/05/2019 08:30 | 21633 | 568 | 14648 | 2642 | 1059 | 10 | 1193 |
| 09/05/2019 09:00 | 28330 | 842 | 18252 | 3171 | 1289 | 1030 | 1221 |
| 09/05/2019 10:00 | 22128 | 1194 | 15502 | 2501 | 861 | 710 | 1199 |
| 09/05/2019 11:00 | 28976 | 1423 | 18611 | 3248 | 1526 | 1109 | 1220 |
| 09/05/2019 13:00 | 27554 | 562 | 18372 | 3461 | 1246 | 35 | 1170 |
| 09/05/2019 15:10 | 15326 | 382 | 10288 | 1547 | 405 | 963 | 1351 |
| 09/05/2019 17:00 | 26680 | 571 | 18136 | 3184 | 1401 | 853 | 1221 |
| 09/05/2019 23:00 | 26674 | 499 | 18438 | 3216 | 1509 |  | 1177 |
| 10/05/2019 05:00 | 24620 | 553 | 16594 | 3168 | 1808 |  | 1152 |
| 13/05/2019 14:00 | 25017 | 528 | 16415 | 3037 | 1334 | 863 | 1166 |
| 13/05/2019 15:00 | 23459 | 425 | 15907 | 2544 | 1114 | 1140 | 1185 |
| 13/05/2019 16:00 | 22976 | 1026 | 16216 | 2396 | 1077 | 1061 | 1101 |
| 13/05/2019 17:00 | 24678 | 548 | 16183 | 3017 | 1158 | 1101 | 1203 |
| 13/05/2019 23:00 | 24496 | 632 | 16166 | 2660 | 856 | 1221 | 1231 |
| 14/05/2019 05:00 | 16859 | 479 | 11037 | 1217 | 735 | 1182 | 1009 |
| 14/05/2019 05:30 | 23361 | 1033 | 16311 | 2556 | 1129 | 984 | 1159 |
| 14/05/2019 06:00 | 23674 | 590 | 16060 | 2361 | 1171 | 1419 | 1163 |
| 14/05/2019 06:30 | 22820 | 546 | 15446 | 2229 | 979 | 1163 | 1108 |
| 14/05/2019 07:00 | 23622 | 542 | 16263 | 2633 | 1243 | 875 | 1165 |
| 14/05/2019 07:30 | 23987 | 1119 | 16636 | 2336 | 1134 | 872 | 1092 |
| 14/05/2019 09:00 | 23113 | 540 | 16322 | 1699 | 1048 | 1232 | 1076 |
| 14/05/2019 10:00 | 22846 | 507 | 16165 | 2692 | 1125 | 889 | 1198 |
| 14/05/2019 10:30 | 23374 | 498 | 15148 | 2610 | 1067 | 1166 | 1206 |
| 14/05/2019 11:00 | 23015 | 456 | 15070 | 2443 | 849 | 1127 | 1176 |
| 14/05/2019 13:30 | 5417 | 232 | 3313 | 867 | 1004 | 1 | 768 |
| 14/05/2019 14:00 | 21626 | 951 | 14856 | 2260 | 1242 | 570 | 1029 |
| 14/05/2019 15:30 | 20133 | 509 | 13401 | 1916 | 1080 | 1249 | 1074 |
| 14/05/2019 17:00 | 18104 | 516 | 11760 | 2571 | 979 | 769 | 1165 |
| 14/05/2019 23:00 | 19930 | 1073 | 12869 | 2403 | 1023 | 1102 | 1148 |
| 15/05/2019 05:00 | 22383 | 1045 | 15941 | 2323 | 894 | 611 | 1064 |
| 15/05/2019 07:00 | 24489 | 1162 | 15627 | 2685 | 1028 | 1156 | 1191 |
| 15/05/2019 09:00 | 23289 | 643 | 15131 | 2432 | 986 | 1239 | 1210 |
| 15/05/2019 10:00 | 23309 | 652 | 15398 | 2379 | 1077 | 1276 | 1238 |
| 15/05/2019 11:00 | 22643 | 538 | 15810 | 1988 | 922 | 1410 | 1137 |
| 15/05/2019 12:00 | 22415 | 1138 | 15459 | 2268 | 658 | 1265 | 1209 |
| 15/05/2019 12:30 | 22774 | 599 | 15505 | 2512 | 1184 | 1097 | 1172 |
| 15/05/2019 13:00 | 23472 | 606 | 15704 | 2067 | 761 | 1317 | 1168 |
| 15/05/2019 13:30 | 23002 | 1311 | 16510 | 2077 | 1250 | 656 | 1001 |
| 15/05/2019 17:00 | 24052 | 718 | 15840 | 1888 | 1134 | 1401 | 1130 |
| 15/05/2019 23:00 | 23452 | 595 | 16103 | 2437 | 1113 | 730 | 1149 |
| 16/05/2019 05:00 | 24430 | 574 | 15950 | 2993 | 1378 | 875 | 1205 |
| 16/05/2019 07:00 | 23980 | 574 | 16354 | 2328 | 1138 | 883 | 1152 |
| 16/05/2019 09:00 | 23857 | 698 | 15624 | 2991 | 1296 | 723 | 1162 |
| 16/05/2019 11:00 | 23791 | 674 | 15895 | 2826 | 1296 | 612 | 1184 |
| 16/05/2019 12:00 | 24241 | 1257 | 16269 | 2571 | 1135 | 1013 | 1216 |
| 16/05/2019 14:00 | 22872 | 1053 | 15346 | 2684 | 984 | 1060 | 1219 |
| 16/05/2019 15:30 | 23543 | 1241 | 16774 | 2211 | 1158 | 812 | 1087 |
| 16/05/2019 16:30 | 22957 | 620 | 15832 | 2095 | 1017 | 1257 | 1161 |
| 16/05/2019 17:00 | 22102 | 976 | 15372 | 2661 | 1269 | 924 | 1164 |
| 17/05/2019 05:00 | 21789 | 505 | 14693 | 2052 | 719 | 1055 | 1141 |
| 17/05/2019 07:00 | 20022 | 649 | 14299 | 2227 | 939 | 1105 | 1162 |
| 17/05/2019 08:00 | 20967 | 463 | 14535 | 1961 | 897 | 1269 | 1128 |
| 17/05/2019 09:30 | 21567 | 610 | 14449 | 2076 | 917 | 1128 | 1162 |
| 17/05/2019 11:00 | 21333 | 648 | 14499 | 2046 | 1036 | 1199 | 1161 |
| 17/05/2019 13:00 | 23765 | 727 | 15911 | 2289 | 1190 | 1126 | 1168 |
| 17/05/2019 15:00 | 26335 | 911 | 17573 | 2451 | 1200 | 1227 | 1183 |
| 17/05/2019 17:00 | 23563 | 682 | 16695 | 2306 | 724 | 1133 | 1156 |
| 17/05/2019 23:00 | 23485 | 786 | 15809 | 2466 | 1067 | 968 | 1158 |
| 18/05/2019 05:00 | 22852 | 742 | 15737 | 1919 | 1017 | 1214 | 1090 |
| 21/05/2019 11:00 | 25054 | 876 | 19186 | 3112 | 1880 |  | 1269 |
| 21/05/2019 11:50 | 24164 | 808 | 18576 | 3187 | 1593 |  | 1336 |
| 21/05/2019 12:20 | 27626 | 313 | 20778 | 3398 | 3055 | 83 | 1292 |
| 21/05/2019 12:50 | 23240 | 512 | 18298 | 2704 | 1714 | 12 | 1269 |
| 21/05/2019 13:20 | 25199 | 449 | 19802 | 3167 | 1782 |  | 1257 |
| 21/05/2019 15:00 | 23865 | 669 | 18333 | 3086 | 1778 |  | 1341 |
| 21/05/2019 17:00 | 4826 | 216 | 3594 | 651 | 365 |  | 1272 |
| 21/05/2019 23:00 | 16353 | 503 | 12873 | 2050 | 926 |  | 1307 |
| 22/05/2019 05:00 | 16693 | 451 | 13163 | 1984 | 1096 |  | 1281 |
| 22/05/2019 05:50 | 18508 | 501 | 14379 | 2302 | 1325 |  | 1354 |
| 22/05/2019 11:00 | 11670 | 283 | 9116 | 1397 | 874 |  | 1382 |
| 22/05/2019 11:50 | 21218 | 707 | 16280 | 2788 | 1443 |  | 1288 |
| 22/05/2019 12:45 | 20105 | 285 | 15751 | 2433 | 1636 |  | 1274 |
| 22/05/2019 14:00 | 20392 | 724 | 15557 | 2782 | 1330 |  | 1306 |
| 22/05/2019 15:45 | 16550 | 481 | 12886 | 2041 | 1143 |  | 1341 |
| 22/05/2019 16:20 | 15458 | 408 | 12231 | 1721 | 1098 |  | 1252 |
| 22/05/2019 17:00 | 19252 | 512 | 15097 | 2633 | 985 | 25 | 1310 |
| 22/05/2019 18:05 | 5119 | 105 | 3612 | 798 | 587 | 17 | 1298 |
| 22/05/2019 23:00 | 22569 | 348 | 18031 | 2711 | 1479 |  | 1306 |
| 23/05/2019 05:00 | 16167 | 281 | 12789 | 2055 | 1041 |  | 1323 |
| 23/05/2019 08:00 | 21229 | 790 | 16456 | 2721 | 1262 |  | 1370 |
| 23/05/2019 11:00 | 22281 | 502 | 17625 | 2746 | 1409 |  | 1324 |
| 23/05/2019 17:00 | 25853 | 446 | 20494 | 3384 | 1529 |  | 1320 |
| 23/05/2019 23:00 | 24818 | 860 | 18971 | 3058 | 1929 |  | 1241 |
| 24/05/2019 05:00 | 21427 | 206 | 16950 | 2740 | 1531 |  | 1324 |
| 24/05/2019 09:00 | 17642 | 161 | 14229 | 2241 | 1010 |  | 1224 |
| 24/05/2019 10:00 | 19891 | 220 | 15862 | 2526 | 1284 |  | 1319 |
| 24/05/2019 10:30 | 16451 | 412 | 13222 | 2139 | 664 | 15 | 1376 |
| 24/05/2019 11:00 | 20275 | 193 | 15642 | 3023 | 1383 | 34 | 1388 |
| 24/05/2019 12:00 | 18550 | 507 | 14618 | 2460 | 964 |  | 1335 |
| 24/05/2019 14:30 | 15835 | 324 | 12524 | 1928 | 1060 |  | 1401 |
| 24/05/2019 15:15 | 7621 | 176 | 6170 | 867 | 407 |  | 1330 |
| 24/05/2019 16:00 | 13293 | 364 | 10446 | 1655 | 829 |  | 1356 |
| 24/05/2019 18:00 | 21970 | 621 | 17218 | 2678 | 1452 |  | 1347 |
| 24/05/2019 23:00 | 26805 | 376 | 21656 | 3110 | 1663 |  | 1333 |
| 25/05/2019 05:00 | 22046 | 195 | 18230 | 2331 | 1290 |  | 1298 |
| 25/05/2019 11:00 | 20702 | 188 | 16832 | 2362 | 1320 |  | 1325 |
| 25/05/2019 17:00 | 20998 | 307 | 16805 | 2543 | 1343 |  | 1339 |
| 25/05/2019 23:00 | 18309 | 190 | 14731 | 2252 | 1136 |  | 1308 |
| 26/05/2019 05:00 | 13966 | 132 | 11266 | 1777 | 790 |  | 1332 |
| 26/05/2019 11:00 | 13732 | 211 | 11126 | 1540 | 855 |  | 1291 |
| 26/05/2019 11:35 | 5511 | 104 | 4315 | 695 | 398 |  | 1353 |
| 26/05/2019 12:15 | 17488 | 175 | 14185 | 2032 | 1095 |  | 1301 |
| 26/05/2019 15:05 | 20122 | 593 | 15830 | 2411 | 1288 |  | 1308 |
| 26/05/2019 17:00 | 13953 | 403 | 11191 | 1470 | 889 |  | 1249 |
| 26/05/2019 23:00 | 3810 | 69 | 2991 | 476 | 274 |  | 1406 |
| 27/05/2019 05:00 | 20125 | 500 | 16021 | 2346 | 1258 |  | 1367 |
| 27/05/2019 17:00 | 4720 | 162 | 3693 | 559 | 306 |  | 1293 |
| 27/05/2019 23:00 | 10503 | 395 | 8243 | 1182 | 680 | 2 | 1265 |
| 28/05/2019 05:00 | 6201 | 193 | 4879 | 737 | 393 |  | 1298 |
| 31/05/2019 10:00 | 14696 | 433 | 11558 | 1781 | 925 |  | 1344 |
| 31/05/2019 11:00 | 20610 | 692 | 15883 | 2468 | 1567 |  | 1334 |
| 31/05/2019 12:00 | 20305 | 454 | 16072 | 2668 | 1110 |  | 1409 |
| 31/05/2019 12:30 | 20614 | 339 | 16243 | 2726 | 1306 |  | 1412 |
| 31/05/2019 13:00 | 13208 | 280 | 10363 | 1574 | 991 |  | 1368 |
| 31/05/2019 13:30 | 20947 | 465 | 16612 | 2516 | 1353 |  | 1351 |
| 31/05/2019 14:00 | 21397 | 446 | 17097 | 2492 | 1362 |  | 1351 |
| 31/05/2019 14:30 | 17035 | 597 | 13068 | 2046 | 1324 |  | 1374 |
| 31/05/2019 15:00 | 17961 | 362 | 14173 | 2243 | 1183 |  | 1430 |
| 31/05/2019 15:30 | 20119 | 311 | 16150 | 2368 | 1290 |  | 1340 |
| 31/05/2019 16:00 | 11148 | 220 | 8692 | 1334 | 902 |  | 1364 |
| 31/05/2019 16:30 | 21771 | 583 | 17207 | 2544 | 1437 |  | 1371 |
| 31/05/2019 17:00 | 18813 | 360 | 15086 | 2303 | 1065 |  | 1356 |
| 31/05/2019 17:30 | 21411 | 698 | 16765 | 2557 | 1391 |  | 1343 |
| 31/05/2019 18:00 | 20555 | 221 | 16365 | 2653 | 1316 |  | 1427 |
| 31/05/2019 18:30 | 19436 | 652 | 15255 | 2299 | 1230 |  | 1361 |
| 31/05/2019 19:00 | 18011 | 596 | 14221 | 1994 | 1200 |  | 1350 |
| 31/05/2019 23:00 | 16311 | 344 | 12936 | 2033 | 999 |  | 1323 |
| 01/06/2019 05:00 | 18006 | 730 | 13932 | 2033 | 1312 |  | 1288 |
| 01/06/2019 11:00 | 22011 | 958 | 16726 | 2804 | 1523 |  | 1381 |
| 01/06/2019 17:00 | 20277 | 858 | 15083 | 2042 | 923 | 1371 | 1456 |
| 01/06/2019 23:00 | 18751 | 883 | 13980 | 1784 | 765 | 1339 | 1427 |
| 02/06/2019 05:00 | 12307 | 527 | 8979 | 1128 | 512 | 1161 | 1376 |
| 02/06/2019 11:00 | 12217 | 365 | 9575 | 1437 | 840 |  | 1352 |
| 02/06/2019 17:00 | 17152 | 334 | 13475 | 2257 | 1086 |  | 1368 |
| 02/06/2019 23:00 | 17233 | 548 | 13373 | 2064 | 1248 |  | 1303 |
| 03/06/2019 05:00 | 2213 | 52 | 1725 | 257 | 180 |  | 1320 |
| 03/06/2019 08:00 | 15161 | 198 | 12167 | 1849 | 947 |  | 1328 |
| 03/06/2019 09:00 | 15985 | 452 | 12329 | 2100 | 1097 | 8 | 1357 |
| 03/06/2019 10:00 | 14079 | 367 | 10987 | 1768 | 957 |  | 1317 |
| 03/06/2019 11:00 | 15544 | 465 | 12316 | 1871 | 893 |  | 1286 |
| 03/06/2019 12:30 | 14243 | 248 | 11265 | 1750 | 979 |  | 1203 |
| 03/06/2019 14:00 | 14916 | 321 | 11726 | 1836 | 1033 |  | 1268 |
| 03/06/2019 14:30 | 11168 | 198 | 8849 | 1359 | 763 |  | 1216 |
| 03/06/2019 15:00 | 14704 | 231 | 11828 | 1667 | 978 |  | 1185 |
| 03/06/2019 15:30 | 15082 | 493 | 11723 | 1848 | 1018 |  | 1223 |
| 03/06/2019 16:00 | 14365 | 237 | 11865 | 2262 |  |  | 1346 |
| 03/06/2019 16:30 | 14441 | 322 | 12165 | 1954 |  |  | 1278 |
| 03/06/2019 17:00 | 5084 | 91 | 4002 | 643 | 348 |  | 1284 |
| 03/06/2019 17:30 | 15313 | 304 | 12051 | 1847 | 1112 |  | 1212 |
| 03/06/2019 18:00 | 16188 | 606 | 12279 | 2068 | 1235 |  | 1260 |
| 03/06/2019 18:30 | 16444 | 588 | 12650 | 1981 | 1226 |  | 1333 |
| 03/06/2019 19:00 | 16364 | 366 | 12901 | 1919 | 1177 |  | 1207 |
| 03/06/2019 19:30 | 16062 | 580 | 12513 | 2093 | 876 |  | 1342 |
| 03/06/2019 20:00 | 8704 | 355 | 6598 | 1085 | 666 |  | 1296 |
| 03/06/2019 20:30 | 16673 | 218 | 13153 | 2207 | 1080 | 15 | 1368 |
| 03/06/2019 21:00 | 18699 | 732 | 14483 | 2365 | 1119 |  | 1246 |
| 03/06/2019 21:30 | 16275 | 489 | 12903 | 1935 | 948 |  | 1309 |
| 03/06/2019 22:00 | 17049 | 581 | 13745 | 1804 | 919 |  | 1283 |
| 03/06/2019 22:30 | 17048 | 376 | 13522 | 2084 | 1067 |  | 1311 |
| 03/06/2019 23:00 | 17306 | 265 | 12398 | 2229 | 2319 | 94 | 1402 |
| 04/06/2019 05:00 | 19383 | 600 | 15188 | 2342 | 1253 |  | 1362 |
| 04/06/2019 11:00 | 20267 | 738 | 15738 | 2479 | 1313 |  | 1346 |
| 04/06/2019 17:00 | 16155 | 634 | 12239 | 2011 | 1272 |  | 1386 |
| 04/06/2019 19:30 | 20004 | 505 | 15700 | 2525 | 1274 |  | 1336 |
| 04/06/2019 20:00 | 19583 | 433 | 15368 | 2377 | 1405 |  | 1310 |
| 04/06/2019 20:30 | 19455 | 402 | 15153 | 2341 | 1559 |  | 1347 |
| 04/06/2019 21:00 | 19889 | 650 | 15440 | 2447 | 1353 |  | 1352 |
| 04/06/2019 21:30 | 19966 | 730 | 15312 | 2432 | 1492 |  | 1297 |
| 04/06/2019 22:00 | 19351 | 446 | 15067 | 2359 | 1479 |  | 1310 |
| 04/06/2019 22:30 | 17824 | 607 | 13862 | 2139 | 1215 |  | 1319 |
| 04/06/2019 23:00 | 6223 | 257 | 4859 | 762 | 346 |  | 1296 |
| 05/06/2019 05:00 | 13888 | 335 | 11147 | 1608 | 798 |  | 1329 |
| 05/06/2019 06:30 | 486 | 19 | 267 | 73 | 127 |  | 763 |
| 05/06/2019 11:00 | 6945 | 140 | 5519 | 817 | 469 |  | 1311 |
| 05/06/2019 12:00 | 7203 | 233 | 5602 | 865 | 504 |  | 1389 |
| 05/06/2019 13:00 | 7350 | 290 | 5859 | 731 | 471 |  | 1250 |
| 05/06/2019 14:00 | 21618 | 487 | 17221 | 2603 | 1307 |  | 1387 |
| 05/06/2019 15:30 | 7418 | 198 | 5783 | 924 | 512 |  | 1376 |
| 05/06/2019 17:00 | 7203 | 197 | 5650 | 855 | 501 |  | 1402 |
| 05/06/2019 18:00 | 22387 | 617 | 17632 | 2534 | 1604 |  | 1371 |
| 05/06/2019 19:30 | 21564 | 433 | 17099 | 2576 | 1457 |  | 1326 |
| 05/06/2019 20:00 | 22055 | 774 | 16861 | 1762 | 1067 | 1590 | 1401 |
| 05/06/2019 20:30 | 7553 | 213 | 5911 | 964 | 464 |  | 1442 |
| 05/06/2019 21:00 | 22805 | 418 | 18044 | 2828 | 1514 |  | 1417 |
| 05/06/2019 21:30 | 5027 | 141 | 3889 | 667 | 330 |  | 1394 |
| 05/06/2019 22:00 | 23343 | 923 | 18219 | 2932 | 1269 |  | 1465 |
| 05/06/2019 22:30 | 23253 | 629 | 18423 | 2738 | 1464 |  | 1399 |
| 05/06/2019 23:00 | 16389 | 234 | 12955 | 2110 | 1090 |  | 1432 |
| 06/06/2019 05:00 | 21213 | 936 | 16549 | 2593 | 1136 |  | 1429 |
| 06/06/2019 11:00 | 25212 | 1048 | 17987 | 3204 | 2935 | 38 | 1401 |
| 06/06/2019 17:00 | 22470 | 453 | 16508 | 2725 | 2709 | 74 | 1337 |
| 06/06/2019 23:00 | 20777 | 673 | 16432 | 2334 | 1339 |  | 1317 |
| 07/06/2019 05:00 | 25091 | 386 | 18783 | 2920 | 2993 | 9 | 1391 |

**Table S7:** LC-OCD-OND results for Hydrophobic DOC and Aromaticity BW SSW samples.

| Date and Time | Hydrophobic DOC (µg/L) | Aromaticity (L/(mg*m)) |
| --- | --- | --- |
| 04/05/2019 11:00 | 643 | 6.33 |
| 04/05/2019 15:00 |  | 7.88 |
| 04/05/2019 17:00 | 4085 | 6.59 |
| 05/05/2019 06:30 | 2626 | 7.78 |
| 05/05/2019 07:30 |  | 6.52 |
| 05/05/2019 10:00 | 2586 | 6.61 |
| 05/05/2019 12:00 | 1931 | 6.53 |
| 05/05/2019 13:35 | 3310 | 6.51 |
| 05/05/2019 13:59 | 1890 | 6.68 |
| 05/05/2019 14:25 |  | 8.58 |
| 05/05/2019 14:55 |  | 8.71 |
| 05/05/2019 15:25 |  | 8.35 |
| 05/05/2019 15:55 |  | 8.22 |
| 05/05/2019 16:25 |  | 8.39 |
| 05/05/2019 18:00 | 2372 | 6.54 |
| 05/05/2019 23:20 |  | 8.69 |
| 06/05/2019 00:00 |  | 8.13 |
| 06/05/2019 05:25 | 1871 | 7.03 |
| 06/05/2019 07:00 |  | 9.34 |
| 06/05/2019 12:00 |  | 8.31 |
| 06/05/2019 18:00 |  | 8.65 |
| 07/05/2019 06:00 | 1718 | 6.93 |
| 07/05/2019 11:30 |  | 7.99 |
| 07/05/2019 12:00 | 1871 | 7.82 |
| 07/05/2019 12:00 | 1995 | 6.78 |
| 07/05/2019 12:30 |  | 7.93 |
| 07/05/2019 14:30 | 1850 | 6.16 |
| 07/05/2019 18:00 | 2364 | 6.70 |
| 07/05/2019 23:00 | 2205 | 6.97 |
| 08/05/2019 05:00 |  | 8.05 |
| 08/05/2019 08:00 | 3401 | 7.52 |
| 08/05/2019 09:00 | 1041 | 6.88 |
| 08/05/2019 09:30 |  | 8.58 |
| 08/05/2019 10:00 |  | 8.23 |
| 08/05/2019 10:30 |  | 8.00 |
| 08/05/2019 11:00 |  | 7.96 |
| 08/05/2019 17:00 | 3249 | 6.27 |
| 08/05/2019 23:00 | 2376 | 6.76 |
| 09/05/2019 05:00 | 2740 | 6.79 |
| 09/05/2019 07:00 | 2168 | 6.27 |
| 09/05/2019 08:00 | 2534 | 7.59 |
| 09/05/2019 08:30 | 2706 | 6.51 |
| 09/05/2019 09:00 | 3747 | 7.16 |
| 09/05/2019 10:00 | 1359 | 7.75 |
| 09/05/2019 11:00 | 3060 | 7.01 |
| 09/05/2019 13:00 | 3878 | 6.40 |
| 09/05/2019 15:10 | 1741 | 7.42 |
| 09/05/2019 17:00 | 2536 | 6.98 |
| 09/05/2019 23:00 | 3013 | 6.37 |
| 10/05/2019 05:00 | 2496 | 6.24 |
| 13/05/2019 14:00 | 2840 | 7.26 |
| 13/05/2019 15:00 | 2329 | 7.04 |
| 13/05/2019 16:00 | 1199 | 7.00 |
| 13/05/2019 17:00 | 2671 | 7.24 |
| 13/05/2019 23:00 | 2960 | 7.26 |
| 14/05/2019 05:00 | 2209 | 6.97 |
| 14/05/2019 05:30 | 1349 | 7.06 |
| 14/05/2019 06:00 | 2072 | 7.05 |
| 14/05/2019 06:30 | 2456 | 7.05 |
| 14/05/2019 07:00 | 2065 | 7.06 |
| 14/05/2019 07:30 | 1890 | 6.96 |
| 14/05/2019 09:00 | 2272 | 6.90 |
| 14/05/2019 10:00 | 1467 | 7.10 |
| 14/05/2019 10:30 | 2885 | 7.12 |
| 14/05/2019 11:00 | 3071 | 7.19 |
| 14/05/2019 13:30 |  | 7.74 |
| 14/05/2019 14:00 | 1746 | 6.89 |
| 14/05/2019 15:30 | 1977 | 7.07 |
| 14/05/2019 17:00 | 1509 | 7.53 |
| 14/05/2019 23:00 | 1461 | 7.45 |
| 15/05/2019 05:00 | 1569 | 7.33 |
| 15/05/2019 07:00 | 2830 | 7.25 |
| 15/05/2019 09:00 | 2858 | 7.20 |
| 15/05/2019 10:00 | 2527 | 7.14 |
| 15/05/2019 11:00 | 1976 | 7.06 |
| 15/05/2019 12:00 | 1627 | 6.98 |
| 15/05/2019 12:30 | 1877 | 7.27 |
| 15/05/2019 13:00 | 3017 | 7.08 |
| 15/05/2019 13:30 | 1199 | 6.87 |
| 15/05/2019 17:00 | 3071 | 6.87 |
| 15/05/2019 23:00 | 2474 | 7.02 |
| 16/05/2019 05:00 | 2660 | 7.16 |
| 16/05/2019 07:00 | 2704 | 7.02 |
| 16/05/2019 09:00 | 2524 | 7.27 |
| 16/05/2019 11:00 | 2488 | 7.13 |
| 16/05/2019 12:00 | 1995 | 7.04 |
| 16/05/2019 14:00 | 1746 | 7.43 |
| 16/05/2019 15:30 | 1348 | 6.89 |
| 16/05/2019 16:30 | 2135 | 6.98 |
| 16/05/2019 17:00 | 901 | 7.40 |
| 17/05/2019 05:00 | 2765 | 7.10 |
| 17/05/2019 07:00 | 803 | 7.10 |
| 17/05/2019 08:00 | 1843 | 7.10 |
| 17/05/2019 09:30 | 2388 | 6.99 |
| 17/05/2019 11:00 | 1905 | 6.95 |
| 17/05/2019 13:00 | 2522 | 6.96 |
| 17/05/2019 15:00 | 2972 | 6.94 |
| 17/05/2019 17:00 | 2024 | 7.21 |
| 17/05/2019 23:00 | 2388 | 7.01 |
| 18/05/2019 05:00 | 2223 | 6.75 |
| 21/05/2019 11:00 |  | 6.36 |
| 21/05/2019 11:50 |  | 6.09 |
| 21/05/2019 12:20 |  | 6.21 |
| 21/05/2019 12:50 |  | 6.18 |
| 21/05/2019 13:20 |  | 6.08 |
| 21/05/2019 15:00 |  | 6.19 |
| 21/05/2019 17:00 |  | 6.44 |
| 21/05/2019 23:00 |  | 6.29 |
| 22/05/2019 05:00 |  | 6.36 |
| 22/05/2019 05:50 |  | 6.38 |
| 22/05/2019 11:00 |  | 6.69 |
| 22/05/2019 11:50 |  | 6.32 |
| 22/05/2019 12:45 |  | 6.33 |
| 22/05/2019 14:00 |  | 6.32 |
| 22/05/2019 15:45 |  | 6.41 |
| 22/05/2019 16:20 |  | 6.39 |
| 22/05/2019 17:00 |  | 6.27 |
| 22/05/2019 18:05 |  | 6.24 |
| 22/05/2019 23:00 |  | 6.28 |
| 23/05/2019 05:00 |  | 6.35 |
| 23/05/2019 08:00 |  | 6.60 |
| 23/05/2019 11:00 |  | 6.42 |
| 23/05/2019 17:00 |  | 6.37 |
| 23/05/2019 23:00 |  | 6.29 |
| 24/05/2019 05:00 |  | 6.28 |
| 24/05/2019 09:00 |  | 6.49 |
| 24/05/2019 10:00 |  | 6.62 |
| 24/05/2019 10:30 |  | 6.55 |
| 24/05/2019 11:00 |  | 6.55 |
| 24/05/2019 12:00 |  | 6.57 |
| 24/05/2019 14:30 |  | 7.19 |
| 24/05/2019 15:15 |  | 6.47 |
| 24/05/2019 16:00 |  | 6.56 |
| 24/05/2019 18:00 |  | 6.51 |
| 24/05/2019 23:00 |  | 6.41 |
| 25/05/2019 05:00 |  | 6.40 |
| 25/05/2019 11:00 |  | 6.32 |
| 25/05/2019 17:00 |  | 6.33 |
| 25/05/2019 23:00 |  | 6.36 |
| 26/05/2019 05:00 |  | 6.59 |
| 26/05/2019 11:00 |  | 6.64 |
| 26/05/2019 11:35 |  | 6.60 |
| 26/05/2019 12:15 |  | 6.41 |
| 26/05/2019 15:05 |  | 6.45 |
| 26/05/2019 17:00 |  | 6.49 |
| 26/05/2019 23:00 |  | 6.75 |
| 27/05/2019 05:00 |  | 6.55 |
| 27/05/2019 17:00 |  | 6.51 |
| 27/05/2019 23:00 |  | 6.58 |
| 28/05/2019 05:00 |  | 6.50 |
| 31/05/2019 10:00 |  | 6.50 |
| 31/05/2019 11:00 |  | 6.47 |
| 31/05/2019 12:00 |  | 6.56 |
| 31/05/2019 12:30 |  | 6.50 |
| 31/05/2019 13:00 |  | 6.63 |
| 31/05/2019 13:30 |  | 6.56 |
| 31/05/2019 14:00 |  | 6.40 |
| 31/05/2019 14:30 |  | 6.54 |
| 31/05/2019 15:00 |  | 6.75 |
| 31/05/2019 15:30 |  | 6.69 |
| 31/05/2019 16:00 |  | 6.60 |
| 31/05/2019 16:30 |  | 6.47 |
| 31/05/2019 17:00 |  | 6.54 |
| 31/05/2019 17:30 |  | 6.49 |
| 31/05/2019 18:00 |  | 6.56 |
| 31/05/2019 18:30 |  | 6.46 |
| 31/05/2019 19:00 |  | 6.43 |
| 31/05/2019 23:00 |  | 6.50 |
| 01/06/2019 05:00 |  | 6.66 |
| 01/06/2019 11:00 |  | 6.52 |
| 01/06/2019 17:00 |  | 7.26 |
| 01/06/2019 23:00 |  | 7.27 |
| 02/06/2019 05:00 |  | 7.32 |
| 02/06/2019 11:00 |  | 6.66 |
| 02/06/2019 17:00 |  | 6.76 |
| 02/06/2019 23:00 |  | 6.64 |
| 03/06/2019 05:00 |  | 6.86 |
| 03/06/2019 08:00 |  | 6.54 |
| 03/06/2019 09:00 |  | 6.72 |
| 03/06/2019 10:00 |  | 6.53 |
| 03/06/2019 11:00 |  | 6.67 |
| 03/06/2019 12:30 |  | 6.74 |
| 03/06/2019 14:00 |  | 6.70 |
| 03/06/2019 14:30 |  | 6.76 |
| 03/06/2019 15:00 |  | 6.45 |
| 03/06/2019 15:30 |  | 6.65 |
| 03/06/2019 16:00 |  | 6.90 |
| 03/06/2019 16:30 |  | 6.60 |
| 03/06/2019 17:00 |  | 6.55 |
| 03/06/2019 17:30 |  | 6.72 |
| 03/06/2019 18:00 |  | 6.86 |
| 03/06/2019 18:30 |  | 6.59 |
| 03/06/2019 19:00 |  | 6.69 |
| 03/06/2019 19:30 |  | 6.47 |
| 03/06/2019 20:00 |  | 6.41 |
| 03/06/2019 20:30 |  | 6.58 |
| 03/06/2019 21:00 |  | 6.34 |
| 03/06/2019 21:30 |  | 6.52 |
| 03/06/2019 22:00 |  | 6.44 |
| 03/06/2019 22:30 |  | 6.54 |
| 03/06/2019 23:00 |  | 6.57 |
| 04/06/2019 05:00 |  | 6.51 |
| 04/06/2019 11:00 |  | 6.66 |
| 04/06/2019 17:00 |  | 6.69 |
| 04/06/2019 19:30 |  | 6.57 |
| 04/06/2019 20:00 |  | 6.57 |
| 04/06/2019 20:30 |  | 6.68 |
| 04/06/2019 21:00 |  | 6.71 |
| 04/06/2019 21:30 |  | 6.57 |
| 04/06/2019 22:00 |  | 6.60 |
| 04/06/2019 22:30 |  | 6.42 |
| 04/06/2019 23:00 |  | 6.85 |
| 05/06/2019 05:00 |  | 6.55 |
| 05/06/2019 06:30 |  | 5.09 |
| 05/06/2019 11:00 |  | 6.66 |
| 05/06/2019 12:00 |  | 6.63 |
| 05/06/2019 13:00 |  | 6.33 |
| 05/06/2019 14:00 |  | 6.49 |
| 05/06/2019 15:30 |  | 6.58 |
| 05/06/2019 17:00 |  | 6.64 |
| 05/06/2019 18:00 |  | 6.52 |
| 05/06/2019 19:30 |  | 6.65 |
| 05/06/2019 20:00 |  | 7.16 |
| 05/06/2019 20:30 |  | 6.67 |
| 05/06/2019 21:00 |  | 6.64 |
| 05/06/2019 21:30 |  | 6.85 |
| 05/06/2019 22:00 |  | 6.61 |
| 05/06/2019 22:30 |  | 6.55 |
| 05/06/2019 23:00 |  | 6.65 |
| 06/06/2019 05:00 |  | 6.59 |
| 06/06/2019 11:00 |  | 6.58 |
| 06/06/2019 17:00 |  | 6.37 |
| 06/06/2019 23:00 |  | 6.41 |
| 07/06/2019 05:00 |  | 6.38 |

# **D: Box and whisker plots**


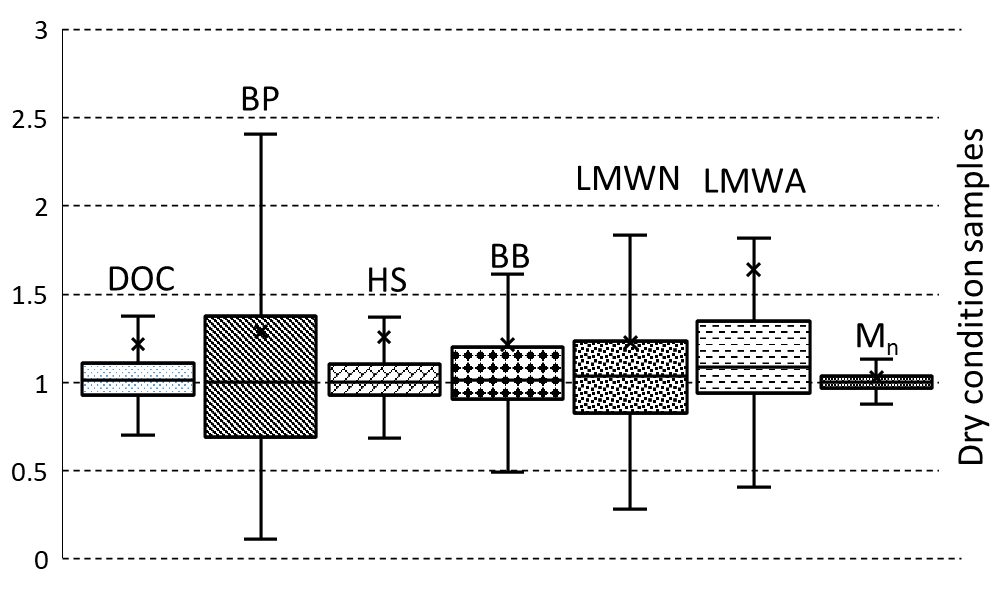


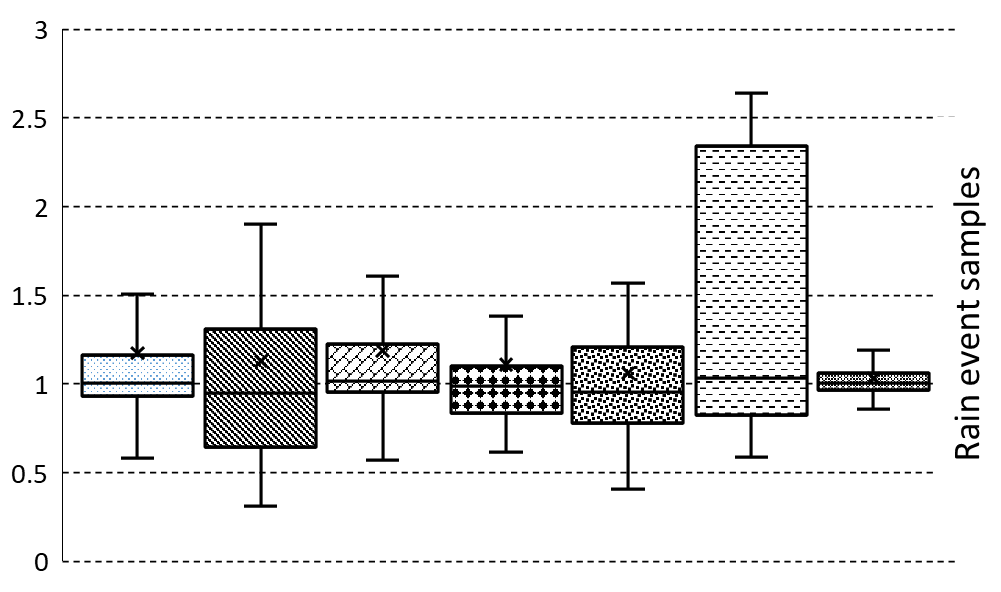


**Figure S1:** Box and whisker plot of DOC, DOM compound groups and HS M_n_ for rain event and dry condition samples.


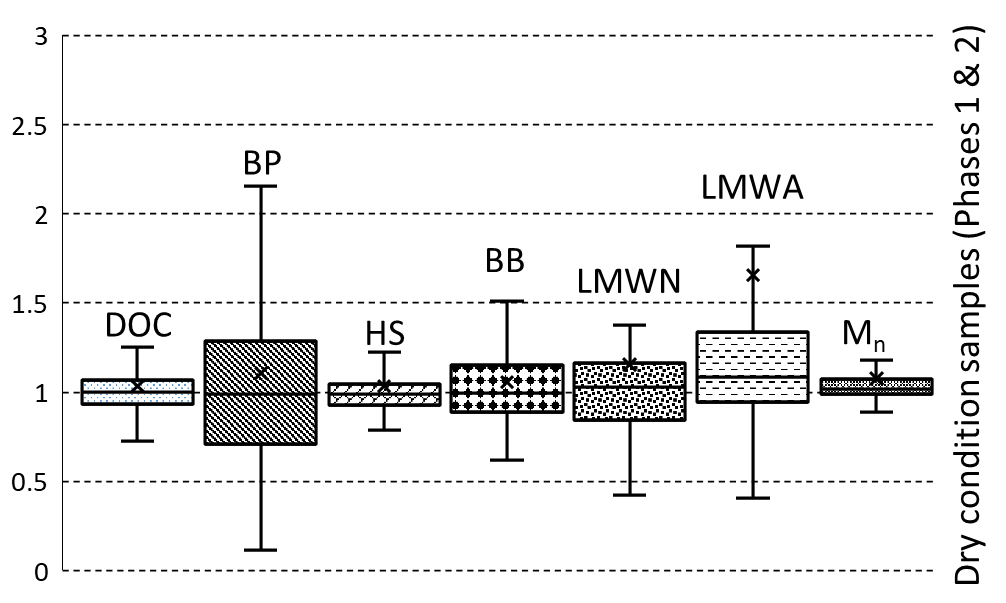


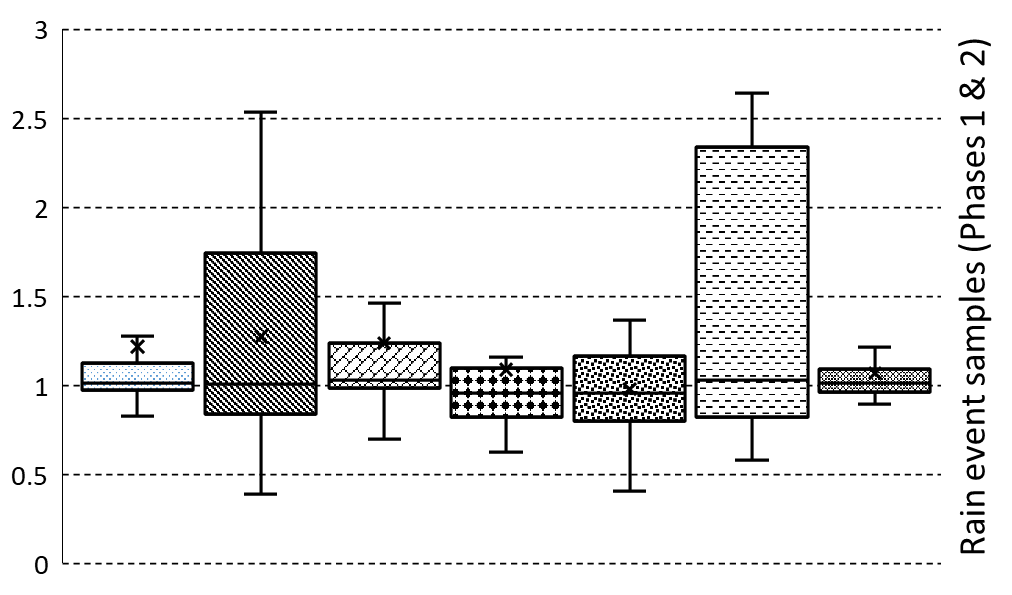


**Figure S2:** Box and whisker plot of DOC, DOM compound groups and HS M_n_ for rain event and dry condition samples of phases 1 & 2.


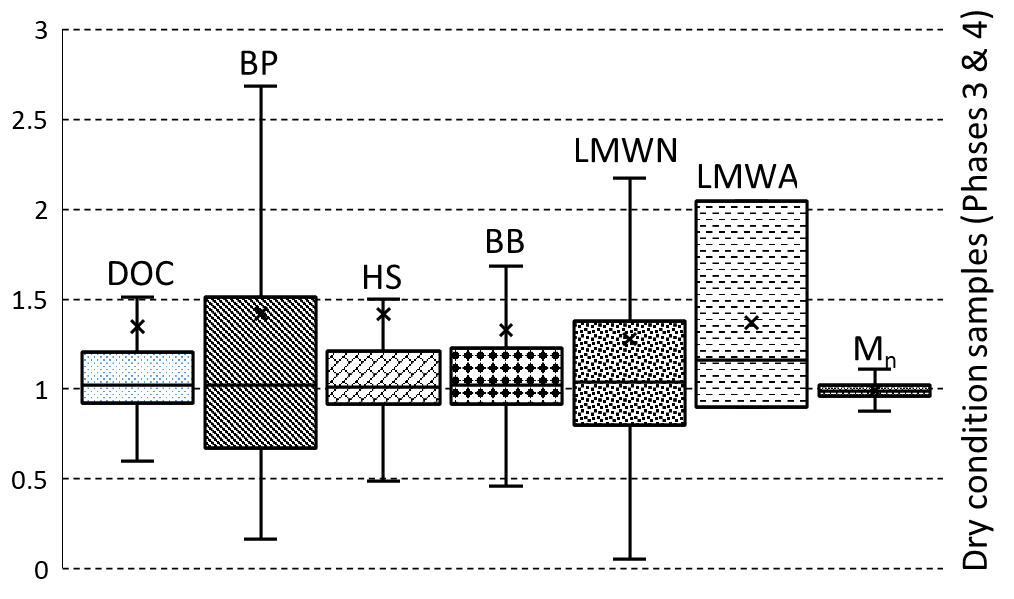


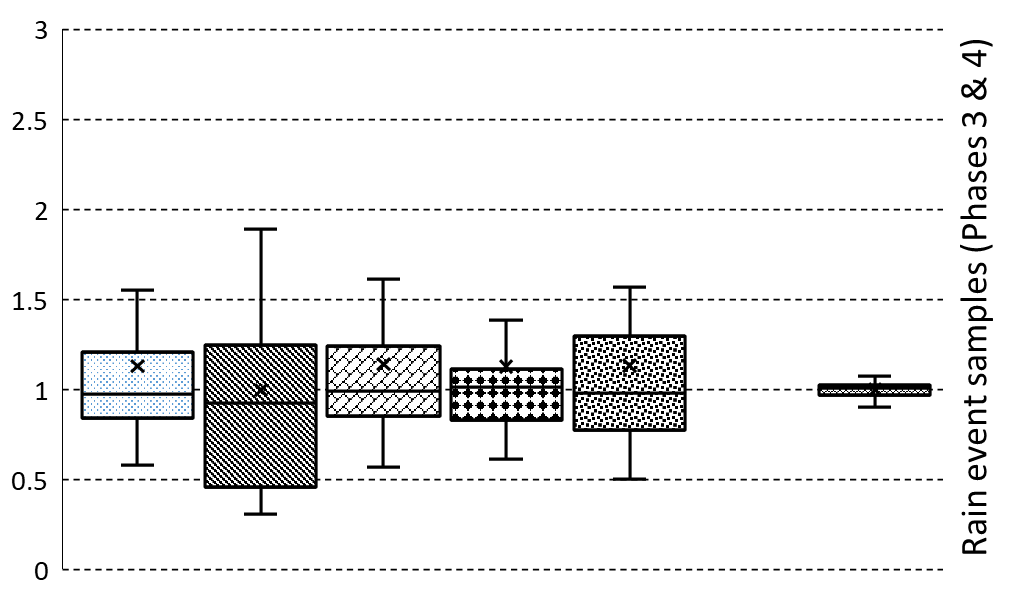


**Figure S3:** Box and whisker plot of DOC, DOM compound groups and HS M_n_ for rain event and dry condition samples of phases 3 & 4.

# **E: Mann-Kendall test results**

**Table S8:** Mann-Kendal test results of concentration of DOM compound groups of SML and SSW

|  |  | Concentration | | | | |
| --- | --- | --- | --- | --- | --- | --- |
|  |  | BP | HS | BB | LMWN | LMWA |
| SML | n | 235 | 235 | 235 | 233 | 235 |
|  | α | 0.05 | 0.05 | 0.05 | 0.05 | 0.05 |
|  | MK-stat | -2167 | 4799 | -3691 | -5285 | 186 |
|  | s.e. | 1205 | 1205 | 1205 | 1189 | 1050 |
|  | z-stat | -1.80 | 3.98 | -3.06 | -4.44 | 0.18 |
|  | p-value | 0.07 | 0.00 | 0.00 | 0.00 | 0.86 |
|  | trend | no | yes | yes | yes | no |
| SSW | n | 234 | 234 | 234 | 232 | 234 |
|  | α | 0.05 | 0.05 | 0.05 | 0.05 | 0.05 |
|  | MK-stat | -3501 | -1361 | -4723 | -4925 | 109 |
|  | s.e. | 1197 | 1197 | 1197 | 1182 | 1051 |
|  | z-stat | -2.92 | -1.14 | -3.94 | -4.17 | 0.10 |
|  | p-value | 0.00 | 0.26 | 0.00 | 0.00 | 0.92 |
|  | trend | yes | no | yes | yes | no |

**Table S9:** Mann–Kendall test results for DOC concentration, M_n_, and percentage contributions of DOM compound groups in SML and SSW.

|  |  | Percentage of total DOM | | | | |  |  |
| --- | --- | --- | --- | --- | --- | --- | --- | --- |
|  |  | BP | HS | BB | LMWN | LMWA | M_n_ | DOC |
| SML | n | 235 | 235 | 235 | 235 | 235 | 235 | 235 |
|  | α | 0.05 | 0.05 | 0.05 | 0.05 | 0.05 | 0.05 | 0.05 |
|  | MK-stat | -2225 | 4883 | -3703 | -3653 | 20 | 5595 | -2987 |
|  | s.e. | 1205 | 1205 | 1205 | 1205 | 1050 | 1205 | 1205 |
|  | z-stat | -1.85 | 4.05 | -3.07 | -3.03 | 0.02 | 4.64 | -2.48 |
|  | p-value | 0.06 | 0.00 | 0.00 | 0.00 | 0.99 | 0.00 | 0.01 |
|  | trend | no | yes | yes | yes | no | yes | yes |
| SSW | n | 234 | 234 | 234 | 234 | 234 | 233 | 234 |
|  | α | 0.05 | 0.05 | 0.05 | 0.05 | 0.05 | 0.05 | 0.05 |
|  | MK-stat | -3375 | 5109 | -3935 | -3633 | 115 | 6259 | -3295 |
|  | s.e. | 1197 | 1197 | 1197 | 1197 | 1051 | 1189 | 1197 |
|  | z-stat | -2.82 | 4.27 | -3.29 | -3.03 | 0.11 | 5.26 | -2.75 |
|  | p-value | 0.00 | 0.00 | 0.00 | 0.00 | 0.91 | 0.00 | 0.01 |
|  | trend | yes | yes | yes | yes | no | yes | yes |

# **F:** **DOM compound groups temporal changes**


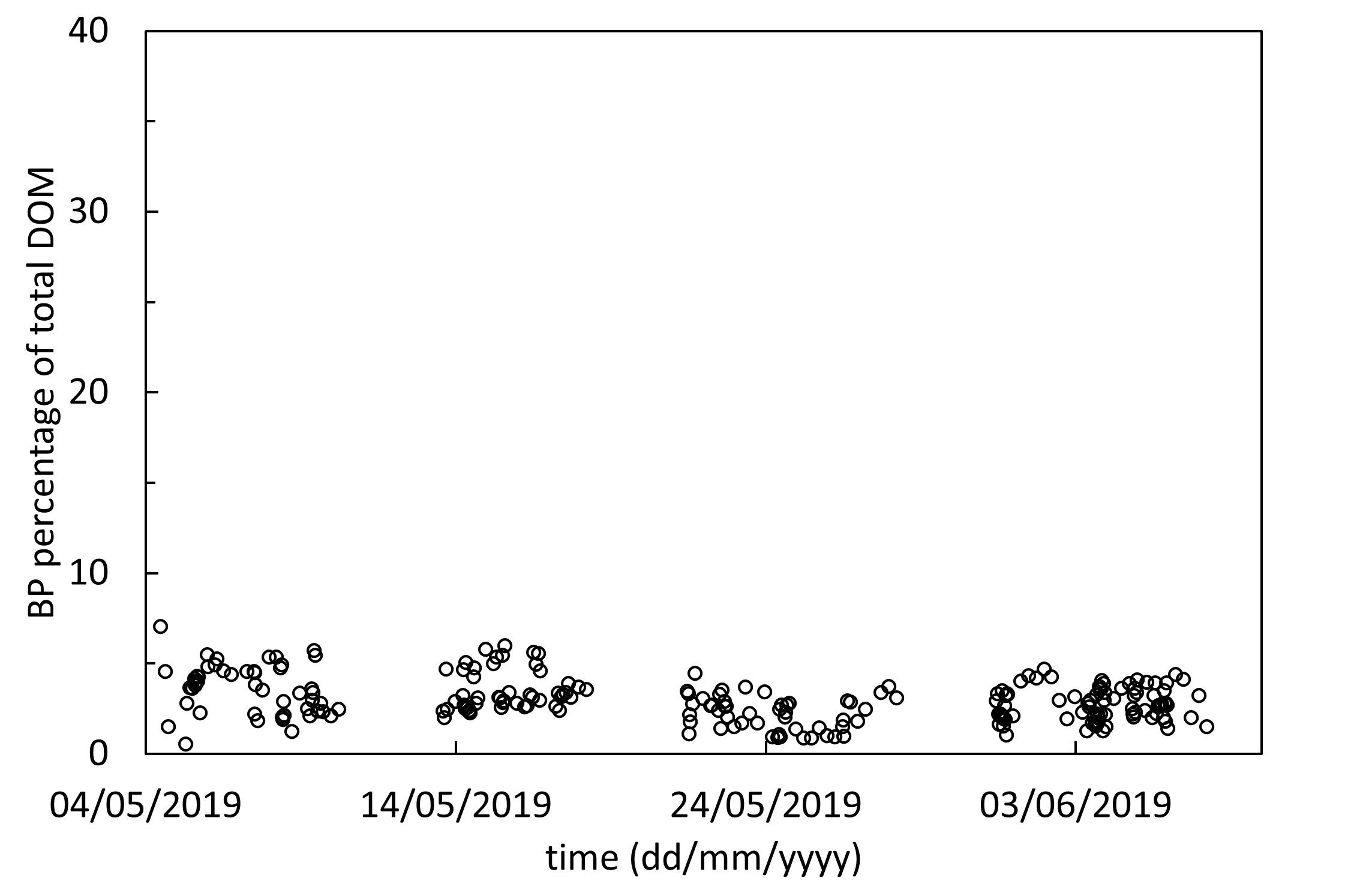


**Figure S4:** SSW temporal changes of biopolymers percentage of total DOM


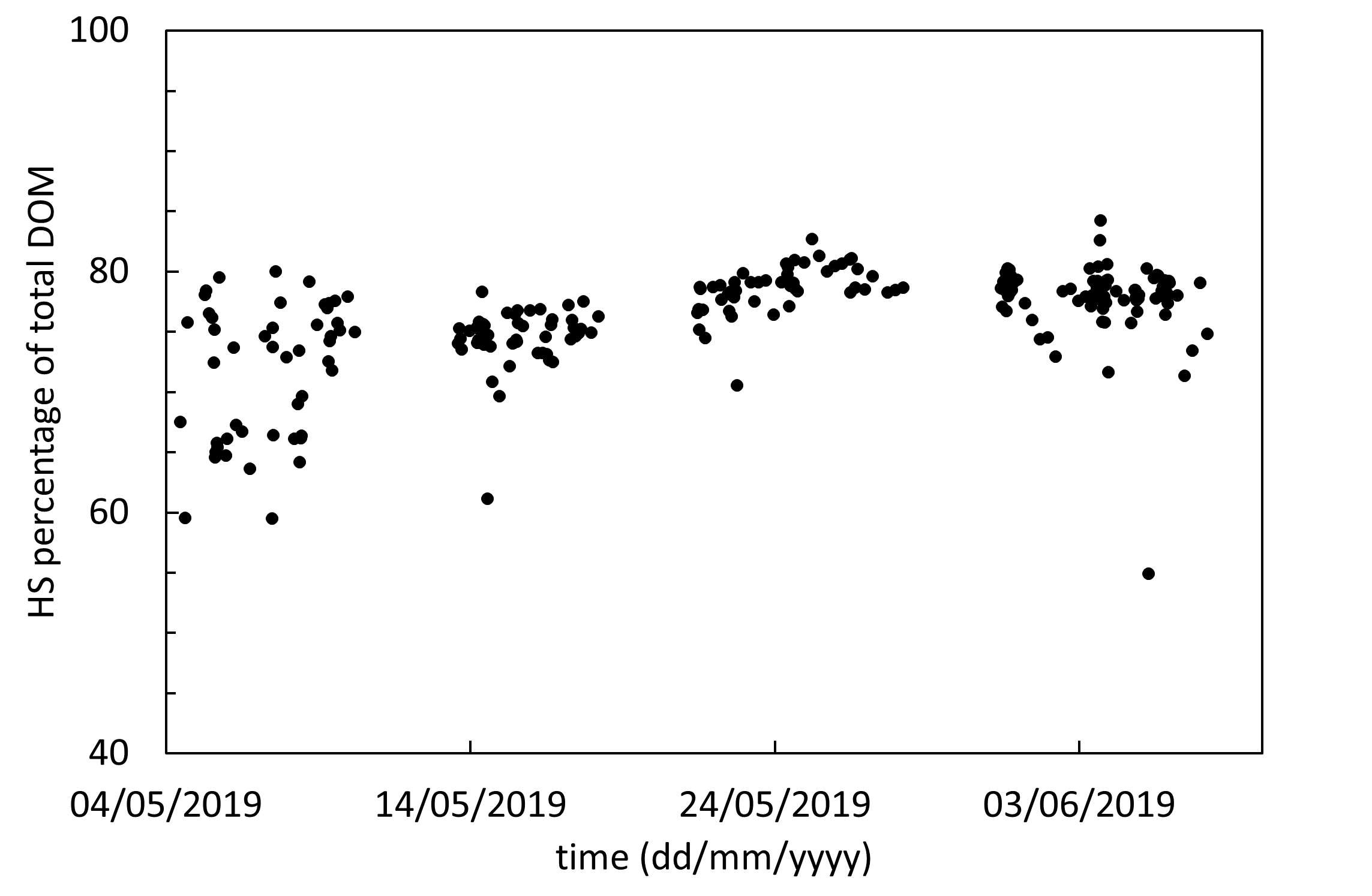


**Figure S5:** SSW temporal changes of humic substances percentage of total DOM


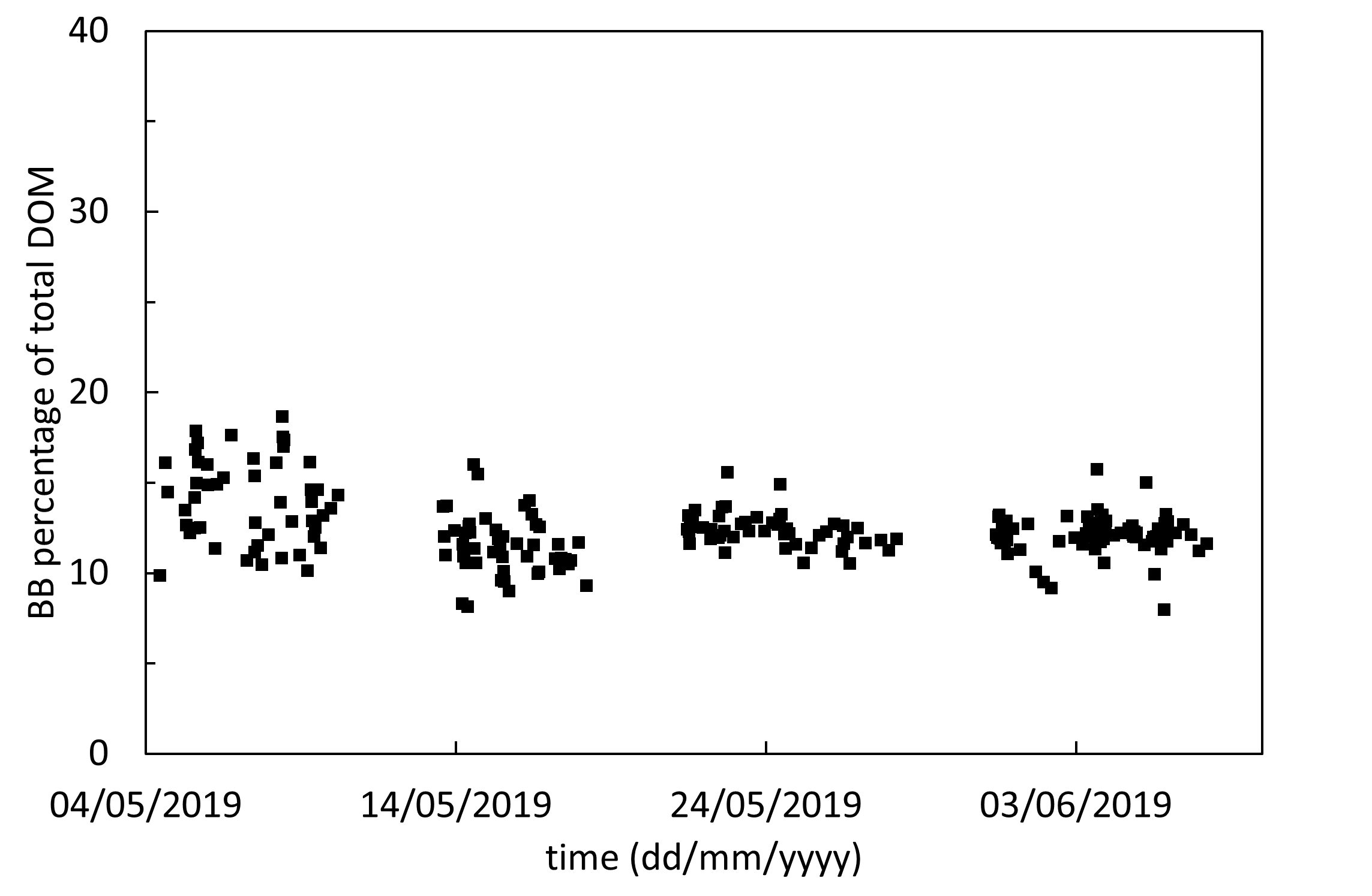


**Figure S6:** SSW temporal changes of building blocks percentage of total DOM


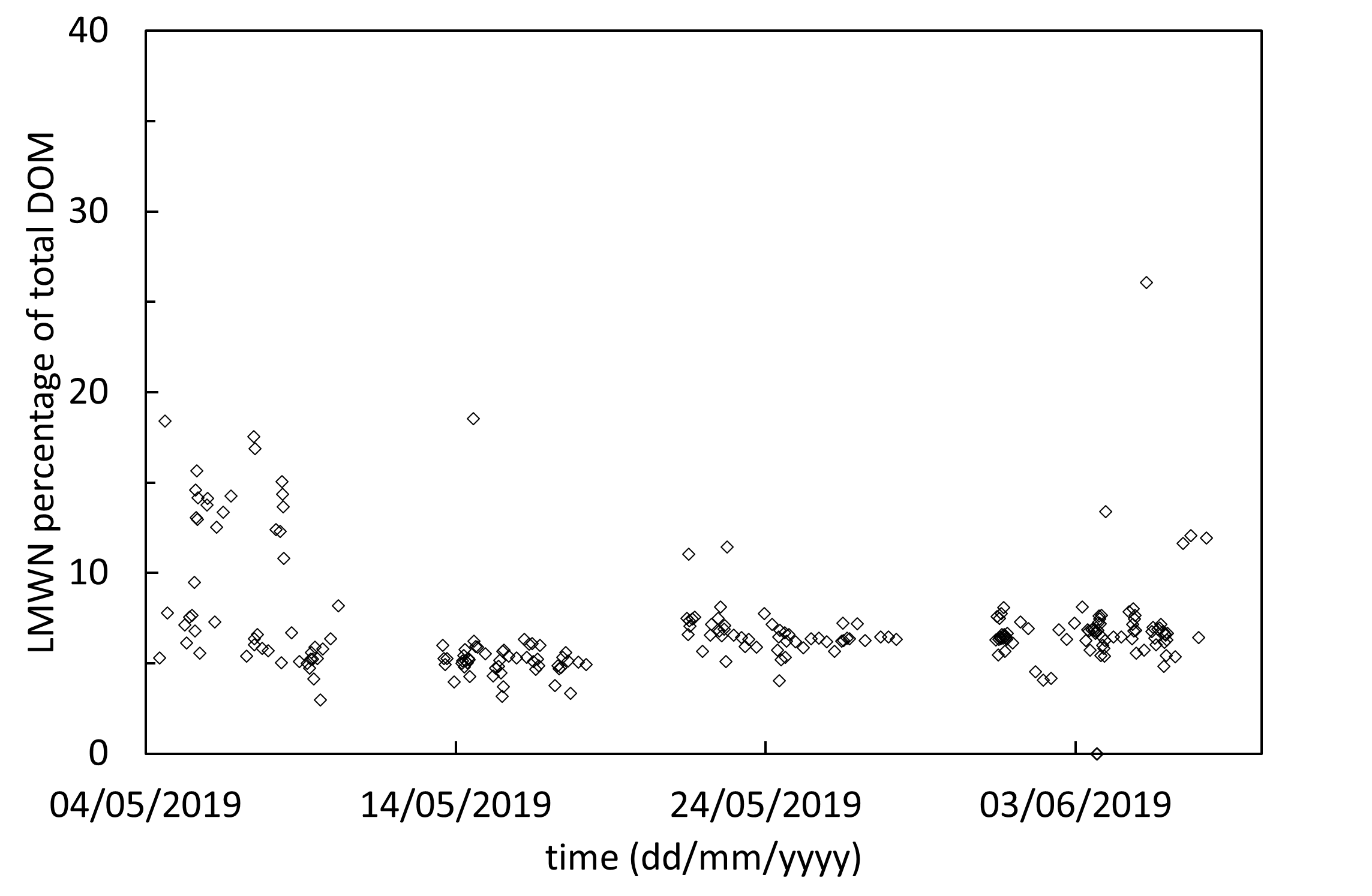


**Figure S7:** SSW temporal changes of low molecular weight neutrals percentage of total DOM


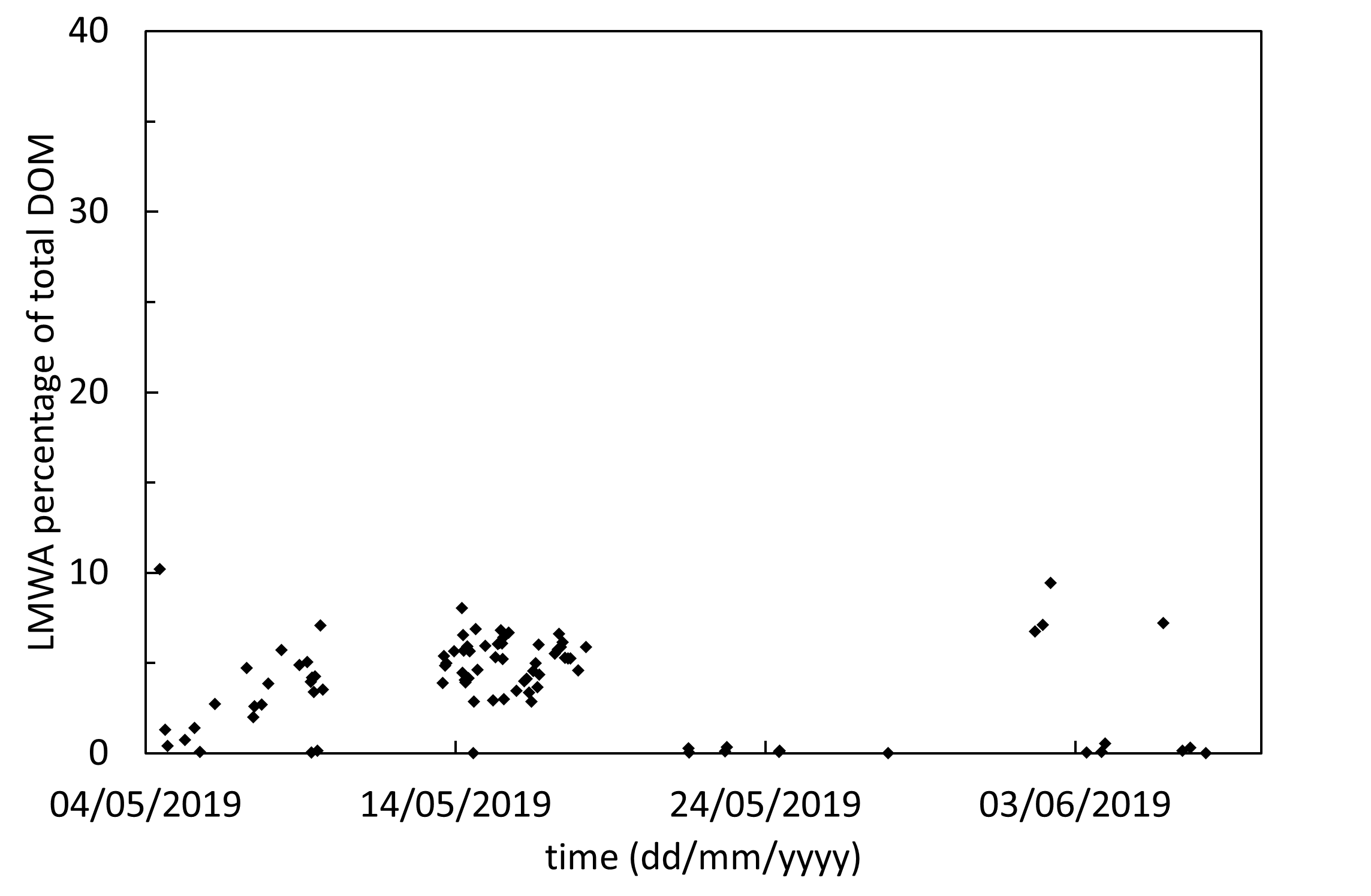


**Figure S8:** SSW temporal changes of low molecular weight acids percentage of total DOM


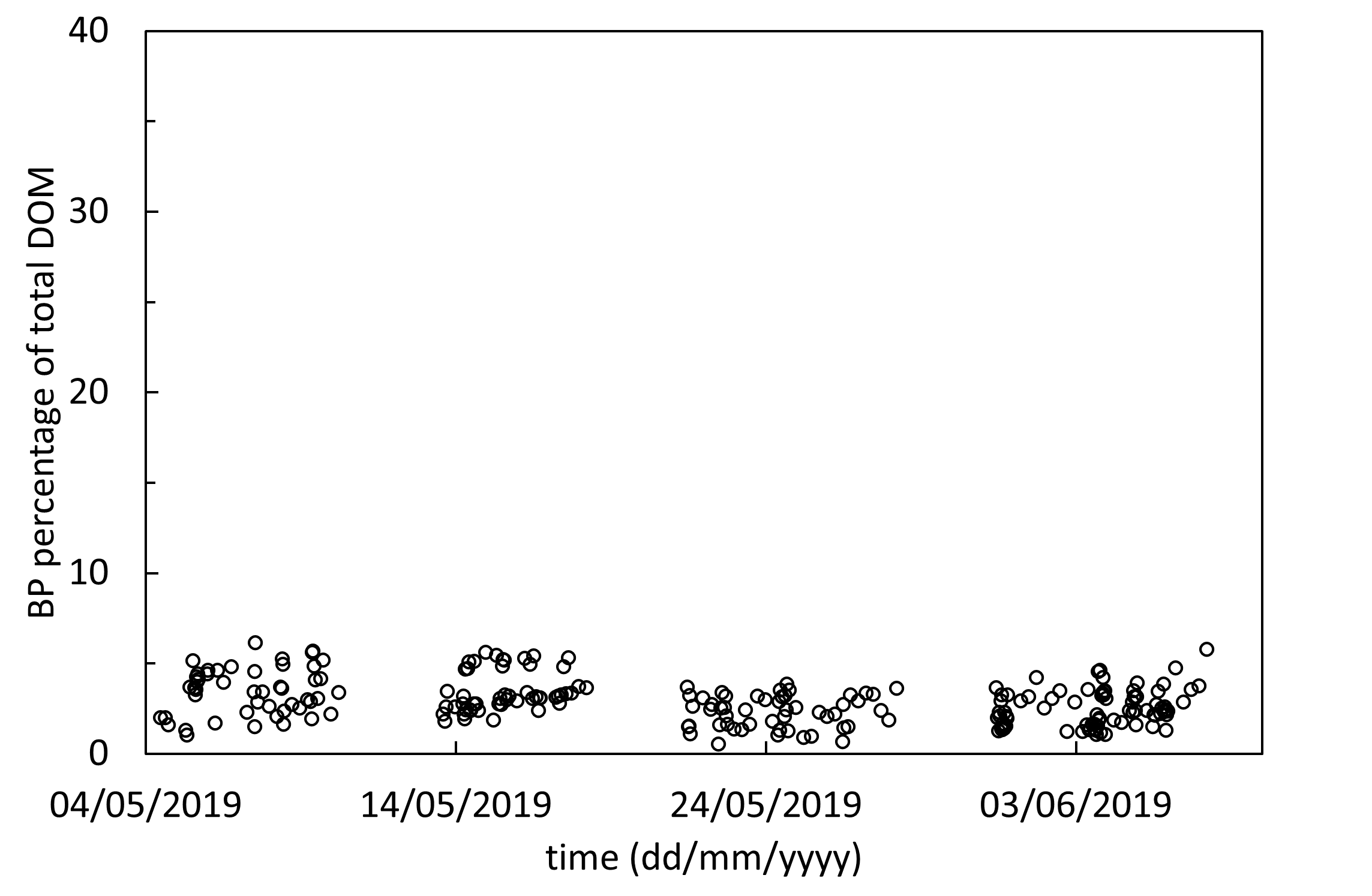


**Figure S9:** SML temporal changes of biopolymers percentage of total DOM


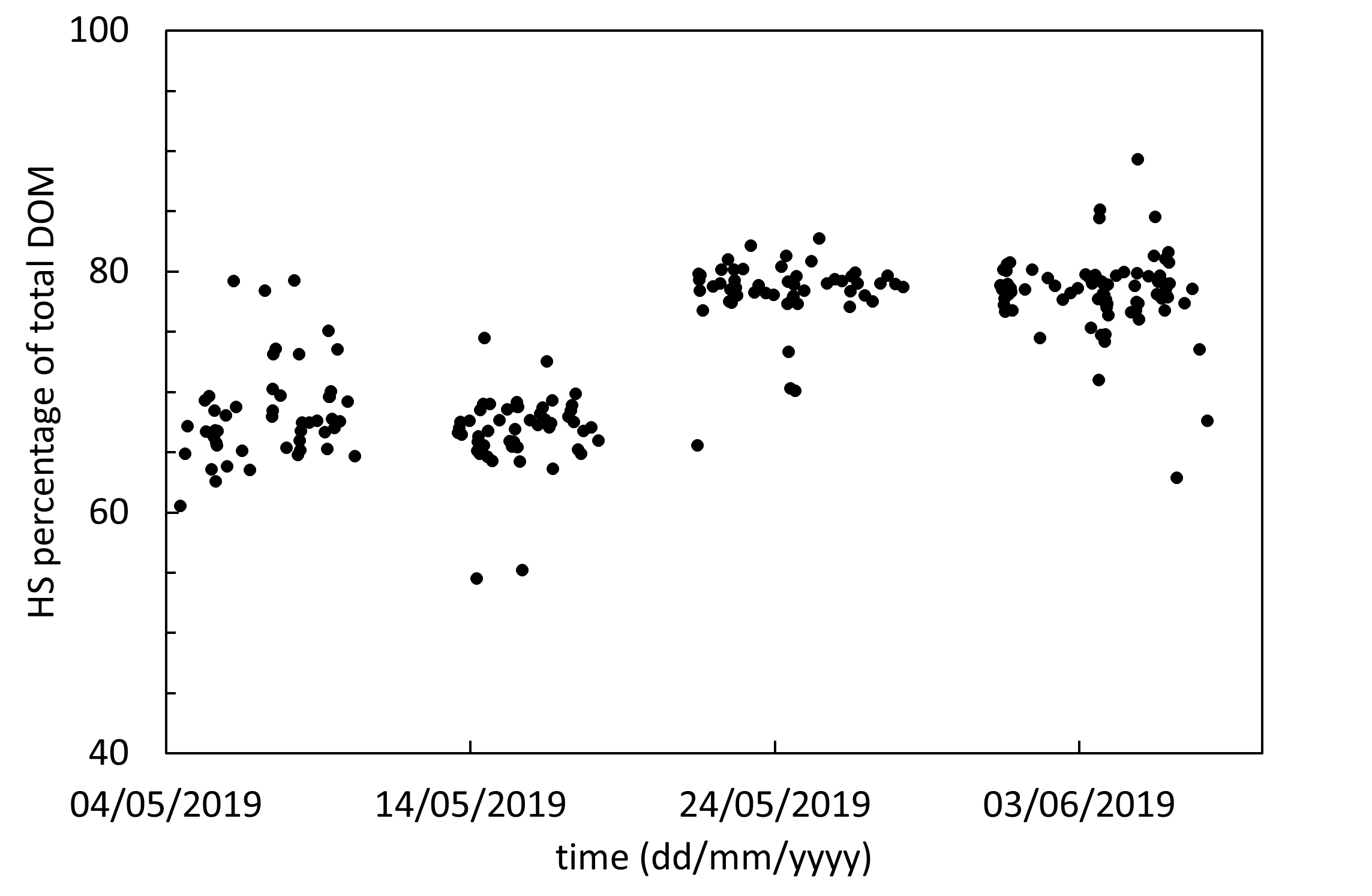


**Figure S10:** SML temporal changes of humic substances percentage of total DOM


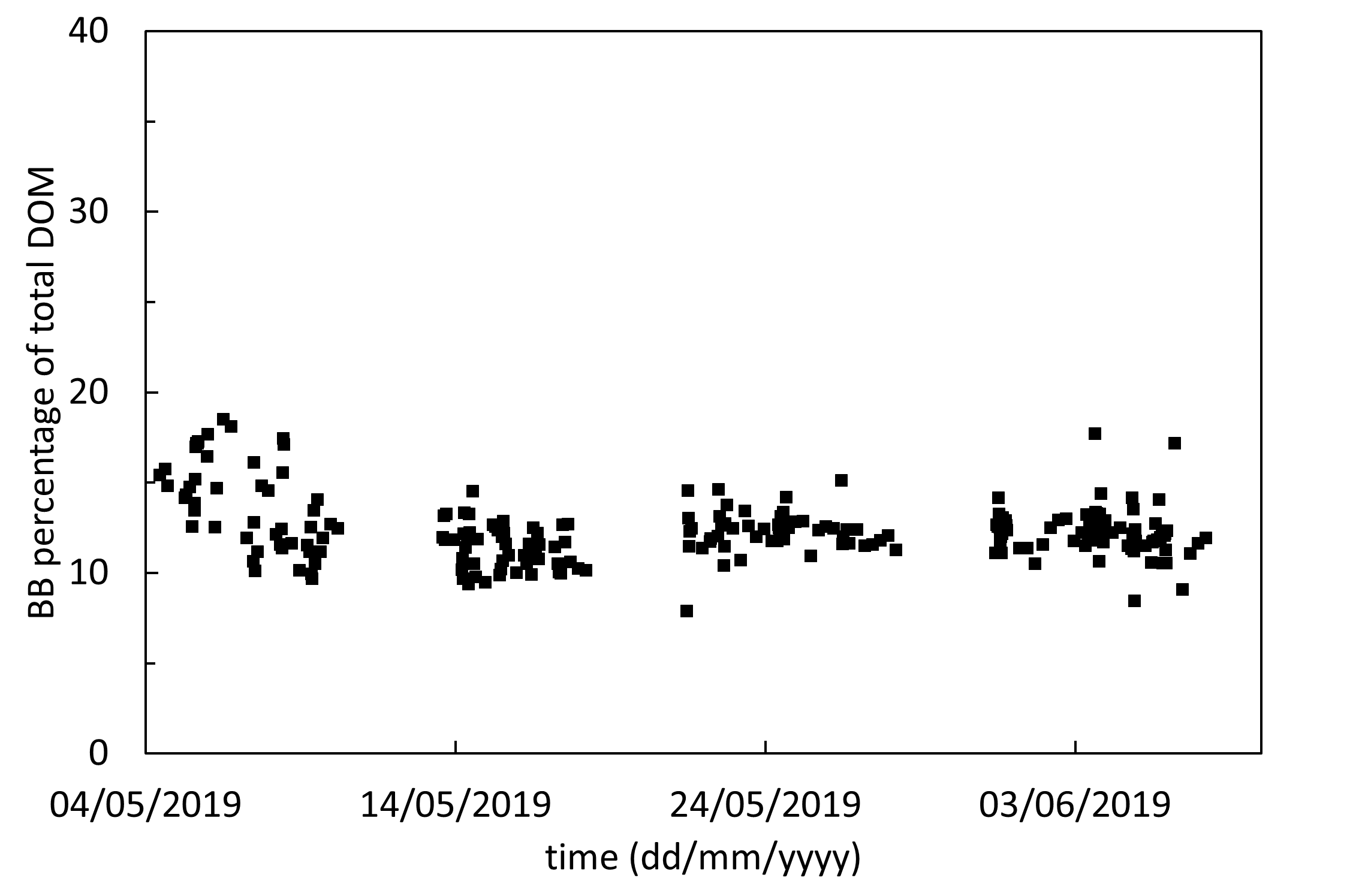


**Figure S11:** SML temporal changes of building blocks percentage of total DOM


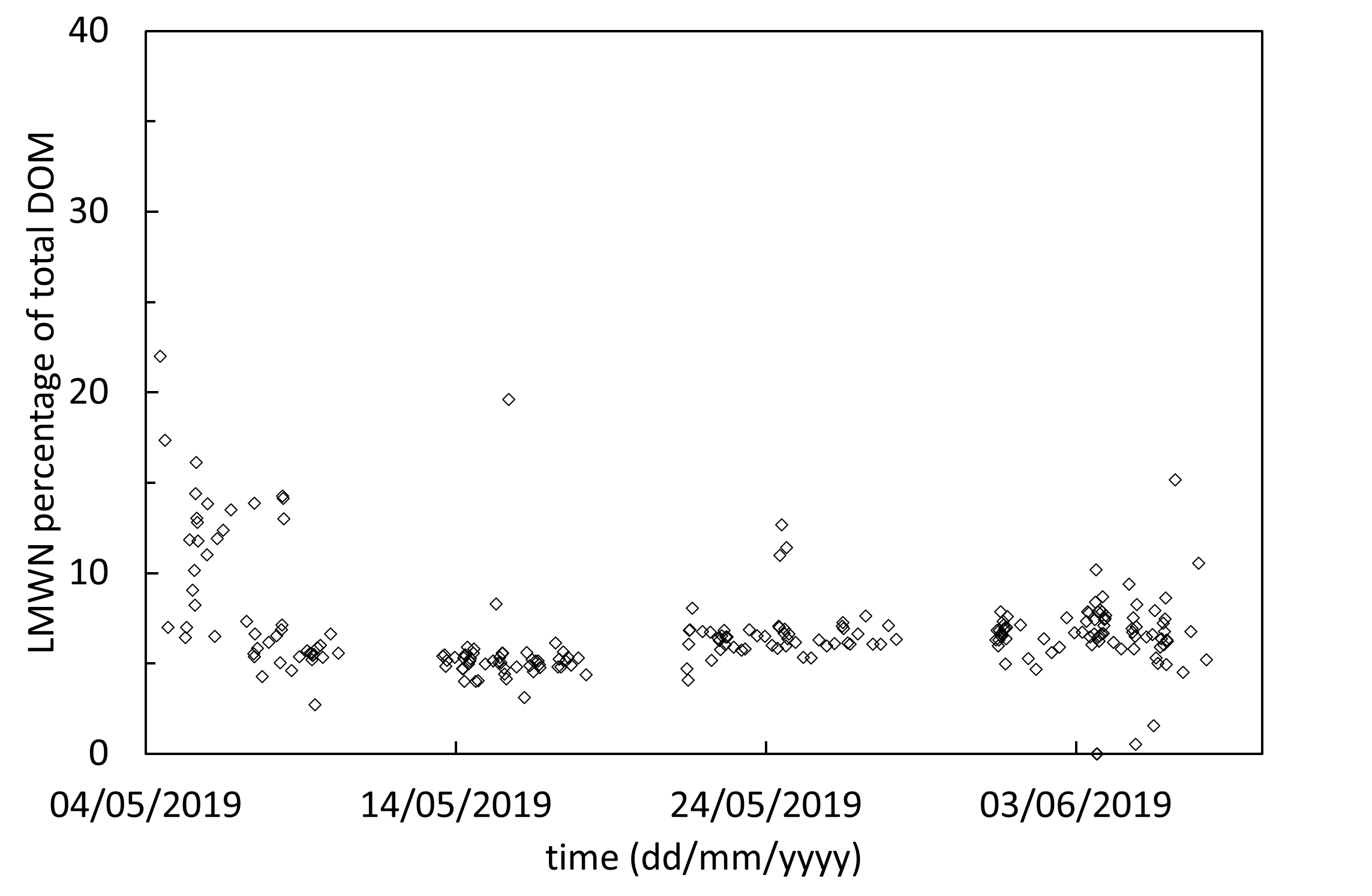


**Figure S12:** SML temporal changes of low molecular weight neutrals percentage of total DOM


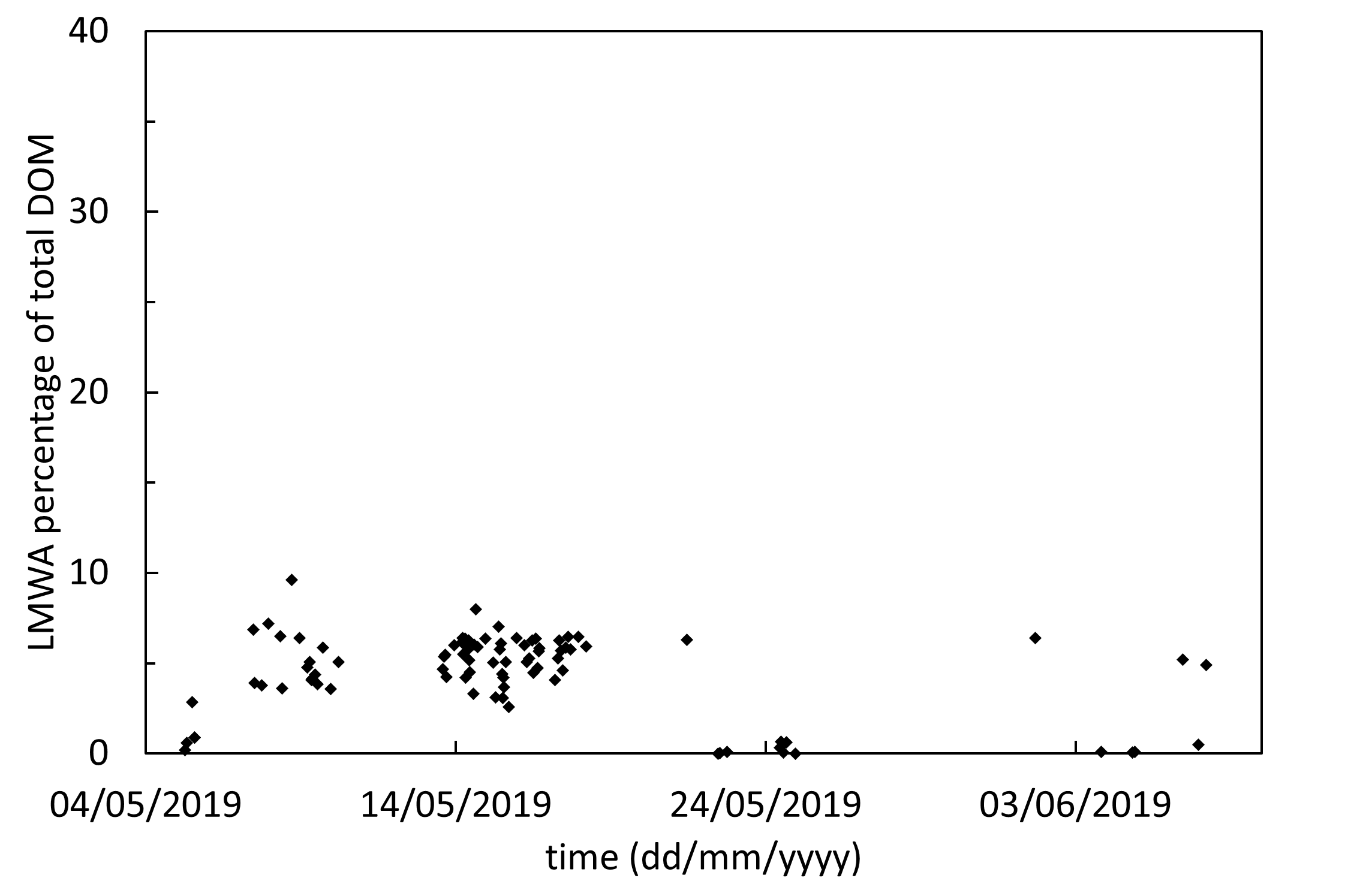


**Figure S13:** SML temporal changes of low molecular weight acids percentage of total DOM

# **G: Histogram plots of DOC and DOM composition**

*
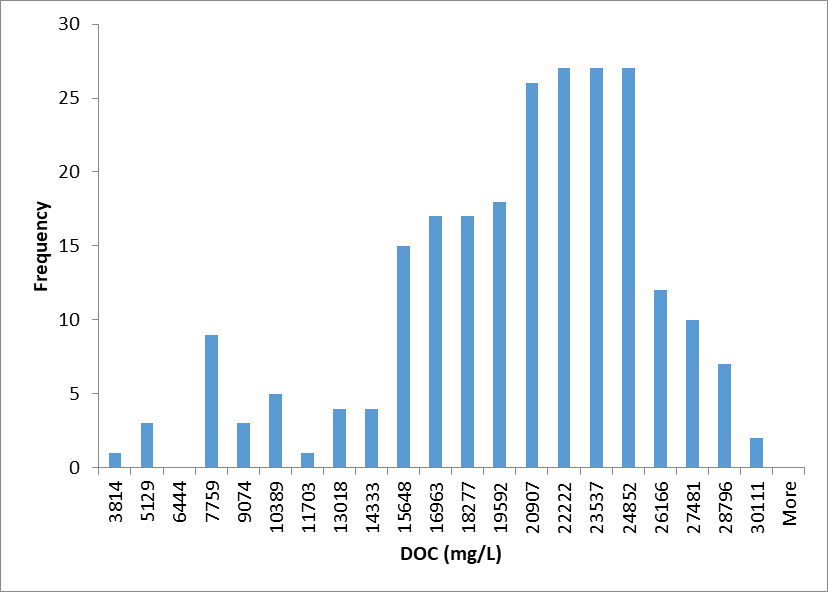
*

**Figure S14:** Histogram showing the distribution of DOC concentrations in SML during the study period.


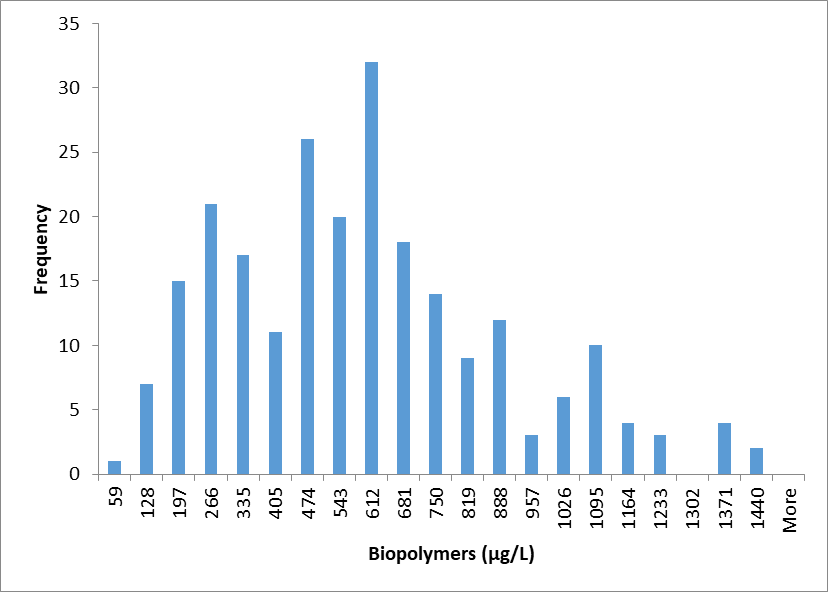


**Figure S15:** Histogram showing the distribution of biopolymers concentrations in SML during the study period.


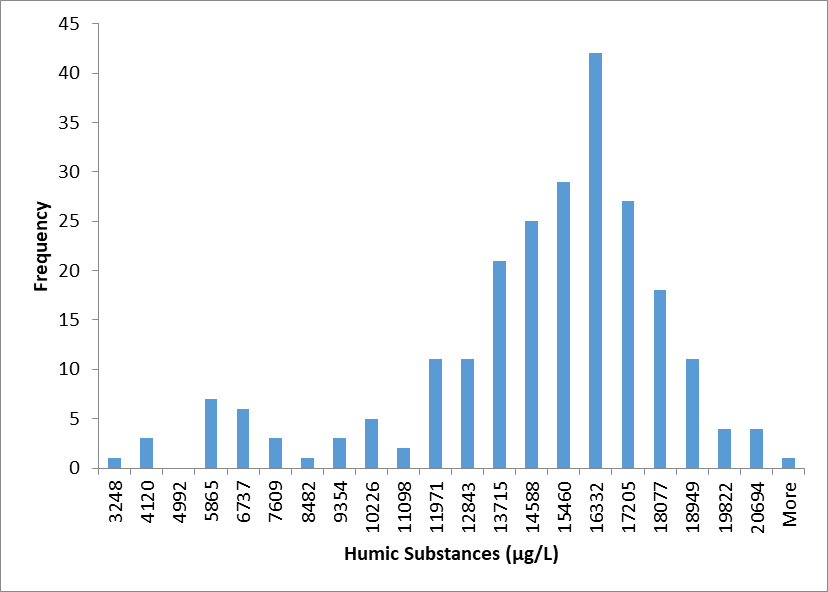


**Figure S16:** Histogram showing the distribution of humic substances concentrations in SML during the study period.


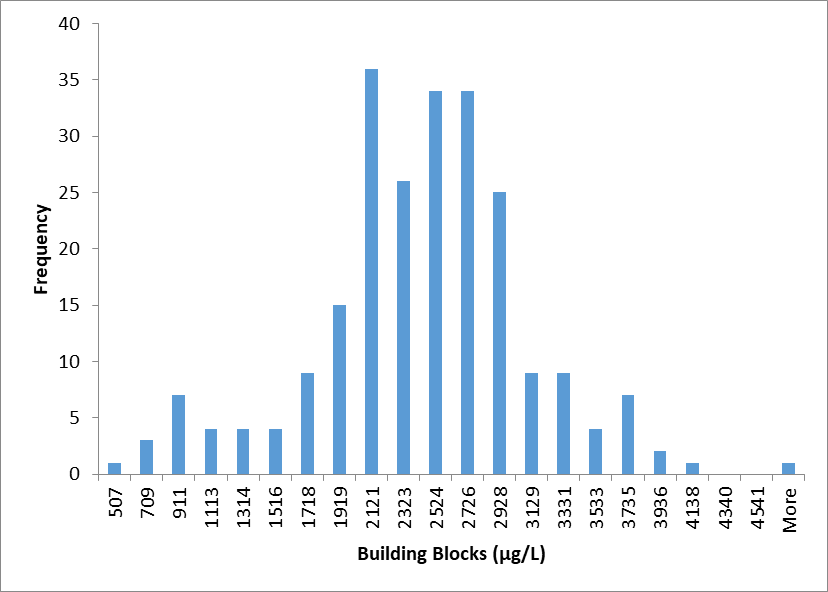


**Figure S17:** Histogram showing the distribution of building blocks concentrations in SML during the study period.


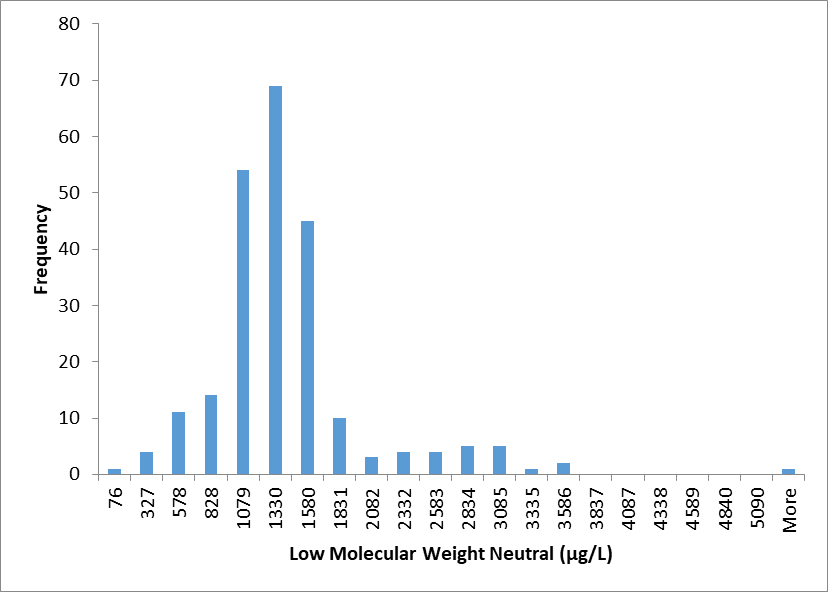


**Figure S18:** Histogram showing the distribution of low molecular weight neutrals concentrations in SML during the study period.


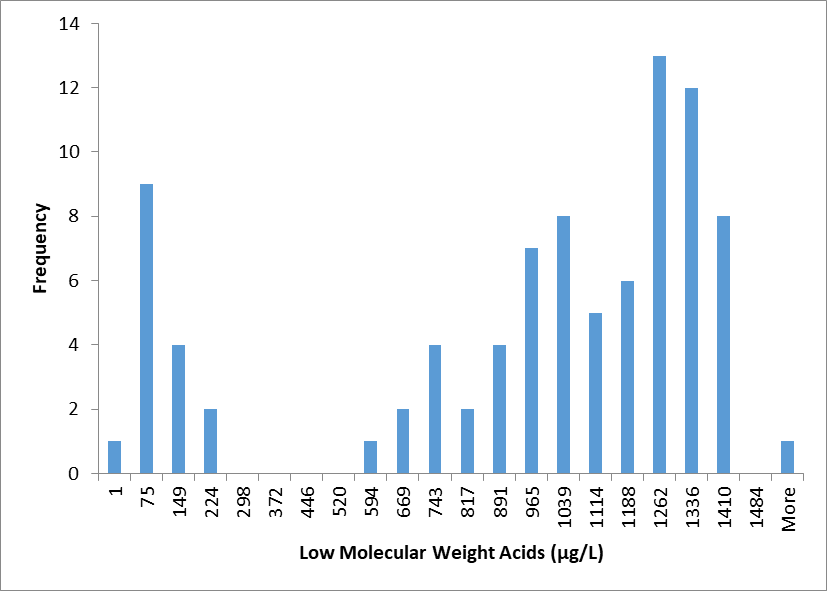


**Figure S19:** Histogram showing the distribution of low molecular weight acids concentrations in SML during the study period.


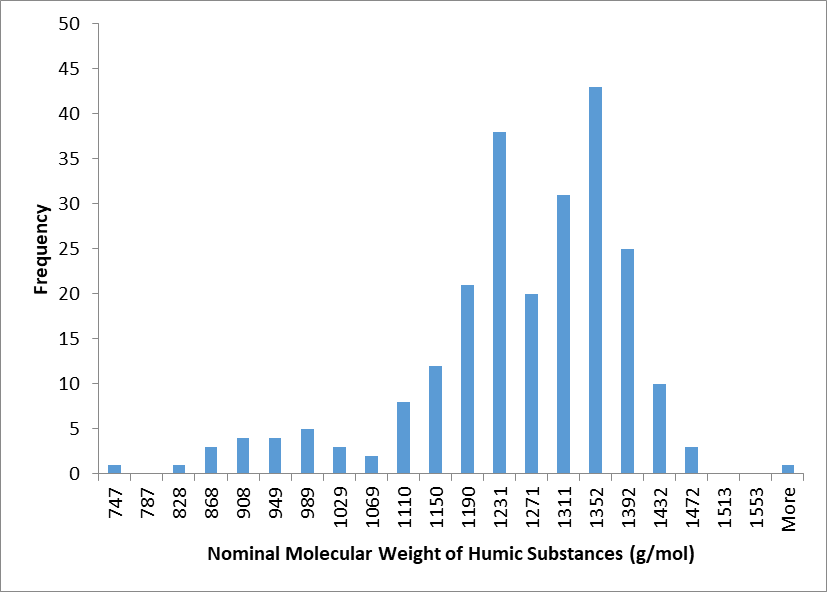


**Figure S20:** Histogram showing the distribution of nominal molecular weight of humic substances in SML during the study period.

*
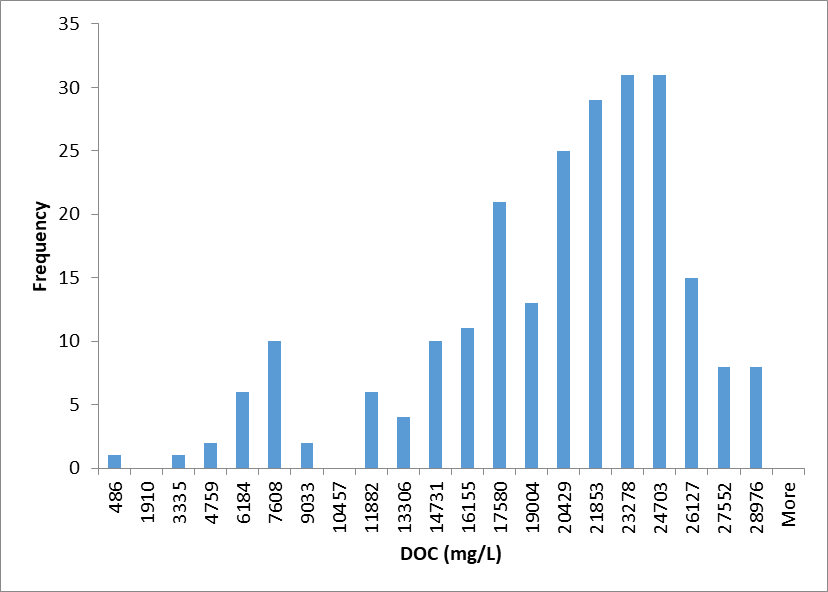
*

**Figure S21:** Histogram showing the distribution of DOC concentrations in SSW during the study period.


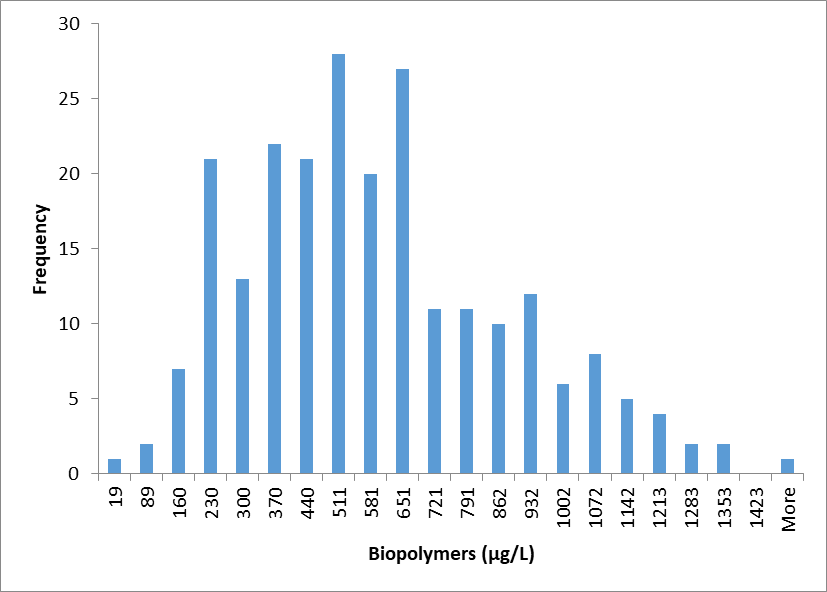


**Figure S22:** Histogram showing the distribution of biopolymers concentrations in SSW during the study period.


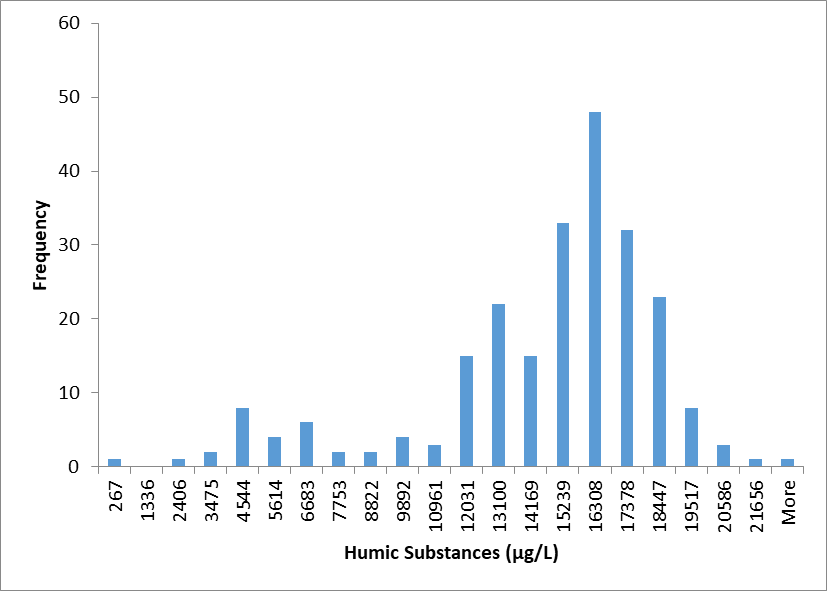


**Figure S23:** Histogram showing the distribution of humic substances concentrations in SSW during the study period.


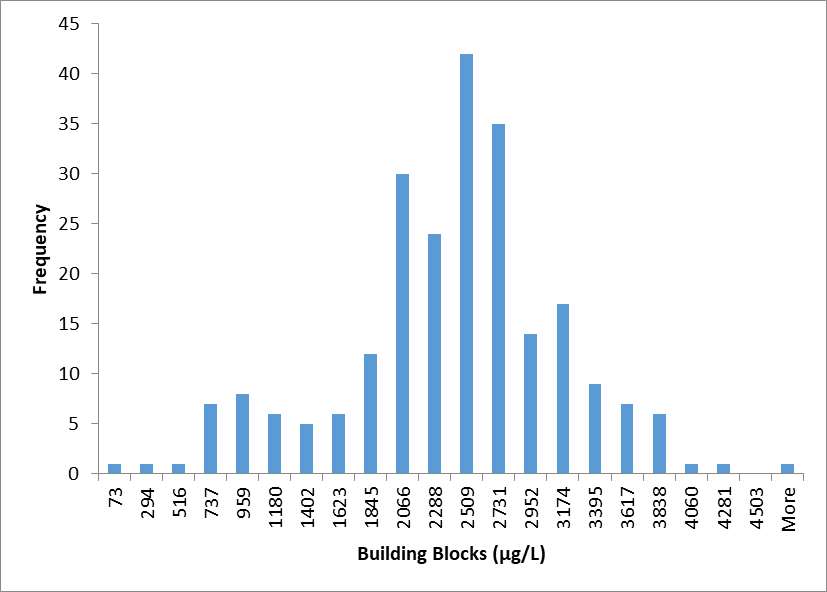


**Figure S24:** Histogram showing the distribution of building blocks concentrations in SSW during the study period.


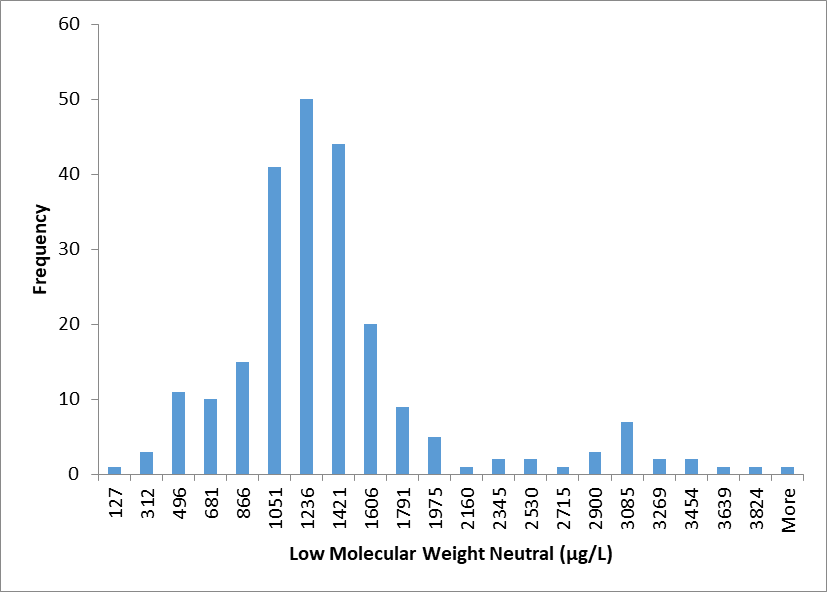


**Figure S25:** Histogram showing the distribution of low molecular weight neutrals concentrations in SSW during the study period.


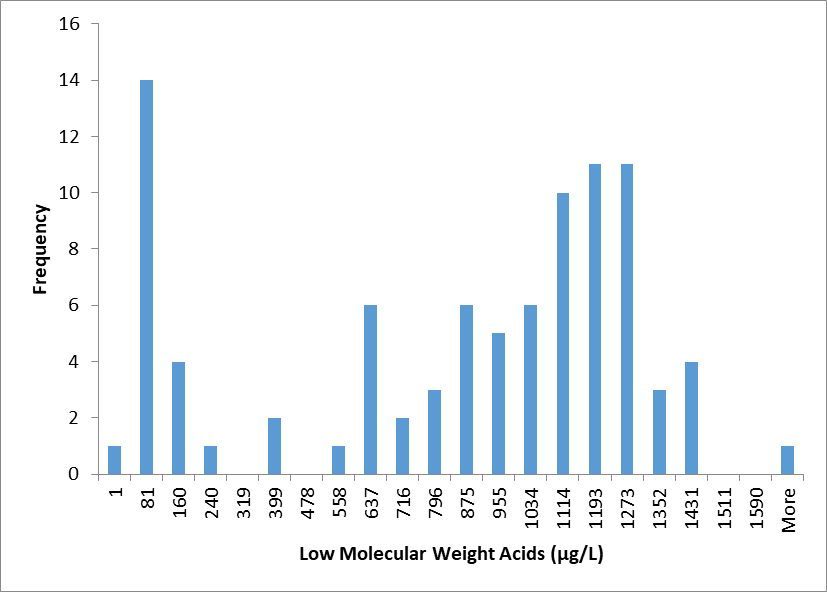


**Figure S26:** Histogram showing the distribution of low molecular weight acids concentrations in SSW during the study period.


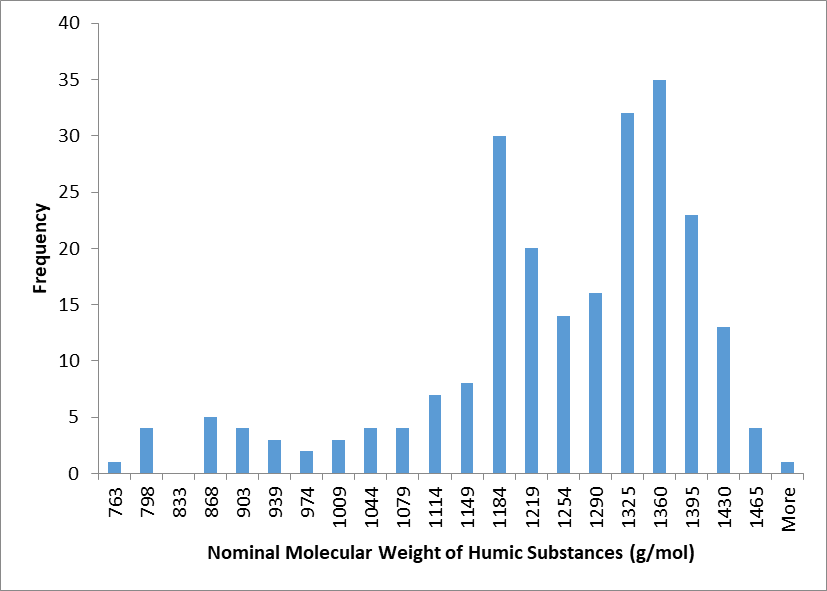


**Figure S27:** Histogram showing the distribution of nominal molecular weight of humic substances in SSW during the study period.

# **H: Humic substances diagram**

Humic substances diagram (HS-diagram): The SAC/ OC ratio (aromaticity) of aquatic humic substances is plotted against M_n_-values (nominal molecular weights), shortly expressed as molecularity.


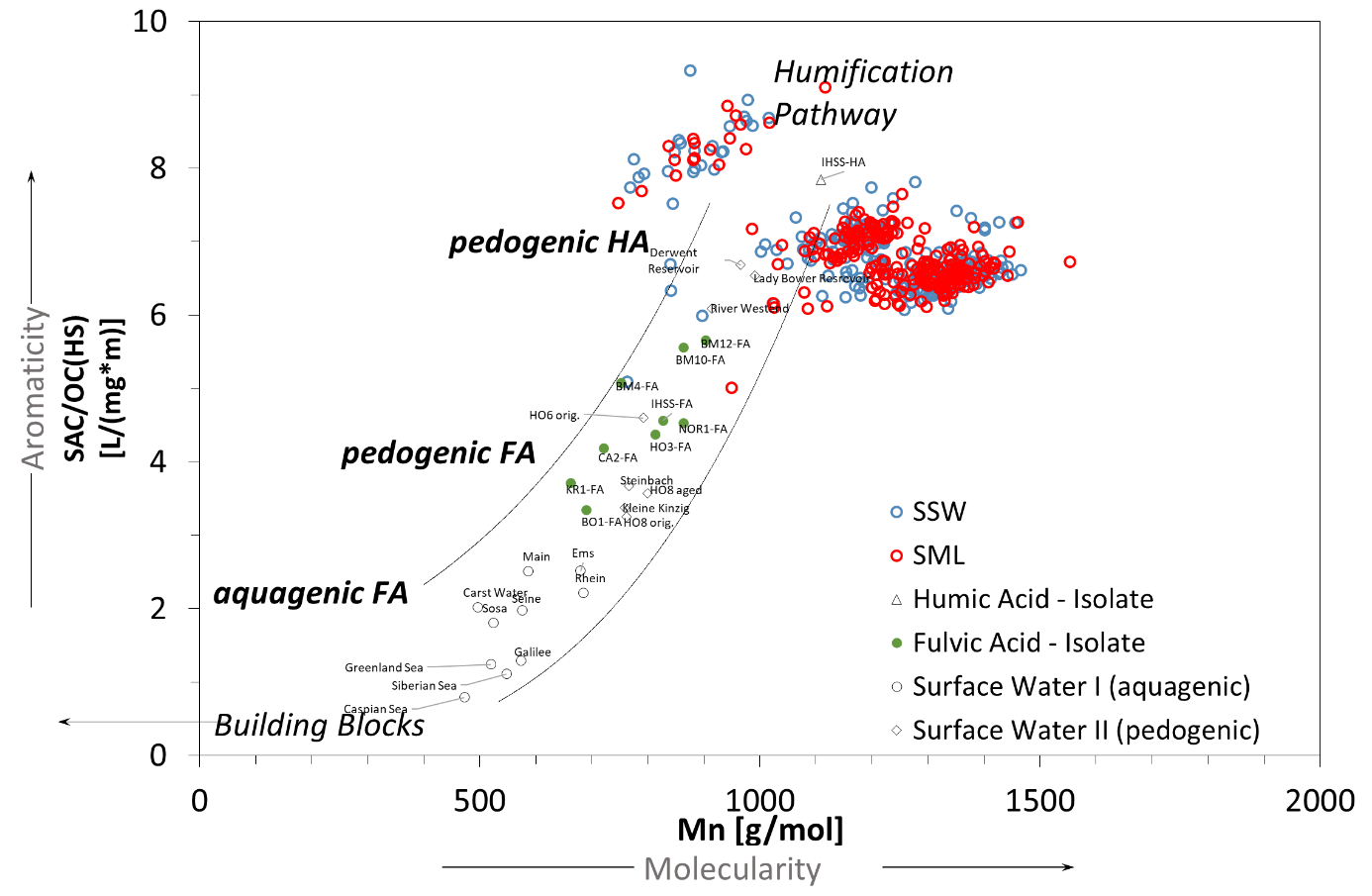


**Figure S28:** HS-diagram of SML (red) and SSW (blue)samples


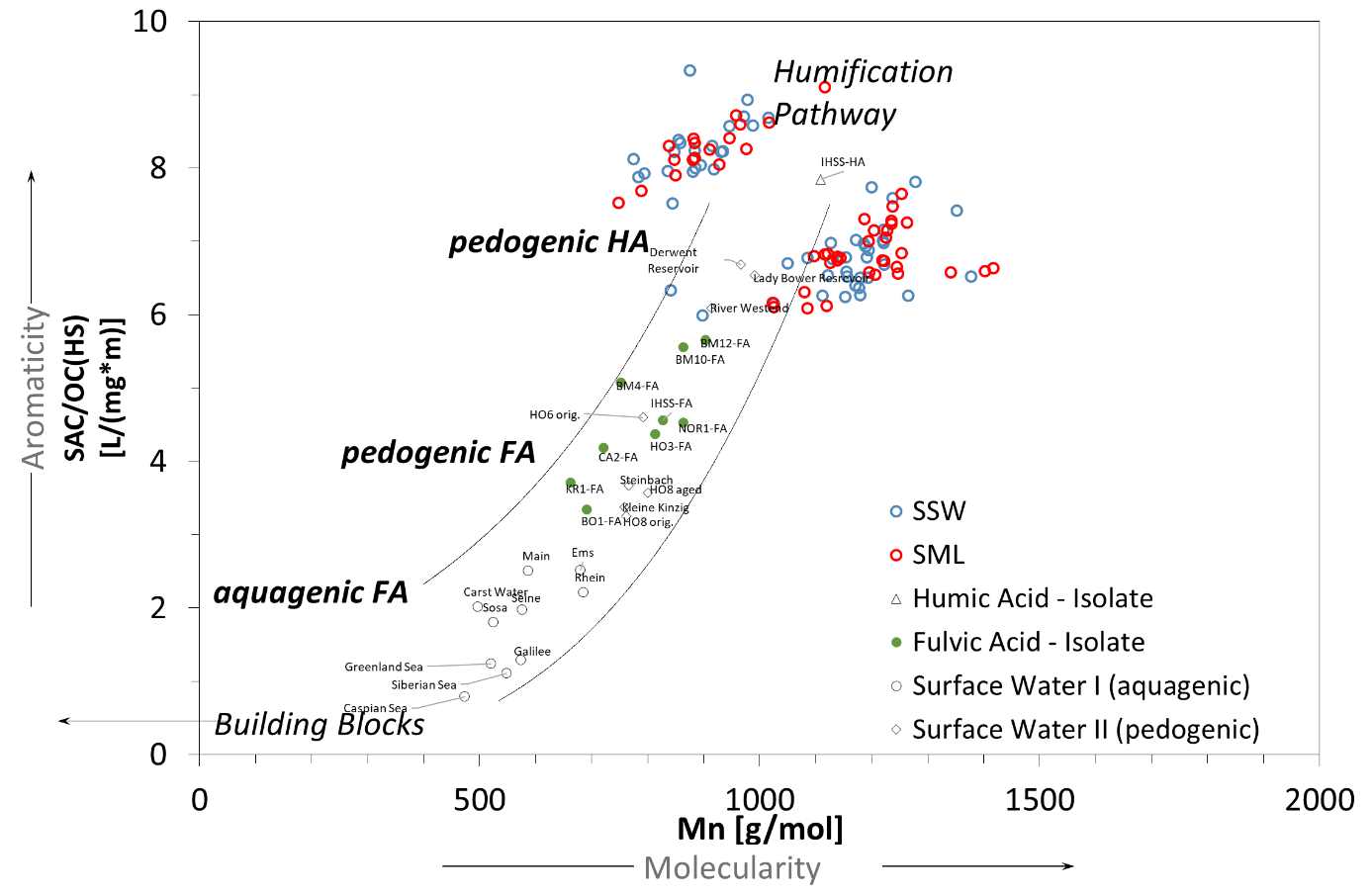


**Figure S29:** HS-diagram of SML (red) and SSW (blue)samples, Phase 1


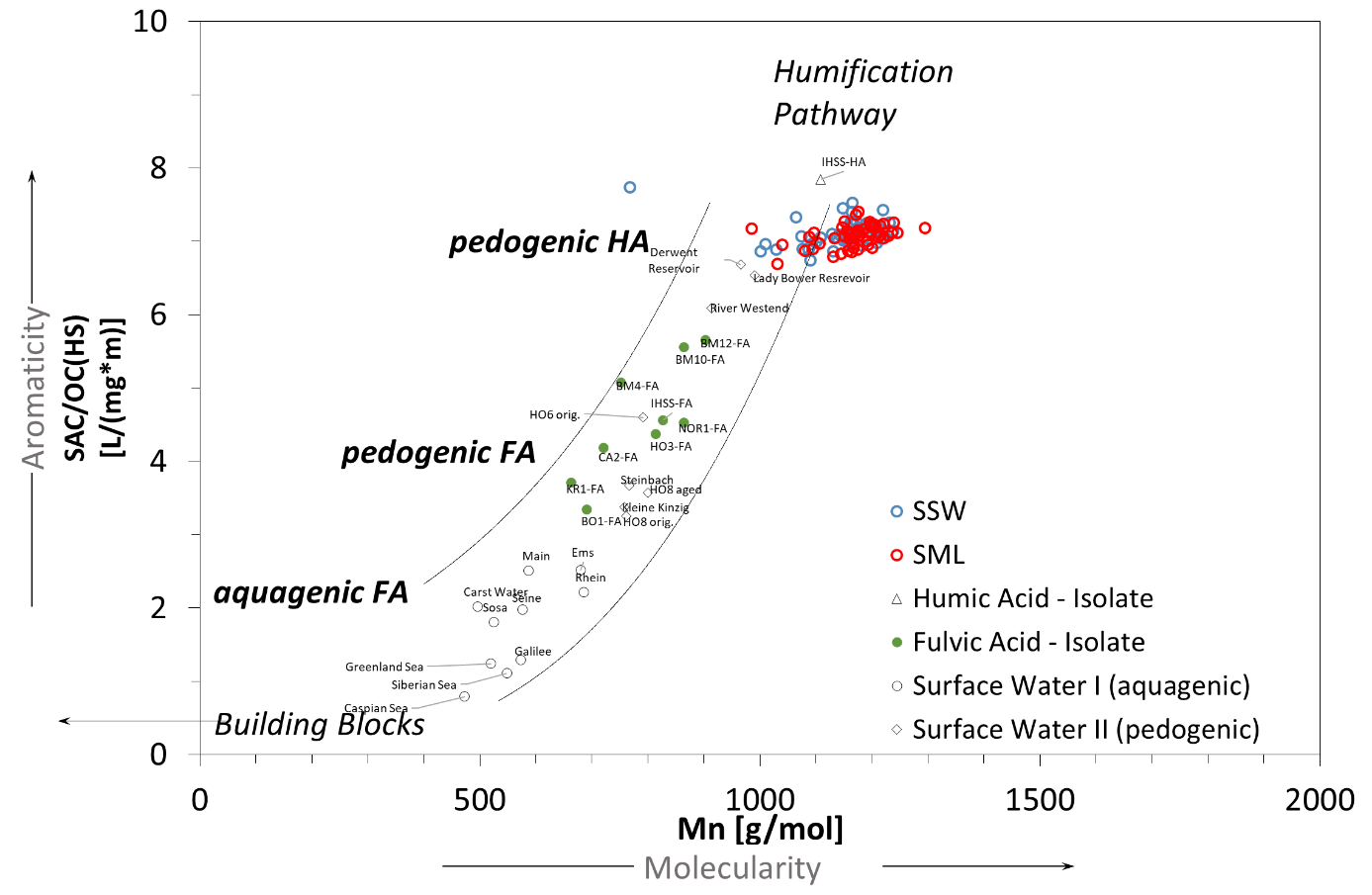


**Figure S30:** HS-diagram of SML (red) and SSW (blue) samples, Phase 2


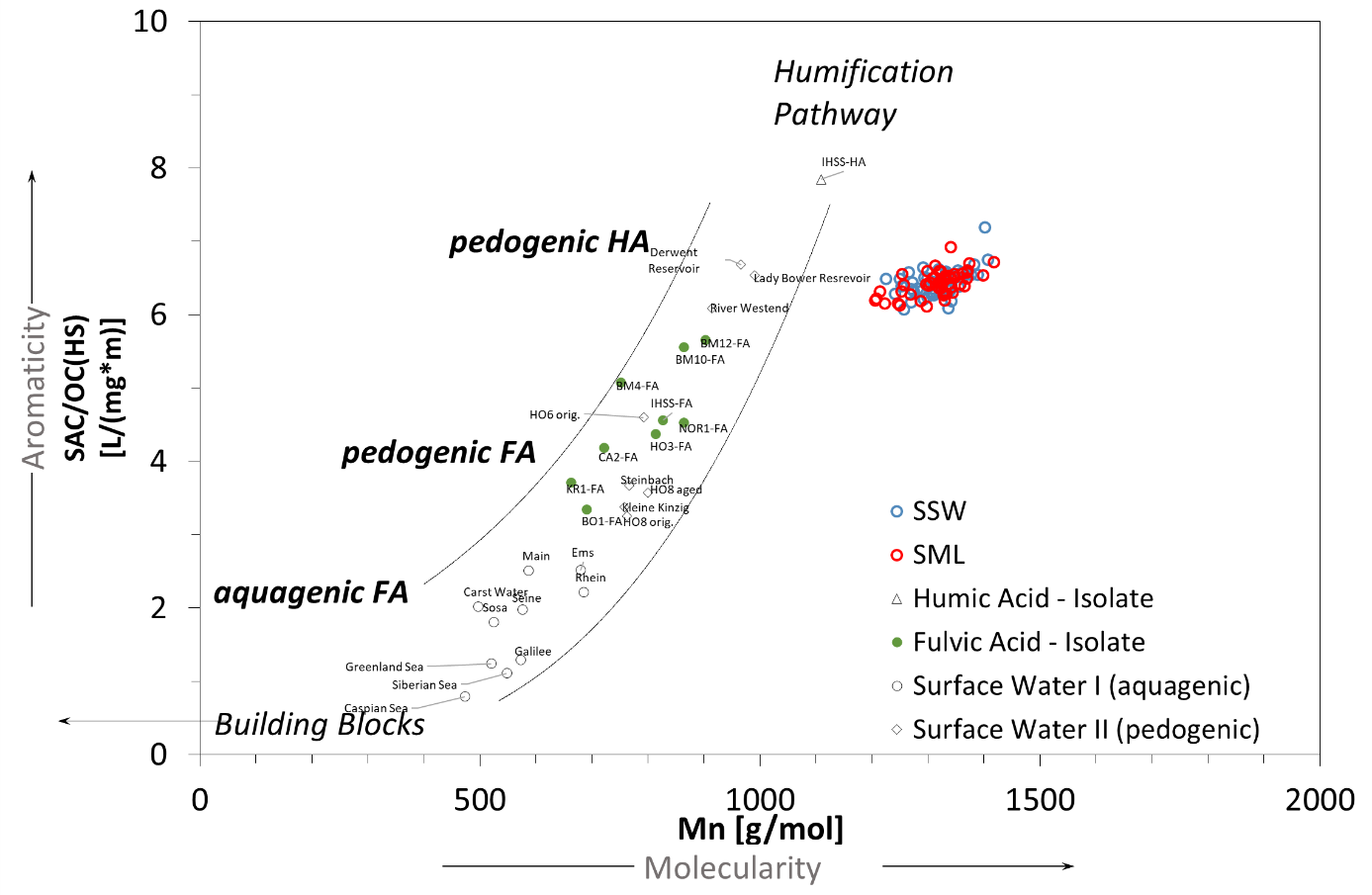


***Figure S31:*** HS-diagram of SML (red) and SSW (blue) samples*, Phase 3*


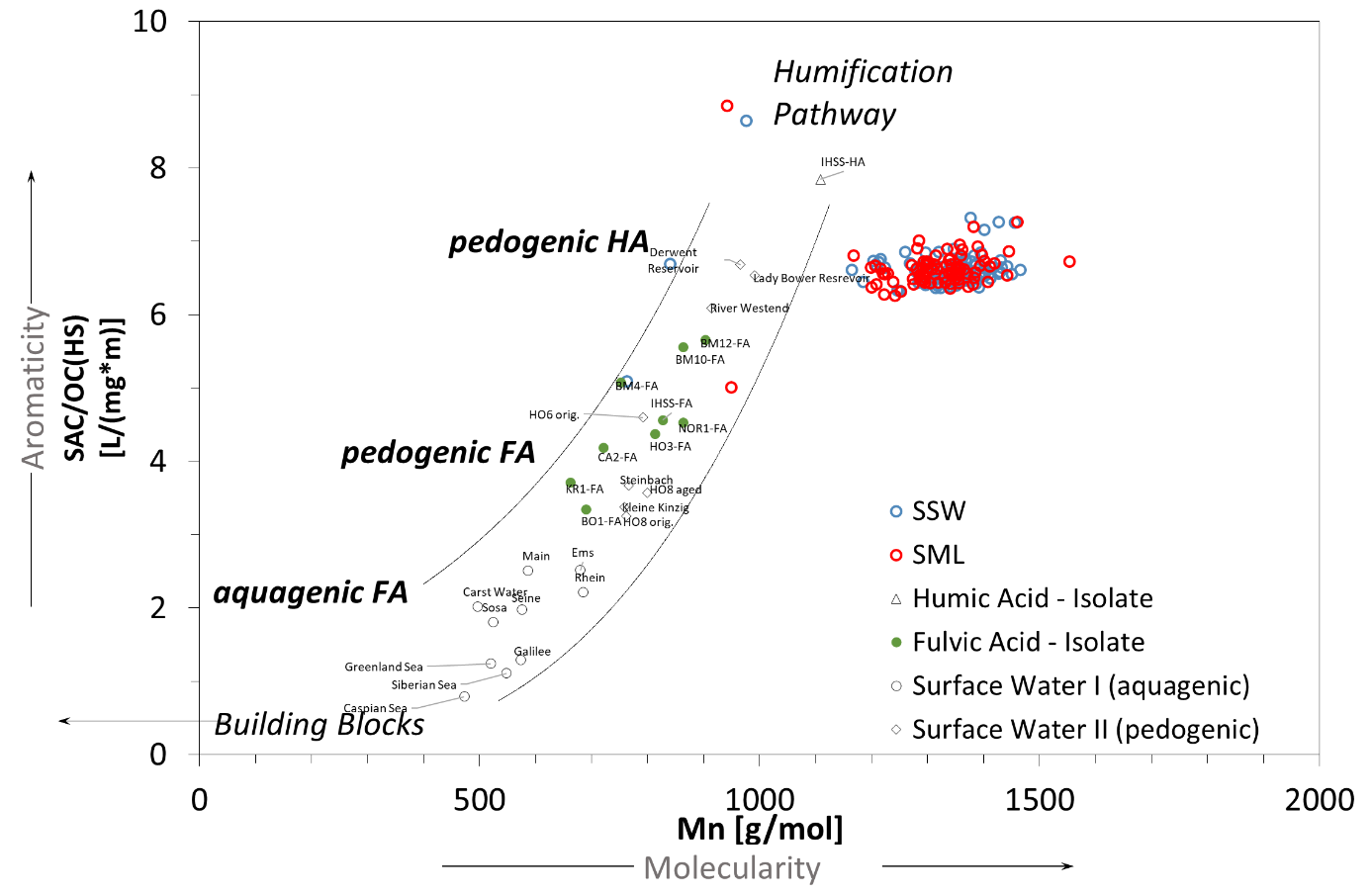


***Figure S32:*** HS-diagram of SML (red) and SSW (blue) samples*, Phase 4*
